# First Total Synthesis of Tanzawaic Acid B

**DOI:** 10.1021/acsomega.3c03634

**Published:** 2023-07-19

**Authors:** Takatsugu Murata, Hisazumi Tsutsui, Takumi Yoshida, Hirokazu Kubota, Shintaro Hiraishi, Hiyo Natsukawa, Yuki Suzuki, Daiki Hiraga, Takahiro Mori, Yutaro Maekawa, Satoru Tateyama, Kiyotaka Toyoyama, Keiichi Ito, Kyohei Suzuki, Keita Yonekura, Natsumi Shibata, Teruyuki Sato, Yasutaka Tasaki, Takehiko Inohana, Atsuhiro Takano, Naoki Egashira, Masaki Honda, Yuma Umezaki, Isamu Shiina

**Affiliations:** Department of Applied Chemistry, Faculty of Science, Tokyo University of Science, 1-3 Kagurazaka, Shinjuku-ku, Tokyo 162-8601, Japan

## Abstract

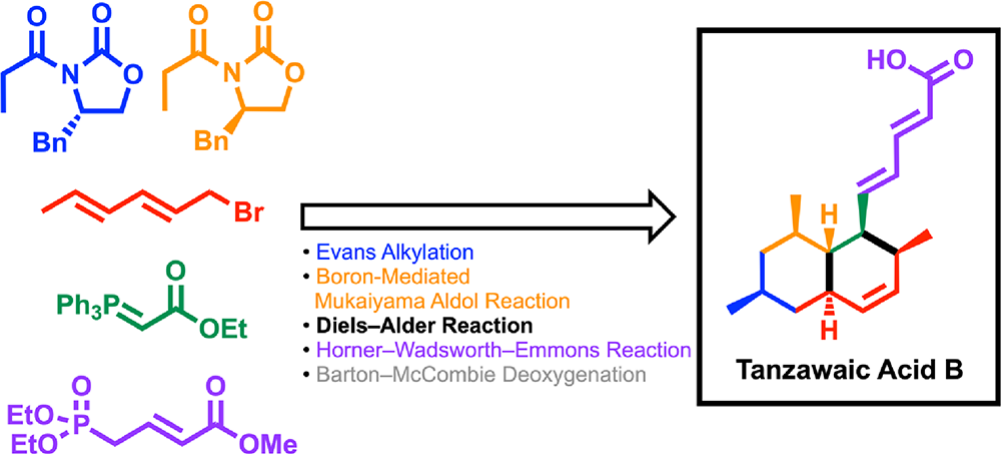

The first total synthesis of (+)-tanzawaic acid B, a natural polyketide bearing a pentadienoic ester and octalin moiety, has been accomplished. The synthetic improvement from previous synthetic conditions facilitated our gram-scale synthesis of the chiral octalin that possesses seven stereogenic centers and that is the core skeleton of almost all of the tanzawaic acid family.

In 1995, Kyowa Hakko Kogyo Co., Ltd., reported the isolation of tanzawaic acid A (GS-1302-3) (**1**) and tanzawaic acid B (GS-1302-1) (**2**) from *Penicillium* sp. GS1302^1^ (Figure 1). Two years later of the first isolation report, Uemura and co-workers reported the isolation of tanzawaic acids A and B (**1** and **2**) with other analogues of tanzawaic acids, tanzawaic acids C and D (**3** and **4**), from the different fungus, *Penicillium citrinum*,^2^ and they named the four compounds “tanzawaic” acids after a name of the Japanese toponym, “tanzawa”. After Uemura had reported the isolation of tanzawaic acids, more than 25 tanzawaic acid analogues have been isolated.^3^

**Figure 1:**
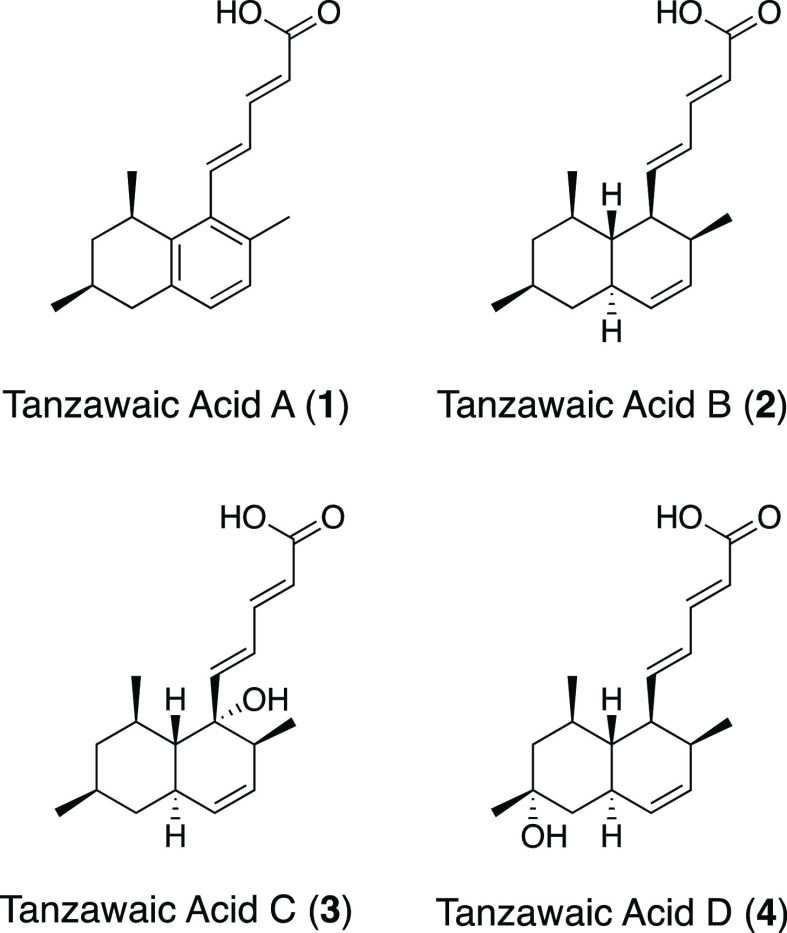
Tanzawaic acids A–D.

The biological activity of **2** had also been researched. Kyowa Hakko Kogyo Co., Ltd., reported its antibacterial activity^1^ in 1995, and Uemura and co-workers reported that **2** has an inhibition activity of superoxide production in human neutrophils induced by TPA (12-*O*-tetradecanoylphorbol-13-acetate).^2^ Since two groups had reported the biological activity, five groups have independently reported the antifungal activity,^1,3g,3n^ PTP1B inhibitory activity,^3c^ and antimalarial activity.^3k^

The structure and relative configuration of **2** were first suggested by Kyowa Hakko Kogyo Co., Ltd. However, they had not shown the information on the absolute configuration of **1** and **2**. Arimoto and Uemura and co-workers accomplished the total synthesis of **1** and revealed the absolute configuration of **1**.^4^ The contribution of their work expected us that the absolute configuration of **2** was corresponding stereochemistry to the one of **1**. In 2018, Guo and Li groups confirmed the structure and the absolute configuration of **2** from X-ray crystallography.^3i^

While there have been a number of isolation and biological researches, there has never been a synthetic study of tanzawaic acid B (**2**). Therefore, we initiated the synthetic study of **2** with the aim of establishing a method to supply **2**, and the details are presented in this paper (Figure 2).

**Figure 2:**
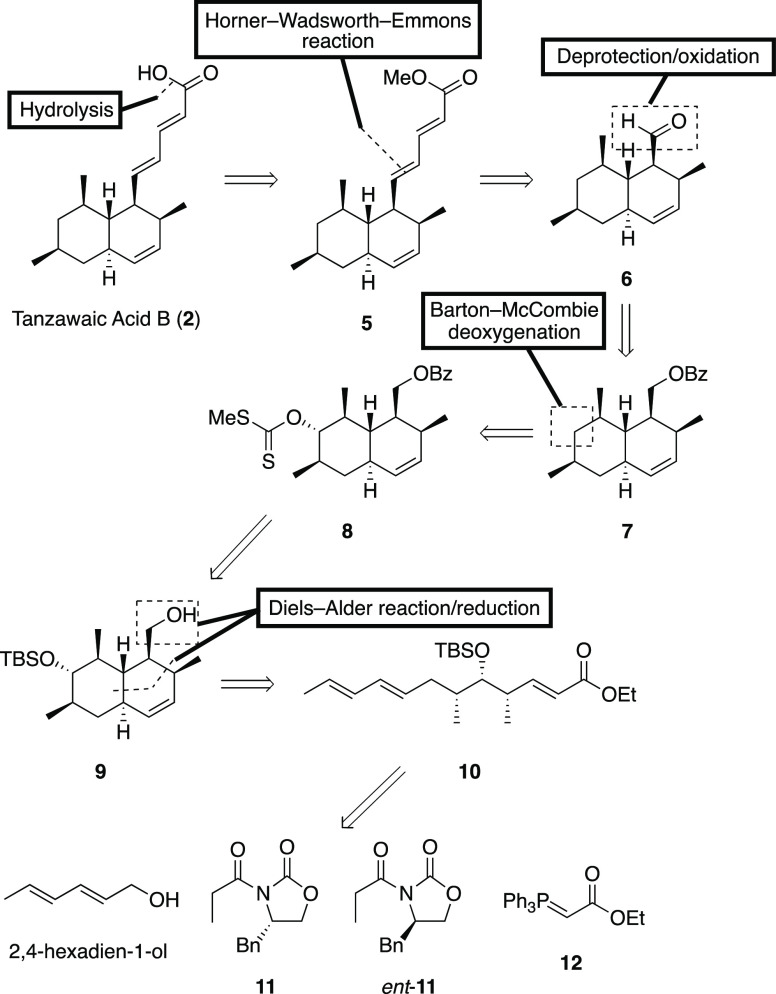
Retrosynthetic analysis of tanzawaic acid B (**2**).

## Results and Discussion

Tanzawaic acids are structurally composed of a pentadienoic acid moiety and a multi-substituted octalin skeleton bearing six stereogenic centers. We had previously reported the total synthesis of coprophilin^5^ that contains a similar skeleton with tanzawaic acids. So, we planned to start this study with a strategy for the utilization of the chiral skeleton.

Tanzawaic acid B would be obtained from the corresponding ester **5**, following the Horner–Wadsworth–Emmons (HWE) reaction of aldehyde **6**. The aldehyde could be prepared from xanthate **8** through the Barton–McCombie deoxygenation and several functional transformations. We assumed that the radical reduction reaction should be conducted before elongation to prevent from geometric isomerization. The xanthate would be given by the protection/deprotection sequence of two hydroxyl groups from the key intermediate **9**. Octalin **9** could be obtained from 2,4-hexadien-1-ol, **11**, *ent*-**11**, and **12** by reference to our previous report.^5,6^

As discussed above, we had already prepared the chiral octalin in less than gram-scale. However, the gram-scale synthesis of key intermediate **9** was needed for the synthetic study of tanzawaic acid, which is a reason for considering a number of steps after the construction of the octalin unit. Therefore, we began to start the gram-scale synthesis of octalin **9**, which bears the seven stereogenic centers, and improve our synthetic route at first.

**Scheme 1:**
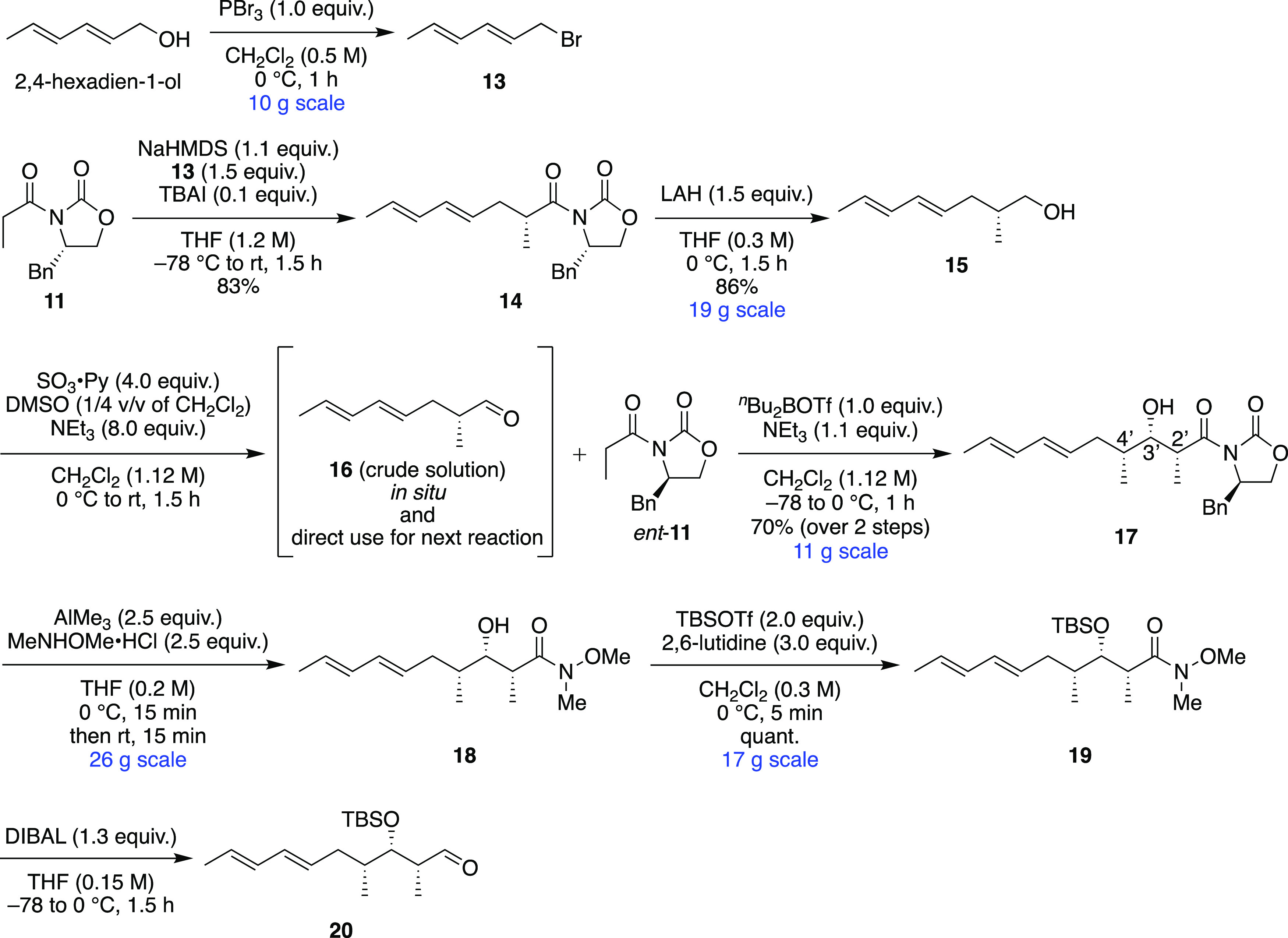
Synthesis of Weinreb Amide **19**

At the beginning of the synthesis, we improved the synthetic conditions to Weinreb amide **19** (Scheme 1). Commercially available 2,4-hexadien-1-ol was brominated to dienyl bromide **13** with simple purification,^7^ and it was used for the Evans alkylation reaction with chiral auxiliary **11** to afford alkylation product **14**. After the reductive removal of the chiral auxiliary of **14** by lithium aluminum hydride, alcohol **15** was subjected to the conditions of the Parikh–Doering oxidation to give the crude solution of the chiral and α-epimerizable aldehyde **16**. The reaction mixture of **16** was directly sent by a cannula into the other enolate system, which was prepared from *ent*-**11** with dibutylboron trifluoromethanesulfonate and triethylamine, to give all-*syn*-adduct **17** in over decagram-scale. This two-sequence reaction system was necessary for the Mukaiyama boron-mediated aldol reaction to afford all-*syn*-adduct **17**. In the case of using isolated aldehyde for the aldol reaction, the epimerized aldol product 4′-epi-**17** was detected, which is not non-Evans-*syn* adduct 2′-epi-3′-epi-**17**.^8^ All-*syn*-adduct **17** was led to Weinreb amide **18**, and then its free hydroxyl group was protected by the *tert*-butyldimethylsilyl (TBS) group. The reductive removal of the Weinreb amide group was delivered by diisobutylaluminum hydride (DIBAL) to afford aldehyde **20**. Since we could obtain aldehyde **20** with enough amounts, we conducted the gram-scale synthesis of enantiomeric octalin **9** with more improvement than the small amount synthesis in the previous report.^5^

**Scheme 2:**
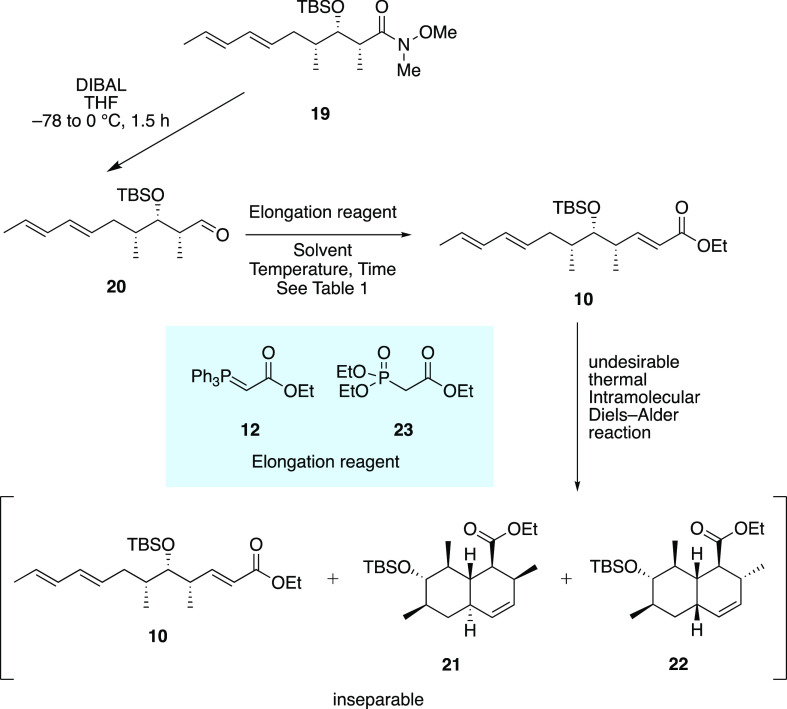
Undesirable Thermal IMDA Reaction

Aldehyde **20** was subjected to the Wittig reaction conditions or HWE reaction conditions (Scheme 2 and Table 1), but the thermal intramolecular Diels–Alder (thermal IMDA) reaction proceeded in a gram-scale with a low *trans*-octalin **21** selectivity (entry 1). Therefore, we had to investigate more effective conditions from **20** to **9**.^9^ To suppress the thermal IMDA reaction, we performed the corresponding HWE reaction, which is a more kinetic Wittig reaction and can be realized at a lower temperature than the Wittig reaction. As a result, the yield of **10** was increased and the yields of IMDA adducts were suppressed, but the geometric isomer of **10** was also given in an 18% yield (entry 2). Thus, we investigated the solvent effect on the Wittig/thermal IMDA reactions, but no solvent effect against selectivity in the Diels–Alder reaction step was observed (entries 3–5 and 7). Hence, we analyzed that this thermal IMDA reaction was dependent only on the reaction temperature. As a result, 35 °C was the highest temperature at which we could get the Wittig product efficiently without proceeding with the thermal IMDA reaction (entry 6). The temperature could also be explained using the Arrhenius equation by calculating the activation energy from the yield of the Wittig product.

**Table 1:** Effect of Solvent and Temperature in the Wittig Reaction toward the Thermal IMDA Reaction

					yield (%) (2 or 3 steps from **20**)			
entry	elongation reagent	solvent	temperature	time	**10**	**21**^a^	**22**^a^	rSM (**20**)
1^b^	**12**	toluene	110 °C	1.5 h	51	19	16	0
2	**23**^c^	THF	–78 °C to rt	14 h	63^d^	0	0	0
3	**12**	THF	rt	16 h	59	0	0	0
4	**12**	THF	66 °C	14 h	78	3	2.5	23
5	**12**	CH_2_Cl_2_	rt	14 h	56	0	0	28
6^b^	**12**	CH_2_Cl_2_	35 °C	14 h	87	0	0	trace
7	**12**	CH_2_Cl_2_	40 °C	16 h	83	trace	trace	0

a Based on the calculation from the ^1^H NMR spectra.

b Performed on over 10 g scale.

c Lithium bis(trimethylsilyl)amide was used as the base for deprotonation.

d The geometric isomer was also given in an 18% yield.

**Scheme 3:**
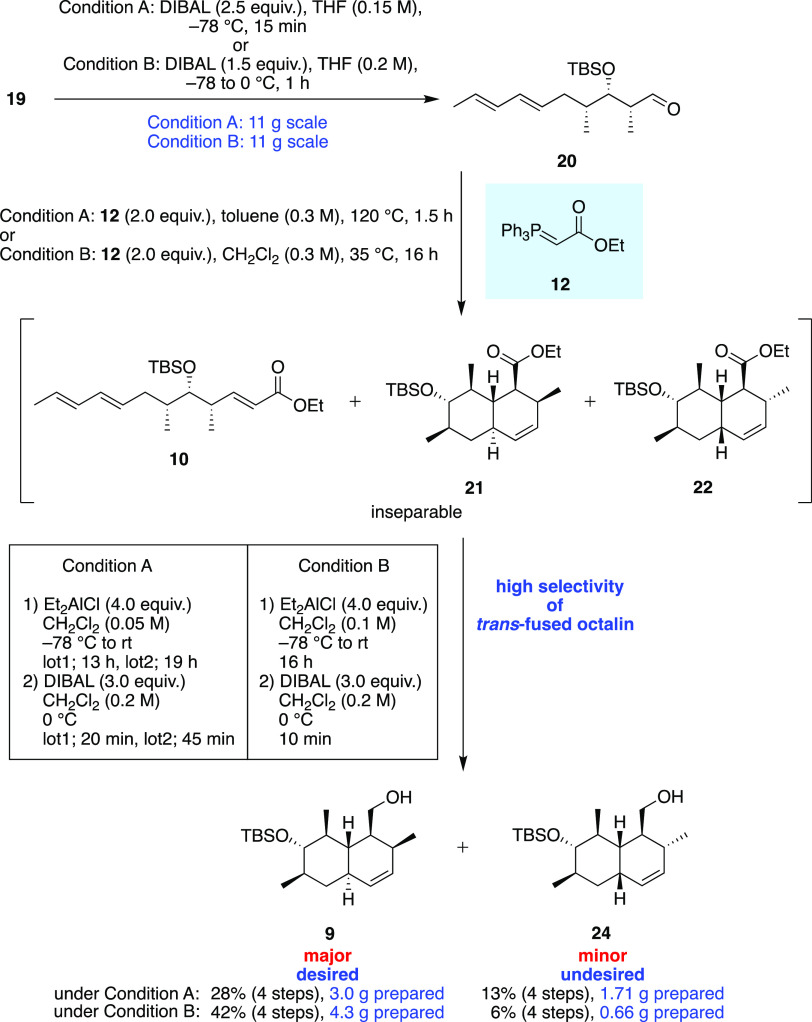
Comparison of Four-Step Yields in Decagram-Scale Synthesis

According to the above investigation, we compared the yields of **9** from **20** over four steps in the Wittig conditions between Table 1 entries 1 and 6 (Scheme 3). In the case that condition A at high temperatures in toluene was employed for the Wittig reaction, octalin **9** was given in 28% yields over four steps and the undesired *cis*-fused octalin **24** was given in 13% yields over four steps. On the other hand, in the case that condition B at moderate temperatures in dichloromethane was employed, we could get octalin **9** in 42% yield over four steps and the undesired *cis*-fused octalin **24** was given in 6% over four steps, which shows a high selectivity of *trans*-fused octalin. Therefore, we determined that condition B was the best condition for the large-scale synthesis of *trans*-octalin **9**, which gives the most amount of *trans*-octalin **9** without proceeding with the thermal IMDA reaction in the former Wittig reaction. The enantiomeric purity of the octalin skeleton was confirmed to be >99.99% ee at the later step, and it is demonstrated that gram-scale synthesis conditions do not affect the enantiomeric purity of the octalin skeleton.

**Scheme 4:**
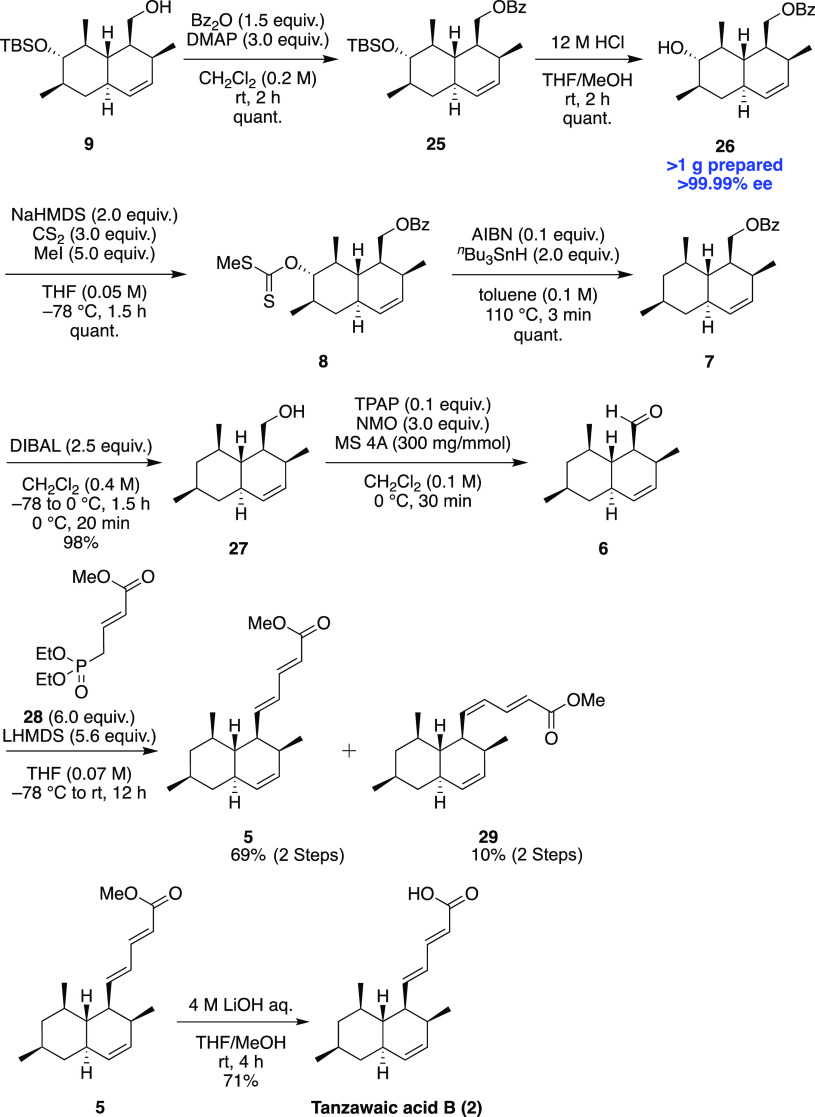
Total Synthesis of Tanzawaic Acid B

After the establishment of the gram-scale synthesis of *trans*-octalin **9**, we pursued the total synthesis of tanzawaic acid B (Scheme 4). The acylation of the hydroxyl group following deprotection of the TBS group gave alcohol **26**. The alcohol was led to xanthate **8**,^10^ and **8** was deoxygenated to benzoate **7** under the conditions of the Barton–McCombie deoxygenation reaction.^11^ After reductive deprotection of the benzoyl group, alcohol **27** was oxidized to **6** and aldehyde **6** was immediately used for the next HWE reaction to afford pentadienoic ester **5**. The hydrolysis of methyl ester **5** to accomplish the total synthesis of tanzawaic acid B was carried out.

Since we could obtain the synthetic sample of tanzawaic acid B, we compared the ^1^H and ^13^C NMR spectrum of the synthetic sample with the ones of the natural tanzawaic acid B (see details in the Supporting Information). The ^1^H and ^13^C NMR spectrum of the synthetic sample was in accordance to the ones of the natural tanzawaic acid B.^3n,12^ In addition, the optical rotation of synthetic **2** matches the one of naturally occurring tanzawaic acid B. Therefore, we demonstrated that the absolute configuration of tanzawaic acid B was (2*E*,4*E*,1*S*,2*S*,4a*R*,6*S*,8*R*,8a*S*)-tanzawaic acid B.

In conclusion, the improvement in the gram-scale synthesis of *trans*-octalin **9** facilitates us to achieve the first total synthesis of (+)-tanzawaic acid B (**2**). The comparison of the ^1^H NMR spectrum, the ^13^C NMR spectrum, and the optical rotation between the synthetic tanzawaic acid B and the reported naturally occurring tanzawaic acid B demonstrated that the chemical structure and stereochemistry of our synthetic tanzawaic acid B match the naturally occurring tanzawaic acid B. The further improvement of synthesis of **2** and investigation of the biological activity of tanzawaic acid B and the synthetic analogues are currently underway.

## Supporting Information Available

^1^H NMR spectra, ^13^C NMR spectra, and the other physical properties of all new compounds and the improvement of the synthetic procedure and the comparison of the NMR spectra between the synthetic sample and natural tanzawaic acid B (PDF)
