# Correction to “First Total Synthesis of Tanzawaic Acid B”

**DOI:** 10.1021/acsomega.3c05579

**Published:** 2023-08-14

**Authors:** Takatsugu Murata, Hisazumi Tsutsui, Takumi Yoshida, Hirokazu Kubota, Shintaro Hiraishi, Hiyo Natsukawa, Yuki Suzuki, Daiki Hiraga, Takahiro Mori, Yutaro Maekawa, Satoru Tateyama, Kiyotaka Toyoyama, Keiichi Ito, Kyohei Suzuki, Keita Yonekura, Natsumi Shibata, Teruyuki Sato, Yasutaka Tasaki, Takehiko Inohana, Atsuhiro Takano, Naoki Egashira, Masaki Honda, Yuma Umezaki, Isamu Shiina

After our paper was published online, we discovered a mistake of structure of “tanzawaic acid D” in Figure 1. Corrected Figure 1 as below (the structure of tanzawaic acid D was replaced to the correct one):

**Figure 1:**
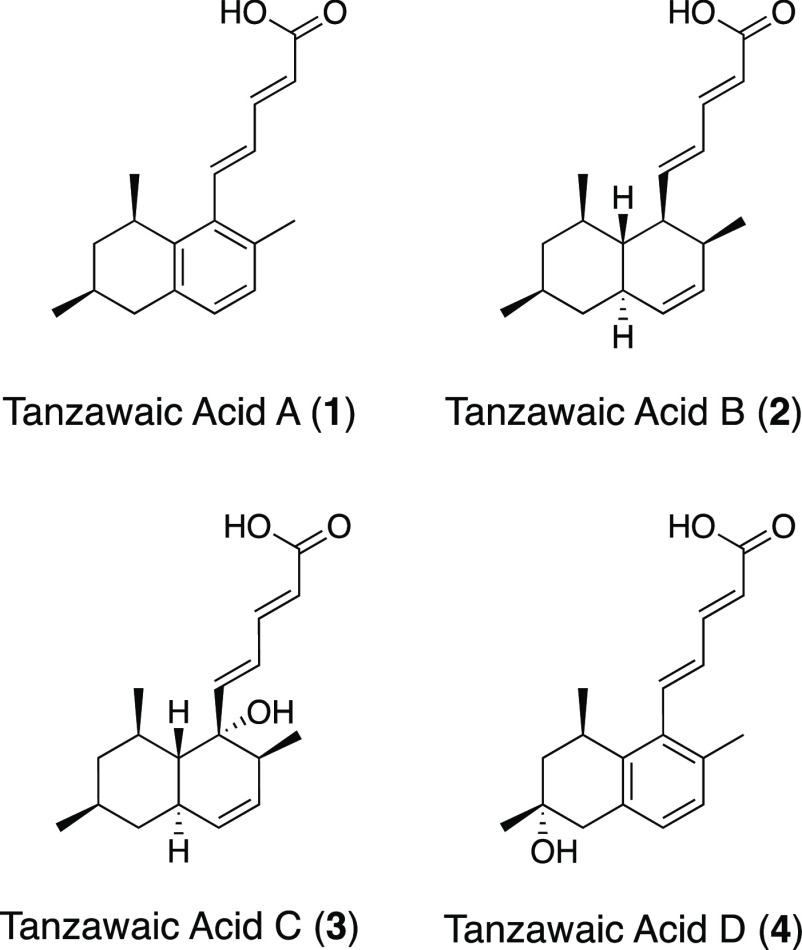
Tanzawaic acids A–D.

