# Supplementary material for: First Total Synthesis of Tanzawaic Acid B
Source: ACS Omega. 2023 Jul 19;8(30):27703–9. doi: 10.1021/acsomega.3c03634 (PMC10399178; doi:10.1021/acsomega.3c03634)
Supplement: Supplementary file 1 — ao3c03634_si_001.pdf [file ao3c03634_si_001.pdf]

The First Total Synthesis of Tanzawaic Acid B

Takatsugu Murata, Hisazumi Tsutsui, Takumi Yoshida, Hirokazu Kubota, Shintaro Hiraishi, Hiyo Natsukawa, Yuki Suzuki, Daiki Hiraga, Takahiro Mori, Yutaro Maekawa, Satoru Tateyama, Kiyotaka Toyoyama, Keiichi Ito, Kyohei Suzuki, Keita Yonekura, Natsumi Shibata, Teruyuki Sato, Yasutaka Tasaki, Takehiko Inohana, Atsuhiro Takano, Naoki Egashira, Masaki Honda, Yuma Umezaki, Isamu Shiina*

†Department of Applied Chemistry, Faculty of Science, Tokyo University of Science, 1-3 Kagurazaka, Shinjuku-ku, Tokyo 162-8601, Japan

Supporting Information

S1	General Information
S1	Reagents
S3	Preparation of Tanzawaic Acid B
S18	Comparison of ^{1}H and ^{13}C NMR Spectra
S19	References
S20	^{1}H and ^{13}C NMR Spectroscopic Data of Compounds

General Information. Melting points are recorded on a Yamano MP-S3. ^{1}H and ^{13}C NMR spectra were recorded on a JEOL JNM-EX300L or JEOL JNM-EX500L and with chloroform (in chloroform-*d*) or with benzene (in benzene-d_6) as internal standard. Infrated spectra (FT-IR) were recorded on a Horiba FT-300. Absorbance frequencies are recorded in reciprocal centimeters (cm^{-1}). High resolution mass spectra (HRMS) were obtained from a Bruker Daltonics micro TOF focus. Optical rotations were determined using a Jasco P-1020. All reactions were carried out under argon atmosphere in dried glassware.

Reagents.

Dichloromethane was purchased from Kokusan Chemical Co., Ltd. and distilled from phosphorus pentoxide, then calcium hydride and dried over Molecular Sieves 4A.

Tetrahydrofuran (Tetrahydrofuran, Dehydrated stabilizer free) was purchased from Kanto Chemical Co., Inc. and used as received.

Dimethyl sulfoxide was purchased from FUJIFILM Wako Pure Chemical Corp. and distilled from calcium hydride and dried over Molecular Sieves 4A.

Methanol was purchased from Kokusan Chemical Co., Ltd. and distilled from Magnesium/Iodine and dried over Molecular Sieves 3A.

Toluene was purchased from Kokusan Chemical Co., Ltd. and distilled from phosphorus pentoxide and dried over Molecular Sieves 4A.

Phosphorus pentoxide was purchased from Kokusan Chemical Co., Ltd. and used as received.
Calcium hydride was purchased from Junsei Chemical Co., Ltd. and used as received.
Magnesium was purchased from Nacalai Tesque, Inc. and used as received.
Iodine was purchased from Kanto Chemical Co., Inc. and used as received.
Potassium hydroxide was purchased from Kokusan Chemical Co., Ltd. and used as received.
Column chromatography was performed on Silica gel 60 (35-70 μm) for column chromatography and the silica gel was purchased from Merck KGaA.
Thin layer chromatography was performed on Wakogel B5F.
(2*E*,4*E*)-Hexa-2,4-dien-1-ol was purchased from Tokyo Kasei Kogyo Co., Ltd. and used as received.
Phosphorus tribromide was purchased from Tokyo Kasei Kogyo Co., Ltd. and used as received.
(*S*)-4-Benzyl-3-propionyloxazolidin-2-one (**11**) was prepared from (*S*)-4-Benzyl-oxazolidin-2-one[1]. (*S*)-4-Benzyl-oxazolidin-2-one was purchased from Tokyo Kasei Kogyo Co., Ltd. or FUJIFILM Wako Pure Chemical Corp. and used as received.
(*R*)-4-Benzyl-3-propionyloxazolidin-2-one (*ent*-**11**) was prepared from (*R*)-4-Benzyl-oxazolidin-2-one[1]. (*R*)-4-Benzyl-oxazolidin-2-one was purchased from Tokyo Kasei Kogyo Co., Ltd. or FUJIFILM Wako Pure Chemical Corp. and used as received.
A 1.00 M solution of sodium bis(trimethylsilyl)amide in tetrahydrofuran was purchased from Sigma-Aldrich Co., LLC and used as received.
Tetrabutylammonium iodide was purchased from Tokyo Kasei Kogyo Co., Ltd. and used as received.
Lithium aluminum hydride was purchased from Yoneyama Yakuhin Kogyo Co., Ltd. and used as received.
Sulfur trioxide-pyridine complex was purchased from Sigma-Aldrich Co., LLC and used as received.
Triethylamine was purchased from Kokusan Chemical Co., Ltd. and distilled over potassium hydroxide.
A 1.00 M solution of dibutylboron trifluoromethanesulfonate in dichloromethane was purchased from Sigma-Aldrich Co., LLC and used as received.
A 1.00 M of trimethylaluminum in hexane was purchased from Kanto Chemical Co., Inc. and used as received.
N,*O*-Dimethylhydroxyamine hydrochlroride was purchased from Tokyo Kasei Kogyo Co., Ltd. and used as received.
tert-Butyldimethylsilyl trifluoromethanesulfonate was prepared from *tert*-butyldimethylchlorosilane and trifluoromethanesulfonic acid and distilled[2].
tert-Butyldimethylchlorosilane was purchased from Kanto Chemical Co., Inc. and used as received.
Trifluoromethanesulfonic acid was purchased from Tokyo Kasei Kogyo Co., Ltd. and used as received.
2,6-Lutidine was purchased from Kokusan Chemical Co., Ltd. and used as received.

A 1.03 M of diisobutylaluminum hydride in hexane was purchased from Kanto Chemical Co., Inc. or Tokyo Kasei Kogyo Co., Ltd. and used as received.
Ethyl (triphenylphosphoranylidene)acetate was purchased from Tokyo Kasei Kogyo Co., Ltd. and used as received.
A 0.870 M of diethylaluminum chloride in hexane was purchased from Tokyo Kasei Kogyo Co., Ltd. and used as received.
A 1.00 M of diethylaluminum chloride in hexane was purchased from Kanto Chemical Co., Inc. and used as received.
Benzoic anhydride was purchased from Tokyo Kasei Kogyo Co., Ltd. and used as received.
4-Dimethyaminopyridine was purchased from Tokyo Kasei Kogyo Co., Ltd. and recrystallized with toluene.
12.0 M aqueous Hydrochloride was purchased from Kokusan Chemical Co., Ltd. and used as received.
Carbon disulfide was purchased from Tokyo Kasei Kogyo Co., Ltd. and used as received.
Iodomethane was purchased from Tokyo Kasei Kogyo Co., Ltd. and used as received.
2,2'-(Diazene-1,2-diyl)bis(2-methylpropanenitrile) was purchased from Tokyo Kasei Kogyo Co., Ltd. and used as received.
Tributyltin hydride was purchased from Kanto Chemical Co., Inc. and used as received.
4-Methylmorpholine *N*-oxide was purchased from Tokyo Kasei Kogyo Co., Ltd. and used as received.
Tetrapropylammonium perruthenate was purchased from Tokyo Kasei Kogyo Co., Ltd. and used as received.
A 1.00 M solution of lithium bis(trimethylsilyl)amide in tetrahydrofuran was purchased from Sigma-Aldrich Co., LLC and used as received.
Phosphate **28** [methyl (*E*)-4-(diethoxyphosphoryl)but-2-enoate] was prepared from methyl 4-bromocrotonate[3]. Methyl 4-bromocrotonate was purchased from Tokyo Kasei Kogyo Co., Ltd. or FUJIFILM Wako Pure Chemical Corp. and used as received.
Lithium hydroxide was purchased from Merck KGaA and used as received.

Preparation of Tanzawaic Acid B.

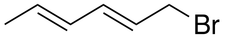

(2*E*,4*E*)-1-Bromohexa-2,4-diene (13)
Dienyl bromide **13** was prepared according to the literature[4] method with modification. Phosphorus tribromide (9.7 mL, 103 mmol) was added to a solution of (2*E*,4*E*)-hexa-2,4-dien-1-ol (10.0 g, 103 mmol) in dichloromethane (51.0 mL) at 0 °C. After the reaction mixture was stirred for 1 h at 0 °C, ice water was added. After the mixture was separated into aqueous layer and organic layer, the organic layer was washed with brine (equal volume to organic layer), and dried over sodium sulfate. After filtration of the mixture and concentration of the solvent, the cooled crude product was filtered through a short pad of silica gel (100 g), which was cooled by utilizing of vaporization's enthalpy of

solvent (hexane/ethyl acetate = 19/1) {eluant (cooled to 0 °C in advance); hexane/ethyl acetate = 10/1}. After concentration of the solvent, crude bromide **13** (14.6 g, 89%) was obtained as brown oil.

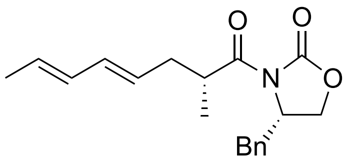

(*S*)-4-Benzyl-3-[(2'*R*,4'*E*,6'*E*)-2'-methylocta-4',6'-dienoyl]oxazolidin-2-one (14)
A solution of oxazolidinone **11** (14.1 g, 60.6 mmol) in tetrahydrofuran (25.0 mL) was added fast to a solution of sodium bis(trimethylsilyl)amide (1.00 M in tetrahydrofuran, 66.6 mL, 66.6 mmol) in tetrahydrofuran (25.6 mL) at –78 °C. After the mixture was stirred for 15 min at –78 °C, a solution of bromide **13** (14.6 g, 90.9 mmol) in tetrahydrofuran (10.0 mL) was added fast to the reaction mixture and tetrabutylammonium iodide (2.24 g, 6.06 mmol) was added to the reaction mixture. After the reaction mixture was stirred for 1 h at room temperature, saturated aqueous ammonium chloride was added at 0 °C. The mixture was extracted with ethyl acetate, the organic layer was washed with brine, and dried over sodium sulfate. After filtration of the mixture and concentration of the solvent, the crude product was purified by column chromatography on silica (eluant; hexane/ethyl acetate = 10/1 to 7/1) to afford oxazolidinone **14** (15.7 g, 83%) as colorless oil.
IR (neat): 1782, 1697 cm^{-1};
$[\alpha]_D^{23}$ +54.0 (*c* 1.04, $CHCl_3$);
^{1}H NMR (500 MHz, $CDCl_3$): δ = 7.38-7.15 (m, 5H, Ph), 6.11-5.92 (m, 2H, H-6', H-5'), 5.61 (dq, *J* = 14.5, 7.0 Hz, 1H, H-7'), 5.55 (ddd, *J* = 14.5, 7.5, 7.0 Hz, 1H, H-4'), 4.71-4.66 (m, 1H, H-4), 4.18 (dd, *J* = 9.0, 9.0 Hz, 1H, Bn), 4.14 (dd, *J* = 9.0, 3.0 Hz, 1H, Bn), 3.85 (ddq, *J* = 7.0, 7.0, 7.0 Hz, 1H, H-2'), 3.26 (dd, *J* = 13.5, 3.0 Hz, 1H, H-5), 2.68 (dd, *J* = 13.5, 10.0 Hz, 1H, H-5), 2.51 (ddd, *J* = 14.0, 7.0, 7.0 Hz, 1H, H-3'), 2.24 (ddd, *J* = 14.0, 7.5, 7.0 Hz, 1H, H-3'), 1.72 (d, *J* = 7.0 Hz, 3H, H-8'), 1.18 (d, *J* = 7.0 Hz, 3H, 2'-Me);
^{13}C NMR (125 MHz, $CDCl_3$): δ = 176.6 (C-1'), 153.1 (C-2), 135.4 (Ph), 133.0 (C-6'), 131.2 (C-5'), 129.3 (Ph), 128.9 (Ph), 128.0 (C-7'), 127.6 (C-4'), 127.2 (Ph), 65.9 (Bn), 55.2 (C-4), 38.0 (C-5), 37.7 (C-2'), 37.0 (C-3'), 18.0 (C-8'), 16.4 (2'-Me);
HRMS: *m*/*z* $[M + Na]^+$ calcd for $C_{19}H_{23}O_3NNa$: 336.1570; found: 336.1576.

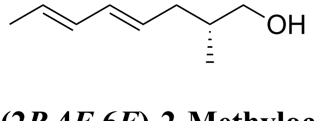

(2*R*,4*E*,6*E*)-2-Methylocta-4,6-dien-1-ol (15)
A solution of oxazolidinone **14** (18.7 g, 59.7 mmol) in tetrahydrofuran (66.0 mL) was added to a suspension of lithium aluminum hydride (3.40 g, 89.6 mmol) in tetrahydrofuran (133 mL) at 0 °C. After the reaction mixture was stirred for 1 h at room temperature, methanol and 1.0 M aqueous hydrochloride were added at 0 °C. The mixture was extracted with ethyl acetate, the organic layer was washed with brine, and dried over sodium sulfate. After filtration of the mixture and concentration of the solvent, the crude product was

purified by column chromatography on silica (eluant; hexane/ethyl acetate = 5/1) to afford alcohol **15** (7.19 g, 86%) as colorless oil.
IR (neat): 3340 cm^{-1};
$[\alpha]_D^{24}$ +5.72 (*c* 1.00, $CHCl_3$);
^1H NMR (300 MHz, $CDCl_3$): δ = 6.10-5.96 (m, 2H, H-6, H-5), 5.68-5.47 (m, 2H, H-7, H-4), 3.51 (ddd, *J* = 10.7, 5.7, 4.8 Hz, 1H, H-1), 3.46 (ddd, *J* = 10.8, 6.0, 4.8 Hz, 1H, H-1), 2.17 (ddd, *J* = 13.5, 6.9, 6.6 Hz, 1H, H-3), 1.95 (ddd, *J* = 13.5, 7.2, 6.9 Hz, 1H, H-3), 1.77-1.55 (m, 1H, H-2), 1.74 (d, *J* = 5.7 Hz, 3H, H-8), 1.30 (br dd, *J* = 6.0, 5.7 Hz, 1H, OH), 0.92 (d, *J* = 6.9 Hz, 3H, 2-Me);
^{13}C NMR (75 MHz, $CDCl_3$): δ = 131.9 (C-6), 131.4 (C-5), 129.5 (C-7), 127.2 (C-4), 67.8 (C-1), 36.4 (C-2), 36.0 (C-3), 17.9 (C-8), 16.4 (2-Me);
HRMS: *m/z* $[M]^+$ calcd for $C_9H_{16}O$: 140.1201; found: 140.1202.

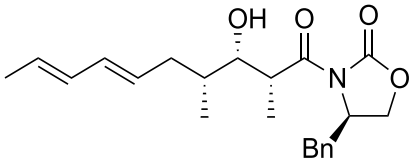

(*R*)-4-Benzyl-3-[(2'*R*,3'*S*,4'*R*,6'*E*,8'*E*)-3'-hydroxy-2',4'-dimethyldeca-6',8'-dienoyl]oxazolidin-2-one (17)
[preparation of aldehyde **16**]
Sulfur trioxide-pyridine complex (47.5 g, 299 mmol) was added to a solution of alcohol **15** (10.5 g, 74.9 mmol), triethylamine (83.0 mL, 599 mmol), and dimethyl sulfoxide (62.4 mL, 879 mmol) in dichloromethane (250 mL) at 0 °C. After the reaction mixture was stirred for 1 h at room temperature, aldehyde **16** was obtained and used in the next step without further purification.
[preparation of the boron enolate]
A 1.00 M solution of dibutylboron trifluoromethanesulfonate in dichloromethane (74.9 mL, 74.9 mmol) and triethylamine (11.4 mL, 82.4 mmol) were added to a solution of oxazolidinone *ent*-**11** (17.4 g, 74.9 mmol) in dichloromethane (250 mL) at 0 °C. After the mixture was stirred for 10 min at 0 °C, the mixture of boron enolate was used in the following reaction without further purification.
[preparation of **17**]
The reaction mixture of aldehyde **16** was added via a cannula to a solution of the boron enolate at –78 °C. After the reaction mixture was stirred for 30 min at –78 °C and for 1 h at 0 °C, methanol (40 mL), phosphate buffer solution (pH 7) (240 mL), and 35% aqueous hydrogen peroxide (40 mL) were added at 0 °C. After the reaction mixture was stirred for 30 min at 0 °C, saturated aqueous sodium thiosulfate (200 mL) was added. The mixture was extracted with dichloromethane, the organic layer was washed with 1.0 M aqueous hydrochloride and brine, and dried over sodium sulfate. After filtration of the mixture and concentration of the solvent, the crude product was purified by column chromatography on silica (eluant; hexane/ethyl acetate = 7/1 to 3/1) to afford crude aldol **17** (32.6 g) as pale yellow oil. Then, crude aldol **17** was purified by column chromatography on silica (eluant;

hexane/ethyl acetate = 10/1 to 4/1) to afford aldol **17** (19.5 g, 70%) as colorless oil.
IR (neat): 3471, 1782 cm^{-1};
$[\alpha]_D^{24}$ –39.9 (*c* 1.01, $CHCl_3$);
^1H NMR (500 MHz, $CDCl_3$): δ = 7.40-7.10 (m, 5H, Ph), 6.10-5.90 (m, 2H, H-8', H-7'), 5.60 (dq, *J* = 14.0, 7.0 Hz, 1H, H-9'), 5.51 (ddd, *J* = 14.5, 7.0, 7.0 Hz, 1H, H-6'), 4.73-4.60 (m, 1H, H-4), 4.21 (dd, *J* = 12.5, 4.0 Hz, 1H, Bn), 4.19 (dd, *J* = 12.5, 2.5 Hz, 1H, Bn), 3.99 (qd, *J* = 6.0, 3.5 Hz, 1H, H-2'), 3.71 (ddd, *J* = 7.5, 3.5, 3.5 Hz, 1H, H-3'), 3.25 (dd, *J* = 13.5, 2.5 Hz, 1H, H-5), 2.78 (dd, *J* = 13.5, 8.5 Hz, 1H, H-5), 2.59 (d, *J* = 3.5 Hz, 1H, OH), 2.20 (ddd, *J* = 13.0, 7.0, 6.0 Hz, 1H, H-5'), 1.94 (ddd, *J* = 13.0, 8.5, 7.0 Hz, 1H, H-5'), 1.80-1.63 (m, 1H, H-4'), 1.73 (d, *J* = 7.0 Hz, 3H, H-10'), 1.28 (d, *J* = 6.0 Hz, 3H, 2'-Me), 0.98 (d, *J* = 7.5 Hz, 3H, 4'-Me);
^{13}C NMR (125 MHz, $CDCl_3$): δ = 177.3 (C-1'), 152.8 (C-2), 135.0 (Ph), 132.2 (C-7'), 131.5 (C-8'), 129.4 (Ph), 129.0 (C-6'), 128.9 (Ph), 127.4 (Ph), 127.3 (C-9'), 74.7 (C-3'), 66.1 (Bn), 55.1 (C-4), 39.9 (C-2'), 37.7 (C-5), 36.5 (C-5'), 35.9 (C-4'), 17.9 (C-10'), 15.0 (2'-Me), 11.5 (4'-Me);
HRMS: *m*/*z* $[M + Na]^+$ calcd for $C_{22}H_{29}O_4NNa$: 394.1989; found: 394.1981.

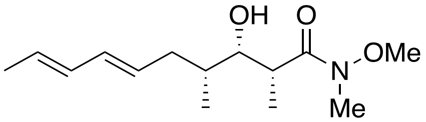

(2*R*,3*S*,4*R*,6*E*,8*E*)-3-Hydroxy-*N*-methoxy-*N*,2,4-trimethyldeca-6,8-dienamide (18)
A 1.06 M solution of trimethylaluminum in hexane (172 mL, 182 mmol) was added to a solution of *N*,*O*-dimethylhydroxylamine hydrochloride (17.8 g, 182 mmol) in tetrahydrofuran (290 mL) at 0 °C. After the mixture was stirred for 15 min at 0 °C and for 15 min at room temperature, a solution of aldol **17** (26.0 g, 72.6 mmol) in tetrahydrofuran (70.0 mL) was added at 0 °C. After the mixture was stirred for 10 min at 0 °C, 1.0 M aqueous hydrochloride was added at 0 °C. The acidified mixture (pH 4) was extracted with dichloromethane, the organic layer was washed with brine, and dried over sodium sulfate. After filtration of the mixture and concentration of the solvent, the crude product was purified by column chromatography on silica (eluant; hexane/ethyl acetate = 3/1 to 2/1) to afford amide **18** (14.1 g, 79%) as colorless oil, and the mixture of product and impurity (4.04 g). Then, the mixture of product and impurity was purified by column chromatography on silica (eluant; hexane/ethyl acetate = 4/1 to 3/1) to afford amide **18** (2.65 g, 15%) as colorless oil and the mixture of product and impurity (804 mg).
Isolated Yield: 16.8 g, 94%;
IR (neat): 3433, 1643 cm^{-1};
$[\alpha]_D^{27}$ +1.9 (*c* 1.28, $CHCl_3$);
^1H NMR (500 MHz, $CDCl_3$): δ = 6.10-5.92 (m, 2H, H-8, H-7), 5.75.-5.45 (m, 2H, H-9, H-6), 3.69 (s, 3H, OMe), 3.59 (dd, *J* = 7.5, 4.0 Hz, 1H, H-3), 3.19 (s, 3H, NMe), 3.19-3.05 (m, 1H, H-2), 2.17 (ddd, *J* = 14.5, 5.5, 5.5 Hz, 1H, H-5), 1.95 (ddd, *J* = 14.5, 8.0, 7.0 Hz, 1H, H-5), 1.80-1.65 (m, 1H, H-4), 1.73 (d, *J* = 7.0 Hz, 3H, H-10), 1.17 (d, *J* = 7.5 Hz, 3H, 2-Me), 1.00 (d, *J* = 7.0 Hz, 3H, 4-Me):

^{13}C NMR (125 MHz, $CDCl_3$): δ = 178.0 (C-1), 132.1 (C-8), 131.4 (C-7), 129.1 (C-9), 127.2 (C-6), 75.1 (C-3), 61.5 (OMe), 36.6 (C-4), 36.2 (C-2), 35.4 (C-5), 31.9 (NMe), 17.9 (C-10), 15.2 (2-Me), 11.0 (4-Me);
HRMS: *m*/*z* $[M + Na]^+$ calcd for $C_{14}H_{25}O_3NNa$: 278.1727; found: 278.1735.

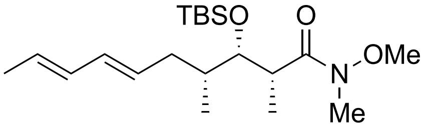

(2*R*,3*S*,4*R*,6*E*,8*E*)-3-(*tert*-Butyldimethylsiloxy)-*N*-methoxy-*N*,2,4-trimethyldeca-6,8-dienamide (19)

To a solution of amide **18** (16.8 g, 65.7 mmol) in dichloromethane (219 mL) at 0 °C , 2,6-lutidine (22.7 mL, 197 mmol) and *tert*-butyldimethylsilyl trifluoromethanesulfonate (30.0 mL, 131 mmol) were added. After the reaction mixture was stirred for 15 min at 0 °C, saturated aqueous ammonium chloride was added. The mixture was extracted with dichloromethane, the organic layer was washed with 1.0 M aqueous hydrochloride and brine, and dried over sodium sulfate. After filtration of the mixture and concentration of the solvent, the crude product was purified by column chromatography on silica (eluant; hexane/ethyl acetate = 20/1 to 4/1) to afford TBS ether **19** (24.5 g, quant.) as colorless oil.
IR (neat): 1666 cm^{-1};
$[\alpha]_D^{25}$ +5.6 (*c* 0.720, $CHCl_3$);
^{1}H NMR (500 MHz, $CDCl_3$): δ = 6.08-5.90 (m, 2H, H-8, H-7), 5.63-5.51 (m, 1H, H-9), 5.48 (ddd, *J* = 14.5, 7.5, 7.5 Hz, 1H, H-6), 3.93-3.83 (m, 1H, H-3), 3.69 (s, 3H, OMe), 3.16 (s, 3H, NMe), 3.30-2.95 (m, 1H, H-2), 2.33-2.15 (m, 1H, H-5), 2.00-1.80 (m, 1H, H-5), 1.72 (d, *J* = 7.5 Hz, 3H, H-10), 1.63-1.44 (m, 1H, H-4,), 1.14 (d, *J* = 7.0 Hz, 3H, 2-Me), 0.92 (s, 9H, TBS), 0.81 (d, *J* = 6.0 Hz, 3H, 4-Me), 0.10 (s, 3H, TBS), 0.08 (s, 3H, TBS);
^{13}C NMR (125 MHz, $CDCl_3$): δ = 177.0 (C-1), 131.7 (C-8), 131.5 (C-7), 130.8 (C-9), 126.7 (C-6), 77.0 (C-3), 61.4 (OMe), 38.9 (C-2), 38.6 (C-4), 37.2 (C-5), 32.2 (NMe), 26.2 (TBS), 18.4 (TBS), 18.0 (C-10), 15.8 (2-Me), 13.4 (4-Me), –3.69 (TBS), –3.72 (TBS);
HRMS: *m*/*z* $[M + Na]^+$ calcd for $C_{20}H_{39}O_3NNa$: 392.2591; found: 392.2607.

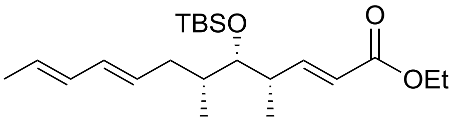

Ethyl (2*E*,4*S*,5*S*,6*R*,8*E*,10*E*)-5-(*tert*-butyldimethylsiloxy)-4,6-dimethyldodeca-2,8,10-trienoate (10)

To a solution of TBS ether **19** (11.3 g, 30.6 mmol) in tetrahydrofuran (204 mL) at –78 °C, a 1.03 M solution of diisobutylaluminum hydride in hexane (38.6 mL, 39.8 mmol) was added. After the reaction mixture was stirred for 1 h at 0 °C, methanol and saturated aqueous Rochelle salt were added. The mixture was extracted with ethyl acetate, the organic layer was washed with brine, and dried over sodium sulfate. After filtration of the mixture and

concentration of the solvent, the residue was dried under reduced pressure to afford aldehyde **20** (9.80 g). The crude aldehyde **20** was used in the following reaction without further purification.

Ethyl (triphenylphosphoranylidene)acetate **12** (21.3 g, 61.2 mmol) was added to a solution of aldehyde **20** in dichloromethane (102 mL) at room temperature. The mixture was stirred for 18 h at 35 °C. After concentration of the solvent, the crude product was filtered through a short pad of silica (eluant; hexane/ethyl acetate = 15/1). After concentration of the solvent, crude ester **10** (11.2 g) was obtained as pale yellow oil. The crude ester **10** was purified by column chromatography on silica (eluant; hexane/ethyl acetate = 20/1) to afford ester **10** (10.1 g, 87%) as colorless oil.

IR (neat): 1720 cm^{-1};

$[\alpha]_D^{25}$ –23.0 (*c* 1.01, $CHCl_3$);

^1H NMR (500 MHz, $CDCl_3$): δ = 6.99 (dd, *J* = 15.5, 8.5 Hz, 1H, H-3), 6.07-5.91 (m, 2H, H-10, H-9), 5.77 (d, *J* = 15.5 Hz, 1H, H-2), 5.58 (dq, *J* = 14.0, 7.0 Hz, 1H, H-11), 5.46 (ddd, *J* = 14.5, 7.5, 7.0 Hz, 1H, H-8), 4.19 (q, *J* = 7.0 Hz, 2H, OEt), 3.51 (dd, *J* = 6.5, 4.0 Hz, 1H, H-5), 2.62-2.40 (m, 1H, H-4), 2.14 (ddd, *J* = 13.5, 7.0, 6.5 Hz, 1H, H-7), 1.90 (ddd, *J* = 13.5, 7.5, 6.0 Hz, 1H, H-7), 1.73 (d, *J* = 7.0 Hz, 3H, H-12), 1.68-1.54 (m, 1H, H-6), 1.29 (t, *J* = 7.0 Hz, 3H, OEt), 1.04 (d, *J* = 7.5 Hz, 3H, 4-Me), 0.91 (s, 9H, TBS), 0.82 (d, *J* = 7.5 Hz, 3H, 6-Me), 0.06 (s, 3H, TBS), 0.05 (s, 3H, TBS);

^{13}C NMR (125 MHz, $CDCl_3$): δ = 166.7 (C-1), 152.4 (C-3), 131.8 (C-9), 131.6 (C-10), 130.3 (C-8), 127.1 (C-11), 120.2 (C-2), 78.4 (C-5), 60.1 (OEt), 41.2 (C-4), 37.7 (C-7), 37.4 (C-6), 26.1 (TBS), 18.4 (TBS), 18.0 (C-12), 15.6 (4-Me), 14.2 (6-Me), 14.2 (OEt), –3.78 (TBS), –3.80 (TBS);

HRMS: *m*/*z* $[M + Na]^+$ calcd for $C_{22}H_{40}O_3SiNa$: 403.2639; found: 403.2651.

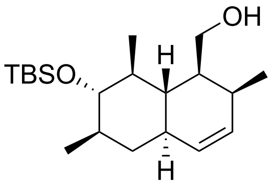

[(1'*S*,2'*S*,4'a*R*,6'*R*,7'*S*,8'*S*,8'a*S*)-7'-(*tert*-Butyldimethylsilyloxy)-2',6',8'-trimethyl-1',2',4'a,5',6',7',8',8'a-octahydronaphthalen-1'-yl]methanol (9)

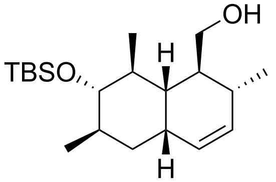

[(1'*S*,2'*R*,4'a*S*,6'*R*,7'*S*,8'*S*,8'a*S*)-7'-(*tert*-Butyldimethylsilyloxy)-2',6',8'-trimethyl-1',2',4'a,5',6',7',8',8'a-octahydronaphthalen-1'-yl]methanol (24)

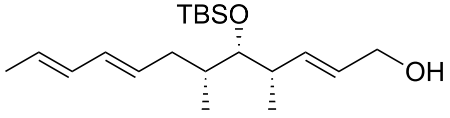

(2*E*,4*S*,5*S*,6*R*,8*E*,10*E*)-5-(*tert*-butyldimethylsilyloxy)-4,6-dimethyldodeca-2,8,10-trien-1

-ol (S1)

A 0.870 M solution of diethylaluminum chloride in hexane (120 mL, 105 mmol) was added to a solution of ester **10** (10.1 g, 26.2 mmol) in dichloromethane (262 mL) at –78 °C. After the mixture was stirred for 16 h at room temperature, 1.0 M aqueous hydrochloride was added at 0 °C. The acidified mixture (pH = 4) was extracted with dichloromethane, and the organic layer was dried over sodium sulfate. After filtration of the mixture and concentration of the solvent, the residue was dried under reduced pressure to afford crude reaction mixture (**10**, **21**, and **22**) (9.83 g), which was used in the following reaction without further purification.

A 1.03 M solution of diisobutylaluminum hydride in hexane (76.3 mL, 78.6 mmol) was added to a solution of the crude reaction mixture (**10**, **21**, and **22**) in dichloromethane (131 mL) at 0 °C. After the mixture was stirred for 10 min at 0 °C, methanol and saturated aqueous Rochelle salt were added. The mixture was extracted with dichloromethane, the organic layer was washed with water and brine, and dried over sodium sulfate. After filtration of the mixture and concentration of the solvent, the crude product was purified by column chromatography on silica (eluant; hexane/ethyl acetate = 20/1) to afford *trans*-fused alcohol **9** (4.27 g, 48%, 2 steps) as colorless oil, *cis*-fused alcohol **24** (658 mg, 7.4%, 2 steps) as colorless oil and reduced alcohol **S1** from ester **10** (2.42 g, 27%) as colorless oil.

[(1'*S*,2'*S*,4'a*R*,6'*R*,7'*S*,8'*S*,8'a*S*)-7'-(*tert*-Butyldimethylsilyloxy)-2',6',8'-trimethyl-1',2',4'a,5',6',7',8',8'a-octahydronaphthalen-1'-yl]methanol (9)

IR (neat): 3348 cm^{-1};

$[\alpha]_D^{23}$ –29.8 (*c* 1.03, $CHCl_3$);

^1H NMR (500 MHz, $CDCl_3$): δ = 5.72 (ddd, *J* = 9.0, 3.5, 3.0 Hz, 1H, H-3’), 5.60 (ddd, *J* = 9.0, 2.5, 2.5 Hz, 1H, H-4’), 3.86-3.72 (m, 1H, H-1), 3.60-3.44 (m, 1H, H-1), 2.88 (dd, *J* = 9.5, 9.5 Hz, 1H, H-7’), 2.43-2.32 (m, 1H, H-2’), 1.88-1.74 (m, 3H, H-1’, H-4’a, H-5’), 1.59-1.49 (m, 1H, H-6’), 1.49-1.38 (m, 1H, H-8’), 1.12 (d, *J* = 7.5 Hz, 3H, 2’-Me), 1.09-1.00 (m, 1H, H-5’), 1.02 (d, *J* = 6.5 Hz, 3H, 8’-Me), 0.98 (d, *J* = 6.5 Hz, 3H, 6’-Me), 0.92 (s, 9H, TBS), 0.92-0.84 (m, 1H, H-8’a) 0.08 (s, 6H, TBS);

^{13}C NMR (125 MHz, $CDCl_3$): δ = 134.6 (C-3’), 134.3 (C-4’), 84.0 (C-7’), 64.8 (C-1), 46.5 (C-8’a), 46.0 (C-1’), 44.3 (C-8’), 40.5 (C-4’a or C-5’), 39.6 (C-6’), 39.0 (C-5’ or C-4’a), 31.7 (C-2’), 26.3 (TBS), 20.3 (6’-Me), 18.5 (TBS), 17.6 (8’-Me), 16.2 (2’-Me), –2.6 (TBS), –2.8 (TBS);

HRMS: *m/z* $[M + Na]^+$ calcd for $C_{20}H_{38}O_2SiNa$: 361.2533; found: 361.2520.

[(1'*S*,2'*R*,4'a*S*,6'*R*,7'*S*,8'*S*,8'a*S*)-7'-(*tert*-Butyldimethylsilyloxy)-2',6',8'-trimethyl-1',2',4'a,5',6',7',8',8'a-octahydronaphthalen-1'-yl]methanol (24)

IR (neat): 3309 cm^{-1};

$[\alpha]_D^{25}$ –22.2 (*c* 1.01, $CHCl_3$);

^1H NMR (500 MHz, $CDCl_3$): δ = 5.54 (ddd, *J* = 10.0, 3.0, 3.0 Hz, 1H, H-3’), 5.38 (br ddd, *J* = 10.0, 3.0, 2.0 Hz, 1H, H-4’), 3.56 (dd, *J* = 10.0, 7.0 Hz, 1H, H-1), 3.52 (dd, *J* = 10.5, 7.0 Hz, 1H, H-1), 2.83 (dd, *J* = 9.0, 9.0 Hz, 1H, H-7’), 2.28-2.20 (m, 1H, H-4’a), 2.03-1.92 (m, 2H, H-2’, H-1’), 1.75-1.60 (m, 2H, H-6’, H-5’), 1.58-1.45 (m, 2H, H-8’, H-8’a), 1.36 (br s, 1H, OH), 1.33-1.22 (m, 1H, H-5’), 1.11 (d, *J* = 7.0 Hz, 3H, 2’-Me), 1.00 (d, *J* = 6.5 Hz, 3H, 6’-Me), 0.92 (d, *J* = 7.0 Hz, 3H, 8’-Me), 0.90 (s, 9H, TBS), 0.06 (s, 3H, TBS), 0.05

(s, 3H, TBS);
^{13}C NMR (125 MHz, $CDCl_3$): δ = 131.7 (C-3’), 129.7 (C-4’), 83.0 (C-7’), 66.6 (C-1), 42.2 (C-1’), 41.0 (C-8’ or C-8’a), 38.1 (C-5’), 37.8 (C-6’), 35.1 (C-8’a or C-8’), 30.9 (C-2’), 30.1 (C-4’a), 26.2 (TBS), 21.6 (2’-Me), 20.1 (8’-Me), 18.5 (TBS), 16.4 (6’-Me), –2.9 (TBS), –3.0 (TBS);
HRMS: *m*/*z* $[M + Na]^+$ calcd for $C_{20}H_{38}O_2SiNa$: 361.2533; found: 361.2529.

Another method
(19) → (9) + (24) + (S1)
A 1.00 M solution of diisobutylaluminum hydride in hexane (74.1 mL, 74.1 mmol) was added to a solution of TBS ether **19** (11.0 g, 29.6 mmol) in tetrahydrofuran (198 mL) at –78 °C. After the reaction mixture was stirred for 15 min at –78 °C, methanol and saturated aqueous Rochelle salt were added. The mixture was extracted with diethyl ether, the organic layer was washed with brine, and dried over sodium sulfate. After filtration of the mixture and concentration of the solvent, the residue was dried under reduced pressure to afford aldehyde **20**. The crude aldehyde **20** was used in the following reaction without further purification.
Ethyl (triphenylphosphoranylidene)acetate **12** (20.7 g, 59.3 mmol) was added to a solution of aldehyde **20** in toluene (98.8 mL) at room temperature. The mixture was stirred for 1 h at 120 °C. After concentration of the solvent, the crude product was purified by column chromatography on silica (eluant; hexane/ethyl acetate = 20/1) to afford crude ester (10.1 g). The crude ester products included ester **10**, *trans*-fused octalin **21**, and *cis*-fused octalin **22** [ester **10**/*trans*-fused octalin **21**/*cis*-fused octalin **22** = 1.00/0.11/0.14 (calculated from ^{1}H NMR spectra)]. The crude ester products were separated to Lot 1,2 and each lot was subjected to the next reaction without further purification.
Lot 1
A 1.00 M solution of diethylaluminum chloride in hexane (54.9 mL, 54.9 mmol) was added to a solution of the ester products (5.22 g, 13.7 mmol) in dichloromethane (275 mL) at –78 °C. After the reaction mixture was stirred for 13 h at room temperature, 1.0 M aqueous hydrochloride was added at 0 °C. The acidified mixture (pH = 4) was extracted with dichloromethane, and the organic layer was dried over sodium sulfate. After filtration of the mixture and concentration of the solvent, the residue was dried under reduced pressure to afford crude reaction mixture (**10**, **21**, and **22**), which was used in the following reaction without further purification.
A 1.00 M solution of diisobutylaluminum hydride in hexane (41.2 mL, 41.2 mmol) was added to a solution of the crude reaction mixture (**10**, **21**, and **22**) in dichloromethane (68.6 mL) at 0 °C. After the mixture was stirred for 20 min at 0 °C, methanol and saturated aqueous Rochelle salt were added. The mixture was extracted with dichloromethane, the organic layer was washed with water and brine, and dried over sodium sulfate. After filtration of the mixture and concentration of the solvent, the crude product was purified by column chromatography on silica (eluant; hexane/ethyl acetate = 30/1) to afford *trans*-fused alcohol **9** (1.57 g, 16%, 4 steps) as colorless oil, *cis*-fused alcohol **24** (1.01 g, 10%, 4 steps)

as colorless oil and reduced alcohol **S1** from ester **10** (677 mg, 6.7%, 4 steps) as colorless oil.

Lot 2

A 1.00 M solution of diethylaluminum chloride in hexane (51.4 mL, 51.4 mmol) was added to a solution of the ester products (4.89 g, 12.9 mmol) in dichloromethane (257 mL) at –78 °C. After the reaction mixture was stirred for 19 h at room temperature, 1.0 M aqueous hydrochloride was added at 0 °C. The acidified mixture (pH = 4) was extracted with dichloromethane, and the organic layer was dried over sodium sulfate. After filtration of the mixture and concentration of the solvent, the residue was dried under reduced pressure to afford crude reaction mixture (**10**, **21**, and **22**), which was used in the following reaction without further purification.

A 1.00 M solution of diisobutylaluminum hydride in hexane (38.9 mL, 38.9 mmol) was added to a solution of the crude reaction mixture (**10**, **21**, and **22**) in dichloromethane (64.3 mL) at 0 °C. After the mixture was stirred for 45 min at 0 °C, methanol and saturated aqueous Rochelle salt were added. The mixture was extracted with dichloromethane, the organic layer was washed with water and brine, and dried over sodium sulfate. After filtration of the mixture and concentration of the solvent, the crude product was purified by column chromatography on silica (eluant; hexane/ethyl acetate = 30/1) to afford *trans*-fused alcohol **9** (1.43 g, 14%, 4 steps) as colorless oil, *cis*-fused alcohol **24** (670 mg, 7%, 4 steps) as colorless oil and reduced alcohol **S1** from ester **10** (538 mg, 5.4%, 4 steps) as colorless oil.

Isolated Yield (Lot 1 + Lot 2): 2.99 g, 30% (*trans*-fused alcohol **9**), 1.71 g, 17% (*cis*-fused alcohol **24**), 1.21 g, 12% (reduced alcohol **S1** from ester **10**).

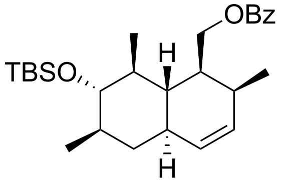

[(1'*S*,2'*S*,4'a*R*,6'*R*,7'*S*,8'*S*,8'a*S*)-7'-(*tert*-Butyldimethylsilyloxy)-2',6',8'-trimethyl-1',2',4'a,5',6',7',8',8'a-octahydronaphthalen-1'-yl]methyl benzoate (25)

Benzoic anhydride (641 mg, 2.84 mmol) was added to a solution of alcohol **9** (640 mg, 1.89 mmol) and 4-dimethylaminopyridine (693 mg, 5.67 mmol) in dichloromethane (9.5 mL) at 0 °C. Then, the reaction mixture was stirred for 2 h at room temperature. After the concentration of the solvent, the crude product was purified by column chromatography on silica (eluant; hexane/ethyl acetate = 20/1) to afford benzoate **25** (836 mg, quant.) as white solid.

Mp: 95.7 °C;

IR (KBr): 1720, 1458, 1088 cm^{-1};

$[\alpha]_D^{18}$ +1.69 (*c* 1.07, $CHCl_3$);

^{1}H NMR (500 MHz, $CDCl_3$): δ = 8.08-8.00 (m, 2H, Bz), 7.59-7.51 (m, 1H, Bz), 7.48-7.40 (m, 2H, Bz), 5.67 (ddd, *J* = 9.0, 3.5, 3.5 Hz, 1H, H-3'), 5.57 (ddd, *J* = 9.0, 2.5, 2.5 Hz, 1H, H-4'), 4.36 (dd, *J* = 11.5, 4.5 Hz, 1H, H-1), 4.23 (dd, *J* = 11.5, 7.5 Hz, 1H, H-1), 2.88 (dd, *J*

= 9.0, 9.0 Hz, 1H, H-7’), 2.52-2.38 (m, 1H, H-2’), 2.22-2.07 (m, 1H, H-1’), 1.91-1.81 (m, 1H, H-4’a), 1,79 (ddd, *J* = 13.0, 3.5, 3.5 Hz, 1H, H-5’), 1.61-1.44 (m, 2H, H-6’, H-8’), 1.104 (d, *J* = 7.0 Hz, 3H, 2’-Me), 1,101 (d, *J* = 6.5 Hz, 3H, 8’-Me), 1.02-0.96 (m, 1H, H-5’), 0.98 (d, *J* = 6.5 Hz, 3H, 6’-Me), 0.91 (s, 9H, TBS), 0.91-0.85 (m, 1H, H-8’a), 0.07 (s, 6H, TBS);

^{1}H NMR (500 MHz, C_6D_6): δ = 8.25-8.19 (m, 2H, Bz), 7.15-7.06 (m, 3H, Bz), 5.63 (ddd, *J* = 9.0, 3.5, 3.5 Hz, 1H, H-3’), 5.53 (ddd, *J* = 9.0, 2.5, 2.5 Hz, 1H, H-4’), 4.35 (dd, *J* = 11.0, 5.5 Hz, 1H, H-1), 4.30 (dd, *J* = 11.0, 7.0 Hz, 1H, H-1), 2.69 (dd, *J* = 9.5, 9.5 Hz, 1H, H-7’), 2.36-2.23 (m, 1H, H-2’), 2.03-1.89 (m, 1H, H-1’), 1.71-1.59 (m, 1H, H-4’a), 1.53 (ddd, *J* = 13.0, 3.5, 3.5 Hz, 1H, H-5’), 1.51-1.32 (m, 2H, H-6’, H-8’), 1.050 (s, 9H, TBS), 1.048 (d, *J* = 6.0 Hz, 3H, 2’-Me), 1.00 (d, *J* = 7.5 Hz, 3H, 8’-Me), 0.96 (d, *J*= 6.0 Hz, 3H, 6’-Me), 0.77 (ddd, *J* = 13.0, 12.5, 12.5 Hz, 1H, H-5’), 0.72 (ddd, *J* = 10.5, 10.5, 7.0 Hz, 1H, H-8’a), 0.11 (s, 3H, TBS), 0.10 (s, 3H, TBS);

^{13}C NMR (125 MHz, $CDCl_3$): δ = 166.7 (Bz), 133.9 (C-3’), 132.8 (C-4’, Bz), 130.4 (Bz), 129.6 (Bz), 128.3 (Bz), 84.1 (C-7’), 66.9 (C-1), 46.4 (C-8’a), 43.7 (C-8’), 43.3 (C-1’), 40.9 (C-4’a), 39.5 (C-6’), 39.0 (C-5’), 32.3 (C-2’), 26.3 (TBS), 20.3 (6’-Me), 18.5 (TBS), 18.3 (8’-Me), 16.3 (2’-Me), –2.6 (TBS), –2.8 (TBS);

^{13}C NMR (125 MHz, C_6D_6): δ = 166.3 (Bz), 134.2 (C-3’), 133.1 (Bz), 132.8 (C-4’), 131.2 (Bz), 129.9 (Bz), 128.6 (Bz), 84.2 (C-7’), 67.0 (C-1), 46.6 (C-8’a), 44.1 (C-8’), 43.6 (C-1’), 41.1 (C-4’a), 39.8 (C-6’), 39.1 (C-5’), 32.7 (C-2’), 26.5 (TBS), 20.6 (6’-Me), 18.8 (TBS), 18.4 (8’-Me), 16.4 (2’-Me), –2.4 (TBS), –2.6 (TBS)

HRMS: *m*/*z* $[M + Na]^+$ calcd for $C_{27}H_{42}O_3SiNa$: 465.2795; found: 465.2799.

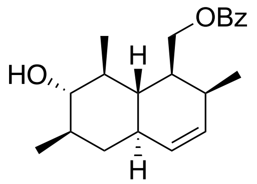

[(1'*S*,2'*S*,4'a*R*,6'*R*,7'*S*,8'*S*,8'a*S*)-7'-Hydroxy-2',6',8'-trimethyl-1',2',4'a,5',6',7',8',8'a-octahydronaphthalen-1'-yl]methyl benzoate (26)

12 M aqueous hydrochloride (3.0 mL, 36.0 mmol) was added to a solution of the benzoate **25** (1.44 g, 3,25 mmol) in methanol (15 mL) and tetrahydrofuran (15 mL) at 0 °C. After the reaction mixture was stirred for 2 h at room temperature, saturated aqueous sodium hydrogen carbonate was added at 0 °C. The mixture was extracted with ethyl acetate, and the organic layer was dried over sodium sulfate. After filtration of the mixture and concentration of the solvent, the crude product was purified by column chromatography on silica (eluant; hexane/ethyl acetate = 4/1 to 2/1) to afford alcohol **26** (1.10 g, quant., >99.99% ee) as white solid.

HPLC (CHIRALPAK® IA-3, hexane/2-propanol = 9/1, flow rate = 0.5 mL/min): t_R = 14.7 min (>99.99%), t_R = 18.0 min (<0.001 %).

Mp: 93.9 °C;

IR (KBr): 3440, 1720, 1458 cm^{-1};

$[\alpha]_D^{20}$ +10.6 (*c* 0.800, $CHCl_3$);

^{1}H NMR (500 MHz, C_6D_6): δ = 8.24-8.18 (m, 2H, Bz), 7.15-7.04 (m, 3H, Bz), 5.60 (ddd, *J* = 9.0, 3.5, 3.5 Hz, 1H, H-3'), 5.46 (ddd, *J* = 9.0, 2.5, 2.5 Hz, 1H, H-4'), 4.42 (dd, *J* = 11.0, 5.0 Hz, 1H, H-1), 4.28 (dd, *J* = 11.0, 8.5 Hz, 1H, H-1), 2.44-2.27 (m, 2H, H-7', H-2'), 2.05-1.93 (m, 1H, 1H, H-1'), 1.67-1.56 (m, 1H, H-4'a), 1.48 (ddd, *J* =13.0, 3.5, 3.5 Hz, 1H, H-5'), 1.27-1.14 (m, 1H, H-6'), 1.17-1.05 (m, 1H, H-8'), 1.06 (d, *J* = 6.0 Hz, 3H, 8'-Me), 0.97 (d, *J* = 7.5 Hz, 3H, 2'-Me), 0.93 (d, *J* = 7.0 Hz, 3H, 6'-Me), 0.83 (d, *J* = 6.5 Hz, 1H, OH), 0.74 (ddd, *J* = 10.5, 10.5, 8.0 Hz, 1H, H-8'a), 0.71 (ddd, *J* = 13.0, 13.0, 13.0 Hz, 1H, H-5');
^{13}C NMR (125 MHz, C_6D_6): δ = 166.3 (Bz), 133.8 (C-3'), 132.8 (Bz), 132.7 (C-4'), 131.2 (Bz), 129.9 (Bz), 128.6 (Bz), 82.0 (C-7'), 66.8 (C-1), 45.9 (C-8'a), 43.7 (C-1'), 43.3 (C-8'), 41.7 (C-4'a), 39.6 (C-6'), 39.0 (C-5'), 32.7 (C-2'), 19.2 (6'-Me), 17.8 (8'-Me), 16.1 (2'-Me);
HRMS: *m*/*z* $[M + Na]^+$ calcd for $C_{21}H_{28}O_3Na$: 351.1931; found: 351.1924.

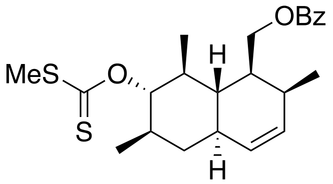

[(1'*S*,2'*S*,4'a*R*,6'*R*,7'*S*,8'*S*,8'a*S*)-7'-(Methylthiocarbonothioyloxy)-2',6',8'-trimethyl-1',2',4'a,5',6',7',8',8'a-octahydronaphthalen-1'-yl]methyl benzoate (8)

To a solution of alcohol **26** (500 mg, 1.52 mmol) in tetrahydrofuran (30.0 mL) at –78 °C, a 1.00 M solution of sodium bis(trimethylsilyl)amide in tetrahydrofuran (3.0 mL, 3.04 mmol) was added. After the reaction mixture was stirred for 30 min at –78 °C, carbon disulfide (0.28 mL, 4.57 mmol) was added to the reaction mixture, and the mixture was stirred for 1 h at –78 °C. Then, iodomethane (0.47 mL, 7.61 mmol) was added to the reaction mixture, and the mixture was stirred for 1.5 h at –78 °C. Afterwards, saturated aqueous ammonium chloride was added to the reaction mixture. The mixture was extracted with diethyl ether, the organic layer was washed with brine, and dried over sodium sulfate. After filtration of the mixture and concentration of the solvent, the crude product was purified by column chromatography on silica (eluant; hexane/ethyl acetate = 30/1) to afford xanthate **8** (636 mg, quant.) as white solid.
Mp: 79.0 °C;
IR (KBr): 3780, 1712, 1635, 1281 cm^{-1};
$[\alpha]_D^{20}$ –44.3 (*c* 1.17, $CHCl_3$);
^{1}H NMR (500 MHz, C_6D_6): δ = 8.23-8.16 (m, 2H, Bz), 7.14-7.05 (m, 3H, Bz), 5.56 (ddd, *J* = 9.0, 3.5, 3.5 Hz, 1H, H-3'), 5.53 (dd, *J* = 10.0, 10.0 Hz, 1H, H-7'), 5,37 (ddd, *J* = 9.0, 2.5, 2.5 Hz, 1H, H-4'), 4.30 (dd, *J* = 11.0, 5.5 Hz, 1H, H-1), 4.16 (dd, *J* = 11.0, 8.0 Hz, 1H, H-1), 2.31-2.20 (m, 1H, H-2'), 2.21 (s, 3H, SMe), 1.95-1.84 (m, 1H, H-1'), 1.68-1.46 (m, 3H, H-4'a, H-6', H-8'), 1.41 (ddd, *J* = 13.0, 3.5, 3.5 Hz, 1H, H-5'), 1.03 (d, *J* = 6.5 Hz, 3H, 8'-Me), 0.96 (d, *J* = 6.0 Hz, 3H, 6'-Me), 0.93 (d, *J* = 7.0 Hz, 3H, 2'-Me), 0.79 (ddd, *J* = 10.5, 10.5, 8.0 Hz, 1H, H-8'a), 0.74 (ddd, *J* = 13.0, 13.0, 13.0 Hz, 1H, H-5');
^{13}C NMR (125 MHz, C_6D_6): δ = 217.8 (C=S), 166.2 (Bz), 134.1 (C-3'), 132.9 (Bz), 132.0

(C-4’), 131.1 (Bz), 129.9 (Bz), 128.6 (Bz), 92.9 (C-7’), 66.5 (C-1), 45.7 (C-8’a), 43.4 (C-1’), 41.8 (C-4’a), 41.1 (C-6’), 38.4 (C-8’), 38.2 (C-5’), 32.6 (C-2’), 18.9 (SMe), 18.8 (6’-Me), 17.0 (8’-Me), 16.1 (2’-Me);
HRMS: *m/z* $[M + Na]^+$ calcd for $C_{23}H_{30}O_3S_2Na$: 441.1529; found: 441.1536.

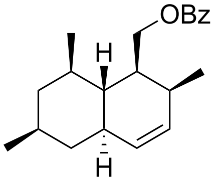

[(1'*S*,2'*S*,4'a*R*,6'*S*,8'*R*,8'a*S*)-2',6',8'-Trimethyl-1',2',4'a,5',6',7',8',8'a-octahydronaphthalen-1'-yl]methyl benzoate (7)

Azobis(isobutyronitrile) (22.3 mg, 0.135 mmol) and tributyltin hydride (0.72 mL, 2.71 mmol) were added to a solution of xanthate **8** (568 mg, 1.35 mmol) in toluene (13.5 mL) at room temperature. Then, the reaction mixture was stirred for 3 min at 110 °C, and it was filtered through a short pad of silica (eluant; hexane/ethyl acetate = 4/1). After concentration of the solvent, the crude product was purified by thin layer chromatography on silica (eluant; hexane/ethyl acetate = 20/1) and thin layer chromatography on silica (eluant; hexane/ethyl acetate = 20/1) to afford benzoate **7** (421 mg, quant.) as white solid.
Mp: 30.8 °C;
IR (neat): 2908, 2360, 1720, 1273 cm^{-1};
$[\alpha]_D^{21}$ +12.8 (*c* 0.887, $CHCl_3$);
^1H NMR (500 MHz, C_6D_6): δ = 8.25-8.18 (m, 2H, Bz), 7.15-7.03 (m, 3H, Bz), 5,62 (ddd, *J* = 9.0, 4.0, 3.0 Hz, 1H, H-3’), 5.51 (ddd, *J* = 9.0, 2.5, 2.5 Hz, 1H, H-4’), 4.49 (dd, *J* = 11.0, 5.0 Hz, 1H, H-1), 4.29 (dd, *J* = 11.0, 8.5 Hz, 1H, H-1), 2.50-2.34 (m, 1H, H-2’), 2.12-1.96 (m, 1H, H-1’), 1.74-1.61 (m, 1H, H-4’a), 1.63-1.49 (m, 1H, H-7’), 1.55-1.41 (m, 1H, H-5’), 1.37-1.16 (m, 2H, H-6’, H-8’), 0.98 (d, *J* = 7.5 Hz, 3H, 2’-Me), 0.90 (d, *J* = 6.5 Hz, 3H, 8’-Me), 0.82 (d, *J* = 6.5 Hz, 3H, 6’-Me), 0.73-0.60 (m, 2H, H-7’, H-8’a), 0.61-0.49 (m, 1H, H-5’);
^{13}C NMR (125 MHz, C_6D_6): δ = 166.3 (Bz), 133.2 (C-3’, C-4’), 132.8 (Bz), 131.3 (Bz), 129.9 (Bz), 128.6 (Bz), 67.0 (C-1), 46.63 (C-8’a), 46.58 (C-5’), 43.7 (C-1’), 42.9 (C-4’a), 41.5 (C-7’), 35.5 (C-8’), 33.0 (C-2’), 32.4 (C-6’), 22.6 (6’-Me), 22.3 (8’-Me), 16.0 (2’-Me);
HRMS: *m/z* $[M + Na]^+$ calcd for $C_{21}H_{28}O_2Na$: 335.1982; found: 335.1967.

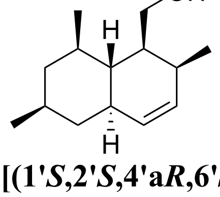

[(1'*S*,2'*S*,4'a*R*,6'*S*,8'*R*,8'a*S*)-2',6',8'-Trimethyl-1',2',4'a,5',6',7',8',8'a-octahydronaphthalen-1'-yl]methanol (27)

To a solution of the benzoate **7** (359 mg, 1.14 mmol) in dichloromethane (11.0 mL) at 0 °C, a 1.00 M solution of diisobutylaluminum hydride in hexane (2.87 mL, 2.87 mmol) was

added. After the mixture was stirred for 20 min at 0 °C, methanol and saturated aqueous Rochelle salt were added. The mixture was extracted with dichloromethane, and dried over sodium sulfate. After filtration of the mixture and concentration of the solvent, the crude product was purified by thin layer chromatography on silica (eluant; hexane/ethyl acetate = 4/1) to afford alcohol **27** (232 mg, 98%) as colorless oil.
IR (neat): 3363, 1458 cm^{-1};
$[\alpha]_D^{28}$ –30.9 (*c* 1.00, $CHCl_3$);
^{1}H NMR (500 MHz, C_6D_6): δ = 5.66 (ddd, *J* = 9.5, 4.0, 3.0 Hz, 1H, H-3'), 5.51 (ddd, *J* = 9.5, 2.5, 2.5 Hz, 1H, H-4'), 3.68-3.54 (m, 1H, H-1), 3.46-3.30 (m, 1H, H-1), 2.45-2.30 (m, 1H, H-2'), 1.72-1.60 (m, 2H, 1'-H, H-4'a), 1.65-1.55 (m, 1H. H-7'), 1.58-1.46 (m, 1H, H-5'), 1.40-1.23 (m, 1H, H-6'), 1.29-1.14 (m, 1H, H-8'), 1.01 (d, *J* = 7.0 Hz, 3H, 2'-Me), 0.85 (d, *J* = 6.5 Hz, 3H, 8'-Me), 0.84 (d, *J* = 6.5 Hz, 3H, 6'-Me), 0.76-0.52 (m, 4H, H-7', H-8'a, OH, H-5');
^{13}C NMR (125 MHz, C_6D_6): δ = 134.0 (C-3'), 133.7 (C-4'), 64.6 (C-1), 46.9 (C-1'), 46.8 (C-8'a), 46.7 (C-5'), 42.9 (C-4'a), 41.5 (C-7'), 35.8 (C-8'), 32.5 (C-2', C-6'), 22.6 (8'-Me), 22.1 (6'-Me), 16.0 (2'-Me);
HRMS: *m*/*z* $[M + Na]^+$ calcd for $C_{14}H_{28}ONa$: 231.1719; found: 231.1719.

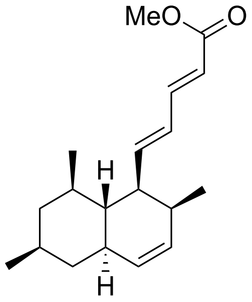

Methyl (2*E*,4*E*)-5-[(1'*S*,2'*S*,4'a*R*,6'*S*,8'*R*,8'a*S*)-2',6',8'-trimethyl-1',2',4'a,5',6',7',8',8'a-octahydronaphthalen-1'-yl]penta-2,4-dienoate (5)

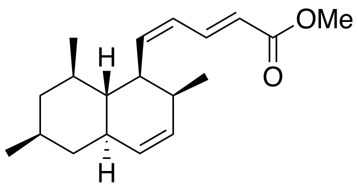

Methyl (2*E*,4*Z*)-5-[(1'*S*,2'*S*,4'a*R*,6'*S*,8'*R*,8'a*S*)-2',6',8'-trimethyl-1',2',4'a,5',6',7',8',8'a-octahydronaphthalen-1'-yl]penta-2,4-dienoate (29)

To a solution of alcohol **27** (133 mg, 0.638 mmol) and Molecular Sieves 4A (191 mg) in dichloromethane (6.4 mL) at 0 °C, 4-methylmorpholine *N*-oxide (224 mg, 1.91 mmol) and tetrapropylammonium perruthenate (22.4 mg, 0.0638 mmol) were added. Then, the reaction mixture was stirred for 30 min at 0 °C, and it was filtered through a short pad of silica (eluant; hexane/ethyl acetate = 4/1). After concentration of the solvent, the residue was

dried under reduced pressure to afford crude aldehyde **6**, which was used in the following reaction without further purification.

A 1.00 M solution of lithium bis(trimethylsilyl)amide in tetrahydrofuran (3.57 mL, 3.57 mmol) was added to a solution of phosphate **28** (904 mg, 3.83 mmol) in tetrahydrofuran (9.0 mL) at –78 °C. Then, the reaction mixture was stirred for 30 min at –78 °C, and a solution of the crude aldehyde **6** in tetrahydrofuran (3.8 mL) was added to the reaction mixture. After the reaction mixture was stirred for 3 h at room temperature, saturated aqueous ammonium chloride was added at 0 °C. The mixture was extracted with ethyl acetate, and the organic layer was dried over sodium sulfate. After filtration of the mixture and concentration of the solvent, it was filtered through a short pad of silica (eluant; hexane/ethyl acetate = 4/1). After concentration of the solvent, the residue was purified by thin layer chromatography on silica (eluant; cyclohexane/toluene = 7/3 × 4) to afford ester **5** (22.4 mg, 12%) as colorless oil and a mixture of ester **5** [105 mg, 57% (calculated from ^{1}H NMR spectra)] and ester **29** [18.4 mg, 10% (calculated from ^{1}H NMR spectra)]. Then, a mixture of ester **5** and ester **29** (70.9 mg) was purified by thin layer chromatography on silica (eluant; cyclohexane/toluene = 7/3 × 4) to afford ester **5** (21.8 mg, 12%) as colorless oil.

Isolated Yield: 44.2 mg, 24% (ester **5**); Yield (calculated from ^{1}H NMR spectra): 127 mg, 69% (ester **5**), 18.4 mg, 10% (ester **29**);

Methyl (2*E*,4*E*)-5-[(1’*S*,2’*S*,4’a*R*,6’*S*,8’*R*,8’a*S*)-2’,6’,8’-trimethyl-1’,2’,4’a,5’,6’,7’,8’,8’a-octahydronaphthalen-1’-yl]penta-2,4-dienoate (5)

IR (neat): 1720, 1643, 1142 cm^{-1};

$[\alpha]_D^{25}$ +44.8 (*c* 1.00, $CHCl_3$);

^{1}H NMR (500 MHz, C_6D_6): δ = 7.49 (dd, *J* = 15.5, 10.5 Hz, 1H, H-3), 5.95 (dd, *J* = 14.5, 10.5 Hz, 1H, H-5), 5.87 (d, *J* = 15.5 Hz, 1H, H-2), 5.80 (dd, *J* = 14.5, 10.5 Hz, 1H, H-4), 5.53 (ddd, *J* = 9.5, 4.5, 3.0 Hz, 1H, H-3’), 5.44 (ddd, *J* = 9.5, 2.0, 2.0 Hz, 1H, H-4’), 3.46 (s, 3H, OMe), 2.18 (ddd, *J* = 10.5, 10.5, 5.5 Hz, 1H, H-1’), 2.10-1.94 (m, 1H, H-2’), 1.73-1.57 (m, 1H, H-4’a), 1.64-1.43 (m, 1H, H-7’), 1.54-1.40 (m, 1H, H-5’), 1.45-1.20 (m, 1H, H-6’), 1.25-1.03 (m, 1H, H-8’), 0.84 (d, *J* = 6.5 Hz, 3H, 6’-Me), 0.82 (d, *J* = 5.5 Hz, 3H, 2’-Me), 0.80 (d, *J* = 4.5 Hz, 3H, 8’-Me), 0.84-0.51 (m, 3H, H-5’, H-7’, H-8’a);

^{13}C NMR (125 MHz, C_6D_6): δ = 167.2 (C-1), 150.0 (C-5), 145.2 (C-3), 132.3 (C-3’, C-4’), 126.9 (C-4), 119.5 (C-2), 51.0 (OMe), 49.5 (C-1’), 46.9 (C-5’), 46.8 (C-8’a), 43.0 (C-4’a), 42.0 (C-7’), 37.2 (C-2’), 36.7 (C-8’), 32.7 (C-6’), 22.9 (8’-Me), 22.5 (6’-Me), 16.6 (2’-Me);

HRMS: *m*/*z* [M + Na]$^{+}$ calcd for $C_{19}H_{28}O_2Na$: 311.1982; found: 311.1978.

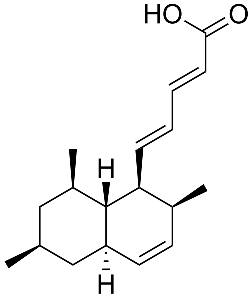

(2*E*,4*E*)-5-[(1'*S*,2'*S*,4'a*R*,6'*S*,8'*R*,8'a*S*)-2',6',8'-trimethyl-1',2',4'a,5',6',7',8',8'a-octahydronaphthalen-1'-yl]penta-2,4-dienoic acid [Tanzawaic acid B] (2)

To a solution of the ester **5** (32.9 mg, 0.114 mmol) in methanol (0.5 mL) and tetrahydrofuran (1.0 mL) at 0 °C, 4 M aqueous lithium hydroxide (0.50 mL, 2.00 mmol) was added. After the reaction mixture was stirred for 4 h at room temperature, 1.0 M aqueous hydrochloride was added at 0 °C. The mixture was extracted with ethyl acetate, and the organic layer was dried over sodium sulfate. After filtration of the mixture and concentration of the solvent, the crude product was purified by thin layer chromatography on silica (eluant; chloroform/methanol/acetic acid = 170/10/1 × 2) to afford tanzawaic acid B (**2**) (22.3 mg, 71%) as slightly pale yellow solid.

Mp: 129 °C;

IR (KBr): 3471, 2908, 1682, 1612, 1273 cm^{-1};

$[\alpha]_D^{25}$ +57.7 (*c* 1.19, $CHCl_3$);

^1H NMR (500 MHz, C_6D_6): δ = 7.48 (dd, *J* = 15.5, 11.0 Hz, 1H, H-3), 5.95 (dd, *J* = 15.0, 10.0 Hz, 1H, H-5), 5.79 (d, *J* = 15.5 Hz, 1H, H-2), 5.73 (dd, *J* = 15.0, 11.0 Hz, 1H, H-4), 5.53 (ddd, *J* = 9.5, 4.0, 2.5 Hz, 1H, H-3'), 5.44 (br ddd, *J* = 9.5, 2.0, 2.0 Hz, 1H, H-4'), 2.16 (ddd, *J* = 10.0, 10.0, 5.5 Hz, 1H, H-1'), 2.07-1.92 (m, 1H, H-2'), 1.72-1.57 (m, 1H, H-4'a), 1.63-1.49 (m, 1H, H-7'), 1.53-1.40 (m, 1H, H-5'), 1.44-1.17 (m, 1H, H-6'), 1.23-1.02 (m, 1H, H-8'), 0.84 (d, *J* = 6.0 Hz, 3H, 6'-Me), 0.80 (d, *J* = 7.0 Hz, 3H, 2'-Me), 0.79 (d, *J* = 7.0 Hz, 3H, 8'-Me), 0.80-0.51 (m, 3H, H-5', H-7', H-8'a);

^{13}C NMR (125 MHz, C_6D_6): δ = 173.4 (C-1), 151.4 (C-5), 147.6 (C-3), 132.4 (C-4'), 132.3 (C-3'), 126.8 (C-4), 119.0 (C-2), 49.5 (C-1'), 47.0 (C-5'), 46.8 (C-8'a), 42.9 (C-4'a), 41.9 (C-7'), 37.1 (C-2'), 36.7 (C-8'), 32.7 (C-6'), 22.8 (8'-Me), 22.6 (6'-Me), 16.6 (2'-Me);

HRMS: *m*/*z* $[M-H]^-$ calcd for $C_{18}H_{25}O_2$: 273.1849; found: 273.1862.

Comparison of ^{1}H and ^{13}C NMR Spectra.

Table S1. Comparison of ^{1}H and ^{13}C NMR Spectra between Synthetic Sample and Natural Tanzawaic Acid B.

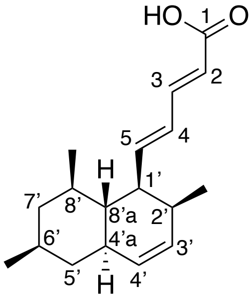

	synthetic tanzawaic acid B			natural tanzawaic acid B	
position	δ_C[a]	δ_H (J in Hz)[b]	position	δ_C[c]	δ_H (J in Hz)[d]
1	172.7		1	173.0	
2	118.0	5.78, d (15.0)	2	118.2	5.78, d (15.2)
3	147.5	7.38, dd (15.0, 11.0)	3	147.7	7.38, dd (15.2, 11.0)
4	126.3	6.13, dd (15.5, 11.0)	4	126.4	6.12, dd (15.0, 11.0)
5	152.0	6.29, dd (15.5, 10.0)	5	152.2	6.29, dd (15.0, 10.2)
1'	49.3	2.43, ddd (10.0, 10.0, 5.5)	1'	49.5	2.42, ddd (15.5, 10.2, 5.4)
2'	36.9	2.25-2.14, m	2'	37.1	2.18, m
2'-Me	16.5	0.94, d (7.0)	2'-Me	16.6	0.94, d (7.4)
3'	132.0	5.56, ddd (9.5, 4.0, 2.5)	3'	132.2	5.56, ddd (9.6, 4.4, 2.9)
4'	132.2	5.44, ddd (9.5, 2.0, 2.0)	4'	132.4	5.44, dt (9.6, 1.8)
4'a	42.7	1.87-1.74, m	4'a	42.8	1.80, tdd (12.5, 5.3, 2.5)
5'	46.8	1.69-1.58, m	5'	47.0	1.64, ddd (13.4, 5.6, 3.4)
		0.96-0.87, m			0.92, m
6'	32.5	1.60-1.42, m	6'	32.6	1.52, m
6'-Me	22.8	0.88, d (6.5)	6'-Me	22.9	0.87, d (6.5)
7'	41.7	1.76-1.67, m	7'	41.9	1.72, ddd (12.7, 5.3, 3.4)
		0.84-0.68, m			0.79, q (12.7)
8'	36.5	1.45-1.28, m	8'	36.7	1.36, m
8'-Me	22.4	0.90, d (6.5)	8'-Me	22.5	0.89, d (6.5)
8'a	46.7	0.84-0.68, m	8'a	46.8	0.75, q (13.4)

a : 500 MHz.
b : 125 MHz.
c : 600 MHz. See Ref 5.
d : 150 MHz. See Ref 5.

^{1}H and ^{13}C NMR spectra of synthetic tanzawaic acid B were different from those of natural tanzawaic acid B[5].

The misassignment of ^{1}H NMR spectra of natural tanzawaic acid B were confirmed from our HMQC and HMBC spectra. See the following information:

(i) The signals of H-5’ in the isolation report corresponded to the signals assigned to H-7’ and H-8’a in our assignment of ^{1}H NMR spectra;
(ii) The signals of H-7’ in the isolation report corresponded to the signals assigned to H-5’ in our assignment of ^{1}H NMR spectra;
(iii) The signal of H-8’a in the isolation report corresponded to the signal assigned to H-7’ in our assignment of ^{1}H NMR spectra.
Therefore, signals of H-5’, H-7’, and H-8’a in ^{1}H NMR spectra were corrected as table S1.

In the case of ^{13}C NMR spectra, the misassignment in isolation report was also confirmed from our HMQC and HMBC spectra. See the following information:
(i) The signal of C-5’ in the isolation report corresponded to the signal of C-8’a in our assignment of ^{13}C NMR spectrum;
(ii) The signal of C-7’ in the isolation report corresponded to the signal of C-5’ in our assignment of ^{13}C NMR spectrum;
(iii) The signal of C-8’a in the isolation report corresponded to the signal assigned to C-7’ in our assignment of ^{13}C NMR spectrum;
(iv) The signal of 6’-Me in the isolation report corresponded to the signal assigned to 8’-Me in our assignment of ^{13}C NMR spectrum;
(v) The signal of 8’-Me in the isolation report corresponded to the signal assigned to 6’-Me in our assignment of ^{13}C NMR spectrum.
Therefore, signals of C-5’, C-7’, C-8’a, 6’-Me, and 8’-Me in ^{13}C NMR spectra were corrected as table S1.

References.

1. W. Tseng, S. Chuang, Z. Hung, C. Wu, *European Patent Organization*, **2011**, EP2345645 A1.
2. E. J. Corey, H. Cho, C. Rucker, D. H. Hua, *Tetrahedron Lett.*, **1981**, *22*, *36*, 3455–3458.
3. I. Shiina, Y. Umezaki, T. Murata, K. Suzuki, T. Tonoi, *Synthesis*, **2018**, *50*, 1301–1306.
4. (a) R. Sarpong, J. T. Su, B. M. Stoltz, *J. Am. Chem. Soc.*, **2003**, *125*, 13624–13625. (b) D. F. Taber, P. Guo, N. Guo, *J. Am. Chem. Soc.*, **2010**, *132*, 11179–11182. (c) H. Bross, R. Schneider, H. Hopf, *Tetrahedron Lett.*, **1979**, *20*, 2129–2132.
5. J. Wang, T. Li, P. Wang, W. Ding, J. Xu, *J. Nat. Prod.*, **2022**, *85*, 1218–1228.

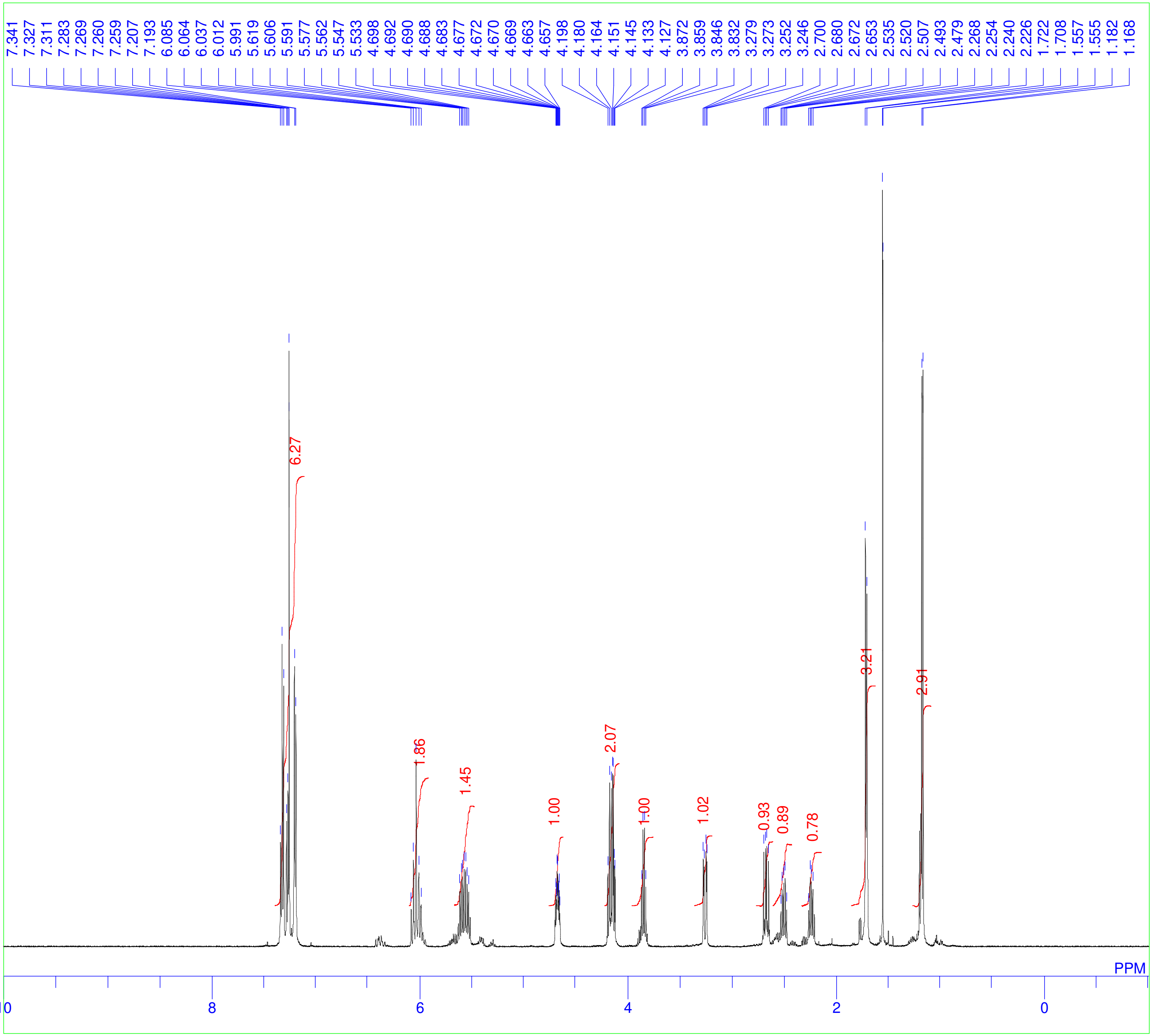

DFILE	14_1H.als
COMNT	
DATIM	Wed May 18 16:05:57 2011
OBNUC	1H
EXMOD	non
OBFRQ	500.00 MHz
OBSET	0.00 KHz
OBFIN	162160.00 Hz
POINT	32768
FREQU	10000.00 Hz
SCANS	16
ACQTM	3.2768 sec
PD	3.7232 sec
PW1	6.50 usec
IRNUC	1H
CTEMP	24.6 c
SLVNT	CDCL3
EXREF	7.26 ppm
BF	0.12 Hz
RGAIN	21

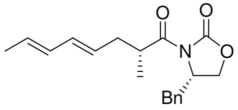

14

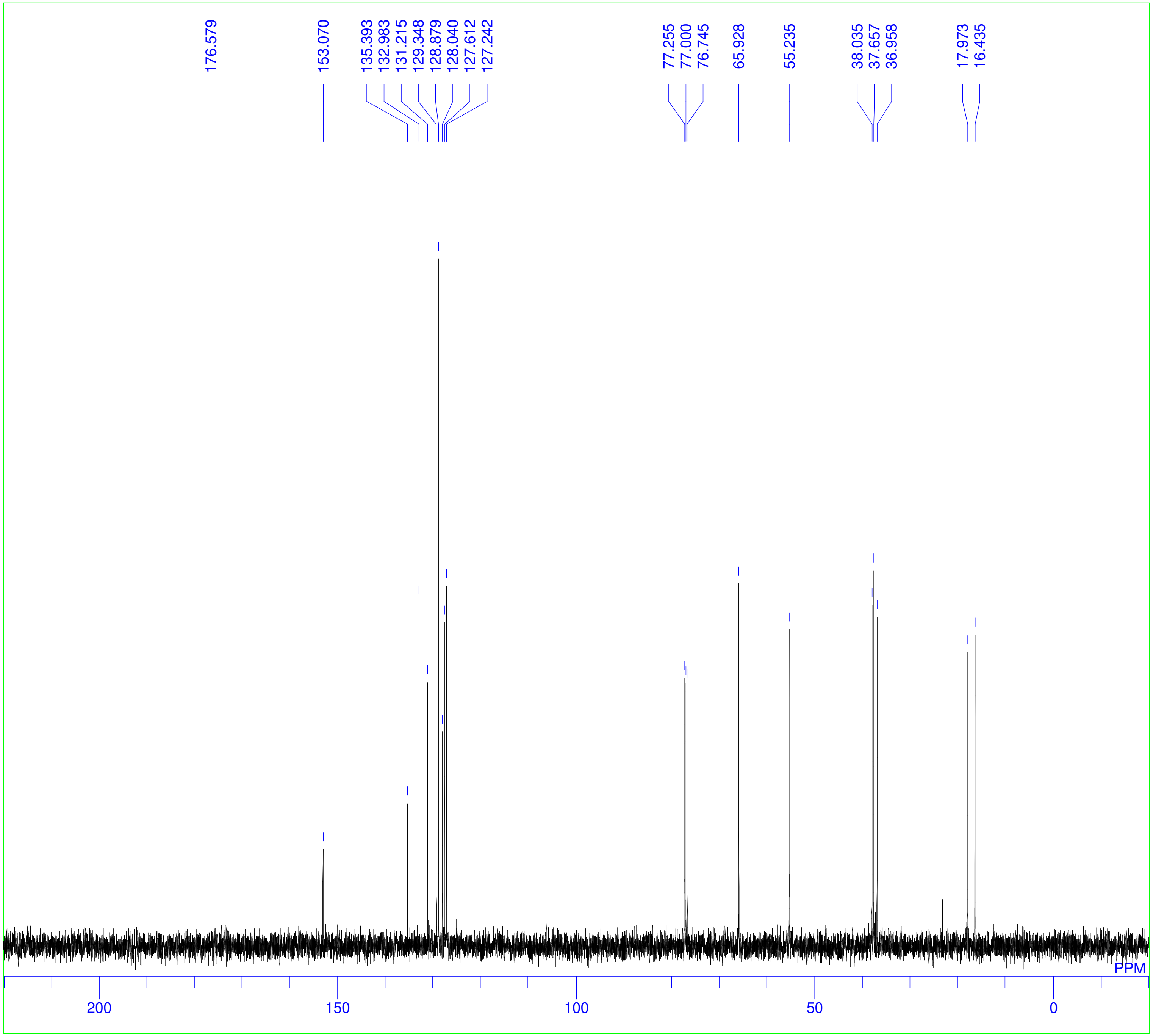

DFILE	14_13C.als
COMNT	
DATIM	Mon May 16 12:59:56 2011
OBNUC	13C
EXMOD	bcm
OBFRQ	125.65 MHz
OBSET	0.00 KHz
OBFIN	127958.00 Hz
POINT	32768
FREQU	33898.30 Hz
SCANS	64
ACQTM	0.9667 sec
PD	2.0333 sec
PW1	5.10 usec
IRNUC	1H
CTEMP	24.7 c
SLVNT	CDCL3
EXREF	77.00 ppm
BF	1.20 Hz
RGAIN	30

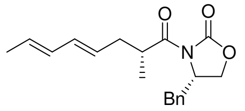

14

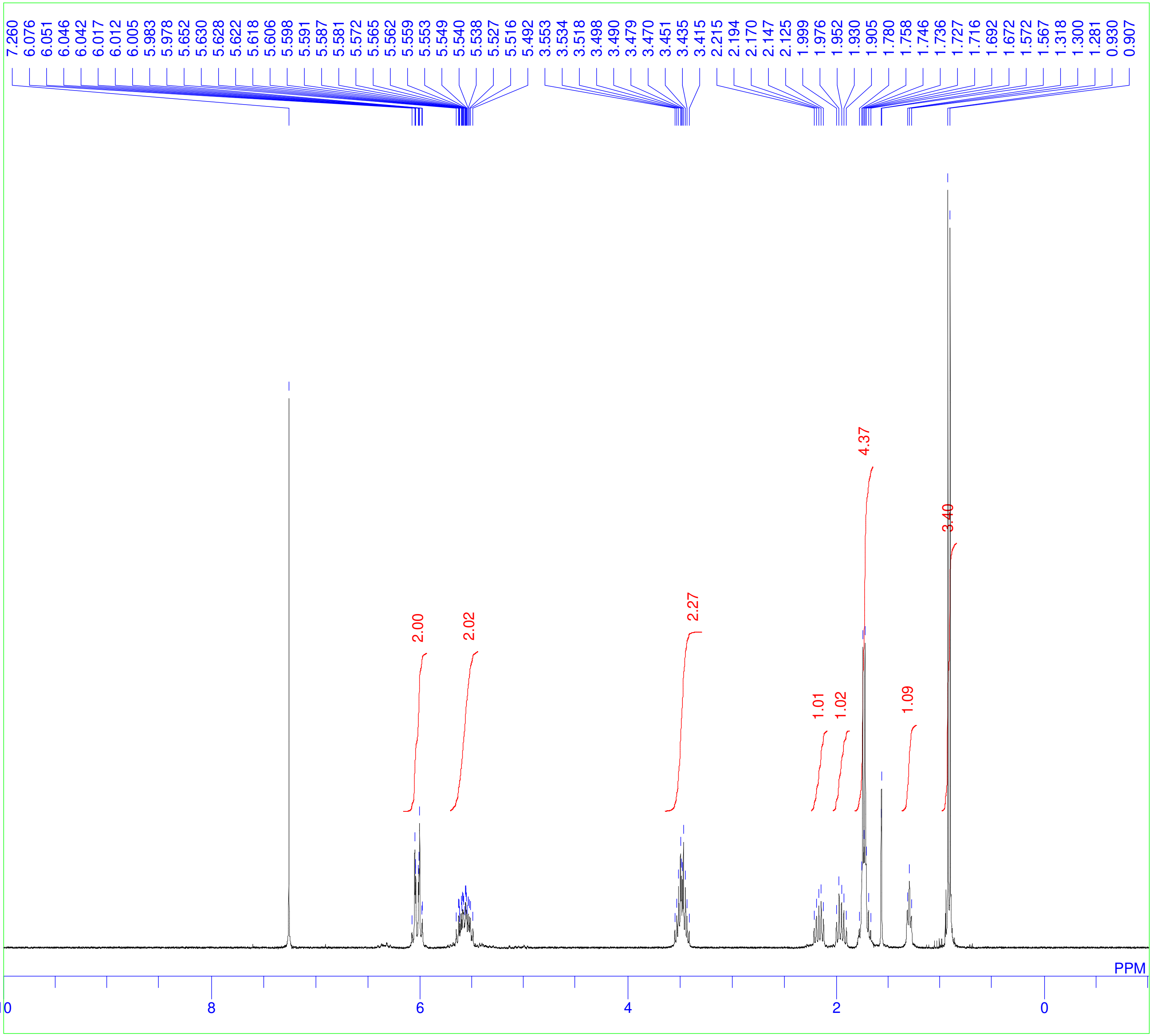

DFILE	15_1H.als
COMNT	
DATIM	Sat May 07 21:42:46 2011
OBNUC	1H
EXMOD	NON
OBFRQ	300.40 MHz
OBSET	130.00 KHz
OBFIN	1150.00 Hz
POINT	32768
FREQU	6020.40 Hz
SCANS	16
ACQTM	5.4428 sec
PD	1.5510 sec
PW1	5.60 usec
IRNUC	1H
CTEMP	20.6 c
SLVNT	CDCL3
EXREF	7.26 ppm
BF	0.12 Hz
RGAIN	21

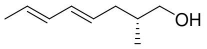

15

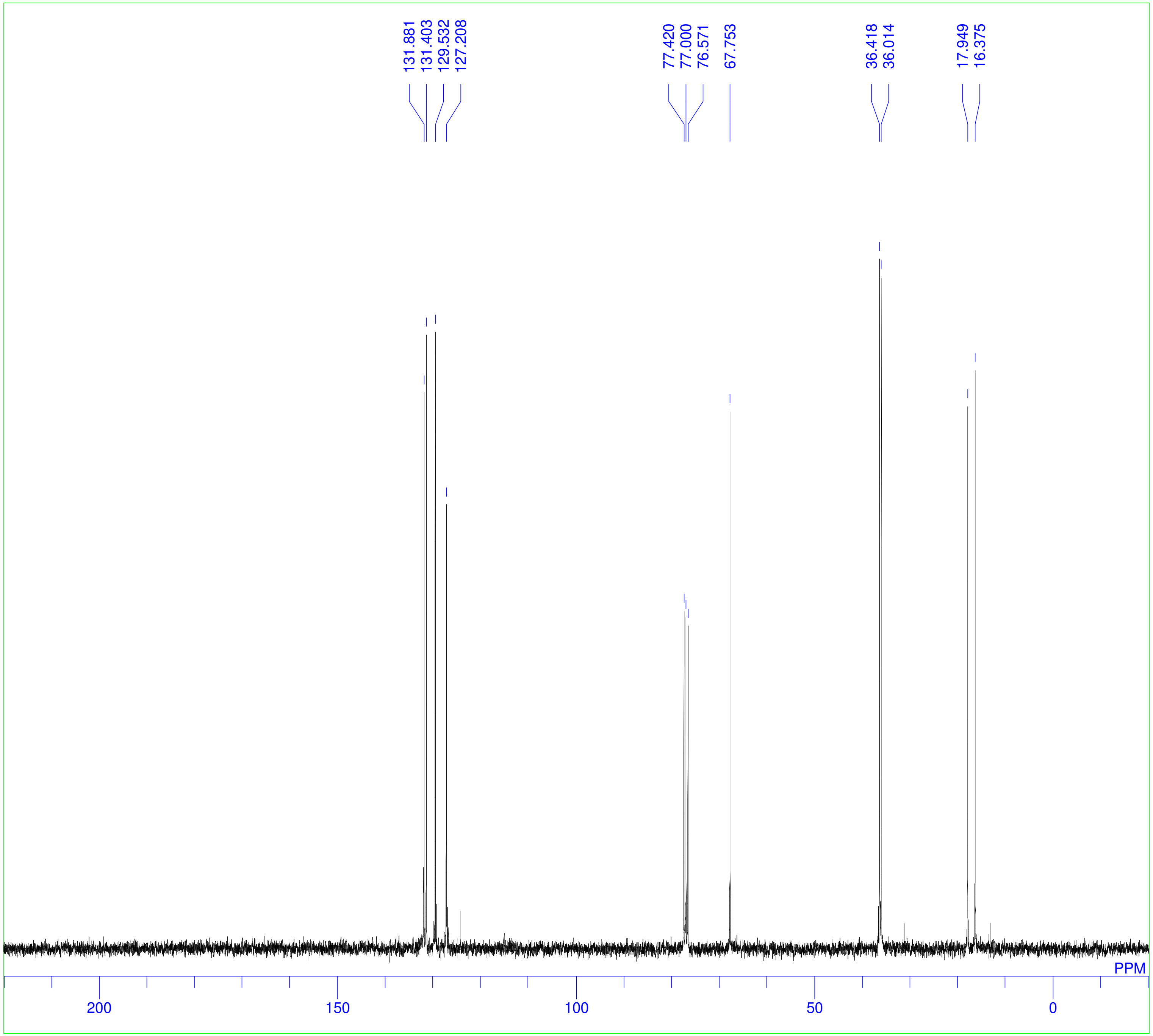

DFILE	15_13C.als
COMNT	
DATIM	Sat May 07 16:22:03 2011
OBNUC	13C
EXMOD	BCM
OBFRQ	75.45 MHz
OBSET	124.00 KHz
OBFIN	1840.00 Hz
POINT	32768
FREQU	20408.10 Hz
SCANS	256
ACQTM	1.6056 sec
PD	1.3940 sec
PW1	4.20 usec
IRNUC	1H
CTEMP	21.2 c
SLVNT	CDCL3
EXREF	77.00 ppm
BF	1.20 Hz
RGAIN	22

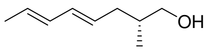

15

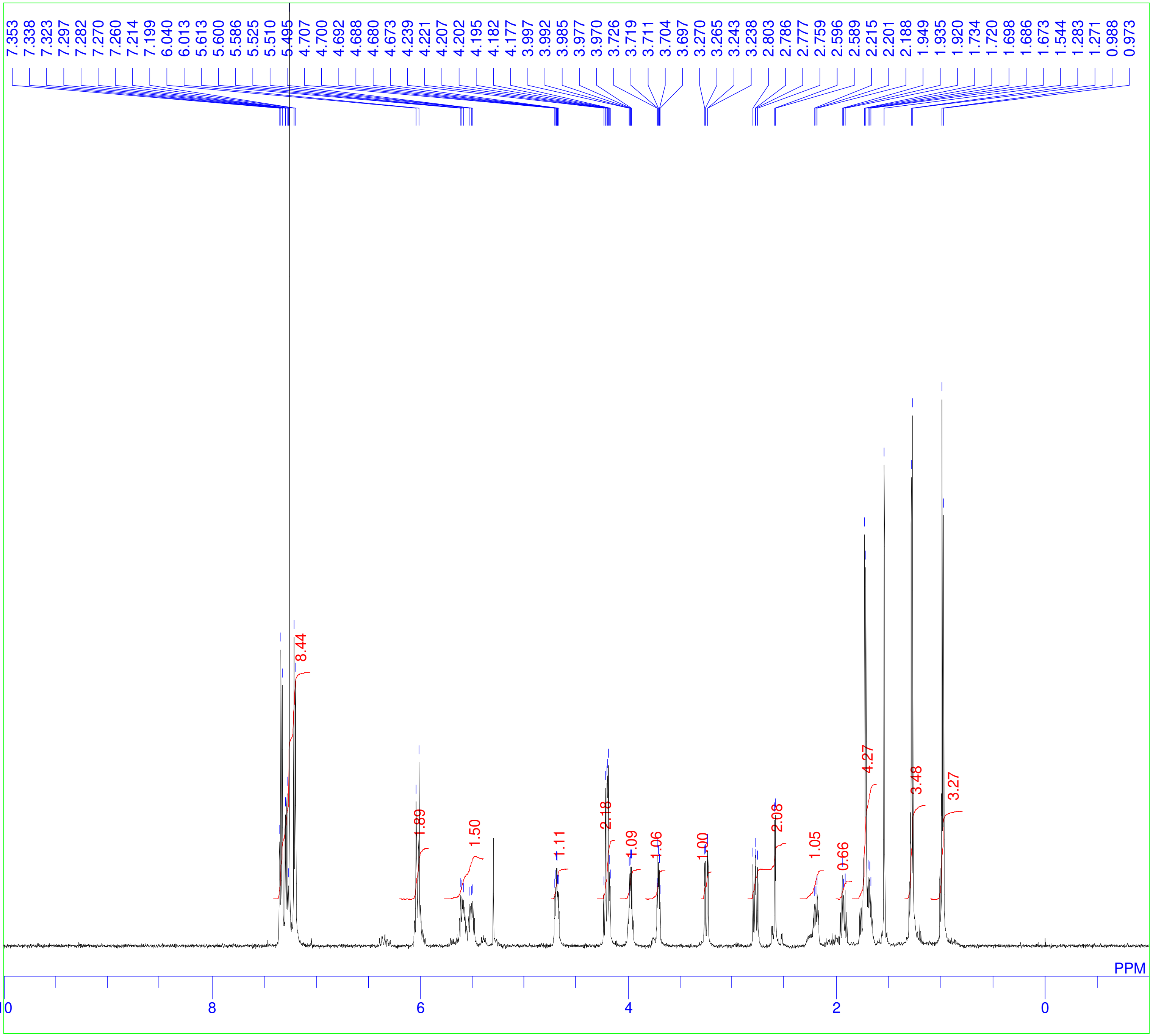

DFILE	17_1H.als
COMNT	
DATIM	Thu Jan 12 10:27:36 2012
OBNUC	1H
EXMOD	non
OBFRQ	500.00 MHz
OBSET	0.00 KHz
OBFIN	162160.00 Hz
POINT	8192
FREQU	10000.00 Hz
SCANS	8
ACQTM	0.8192 sec
PD	6.1808 sec
PW1	6.20 usec
IRNUC	1H
CTEMP	22.8 c
SLVNT	CDCL3
EXREF	7.26 ppm
BF	0.12 Hz
RGAIN	25

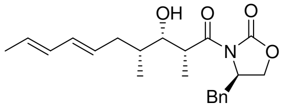

17

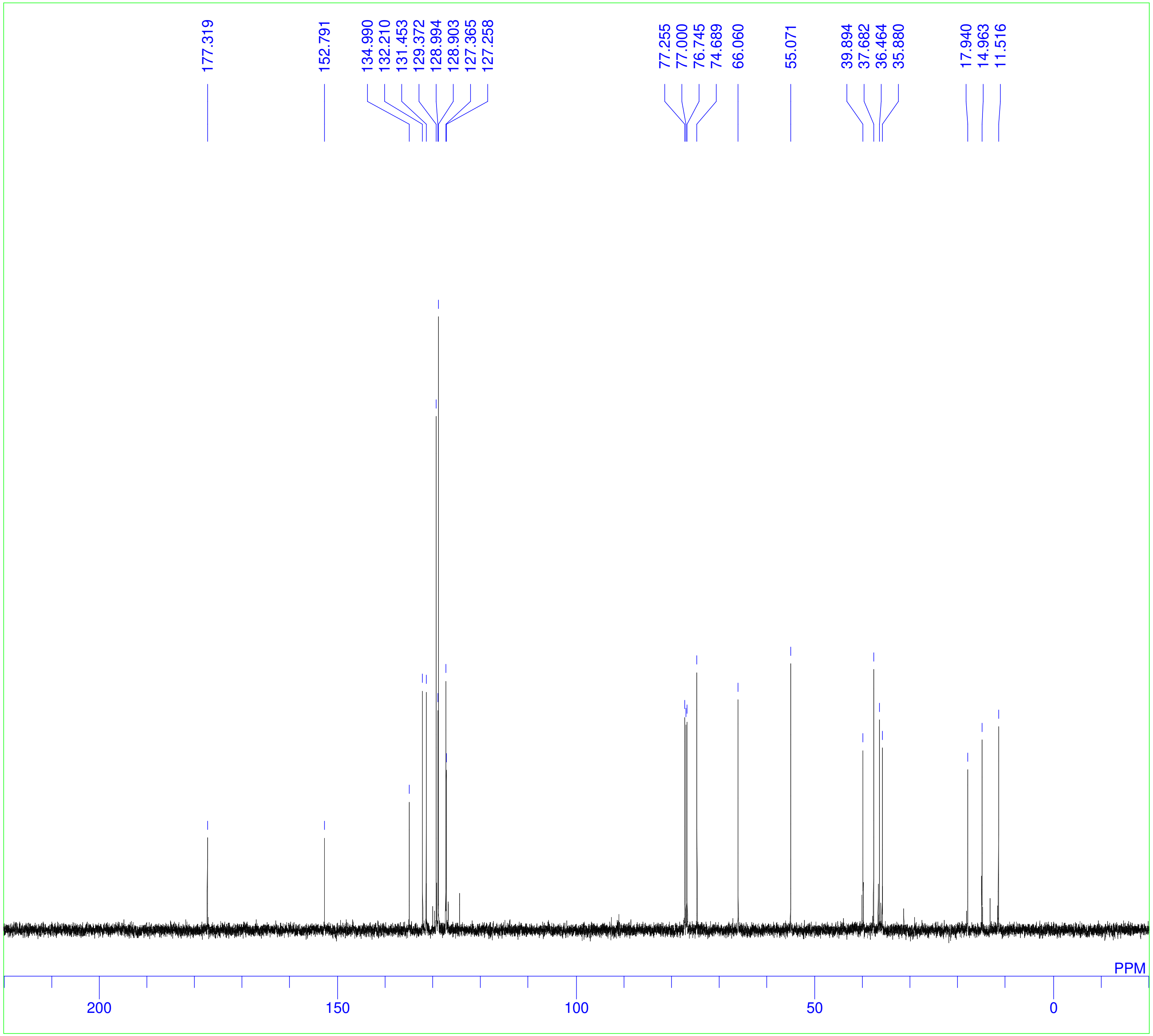

DFILE 17_13C.als
COMNT
DATIM Sun May 27 12:45:22 2012
OBNUC 13C
EXMOD bcm
OBFRQ 125.65 MHz
OBSET 0.00 KHz
OBFIN 127958.00 Hz
POINT 32768
FREQU 33898.30 Hz
SCANS 128
ACQTM 0.9667 sec
PD 2.0333 sec
PW1 4.90 usec
IRNUC 1H
CTEMP 26.1 c
SLVNT CDCL3
EXREF 77.00 ppm
BF 1.20 Hz
RGAIN 30

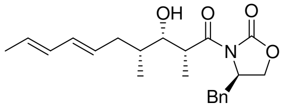

17

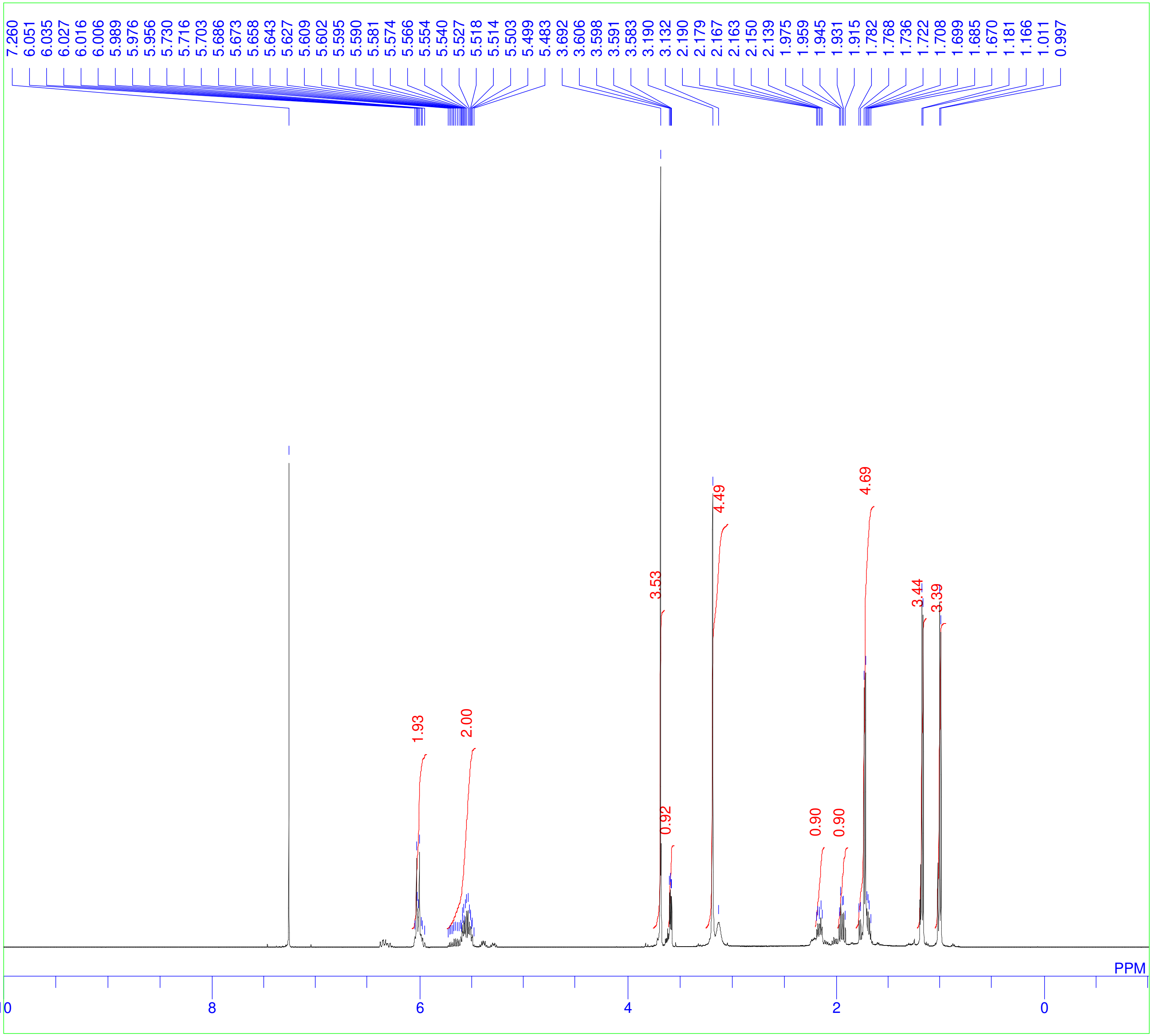

DFILE	18_1H.als
COMNT	
DATIM	2022-10-04 18:39:39
OBNUC	1H
EXMOD	proton.jxp
OBFRQ	500.16 MHz
OBSET	2.41 KHz
OBFIN	6.01 Hz
POINT	13107
FREQU	7507.51 Hz
SCANS	8
ACQTM	1.7459 sec
PD	5.0000 sec
PW1	3.84 usec
IRNUC	1H
CTEMP	23.8 c
SLVNT	CDCL3
EXREF	7.26 ppm
BF	0.25 Hz
RGAIN	44

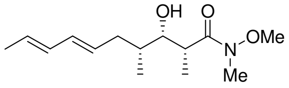

18

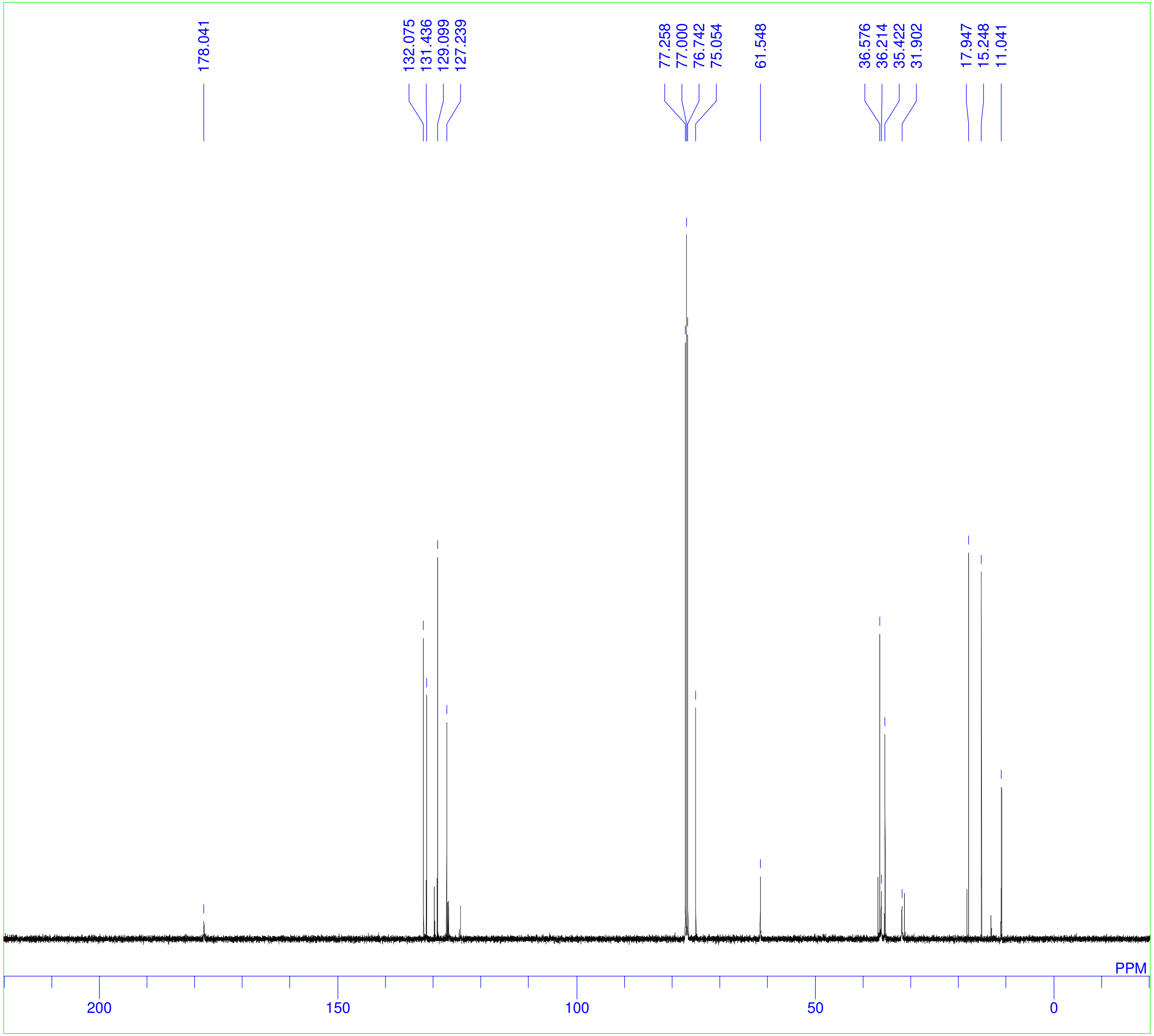

DFILE	18_13C.als
COMNT	
DATIM	2022-10-04 18:46:20
OBNUC	13C
EXMOD	carbon.jxp
OBFRQ	125.77 MHz
OBSET	7.87 KHz
OBFIN	4.21 Hz
POINT	26214
FREQU	31446.54 Hz
SCANS	1024
ACQTM	0.8336 sec
PD	2.0000 sec
PW1	3.87 usec
IRNUC	1H
CTEMP	24.1 c
SLVNT	CDCL3
EXREF	77.00 ppm
BF	0.25 Hz
RGAIN	30

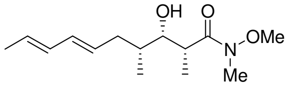

18

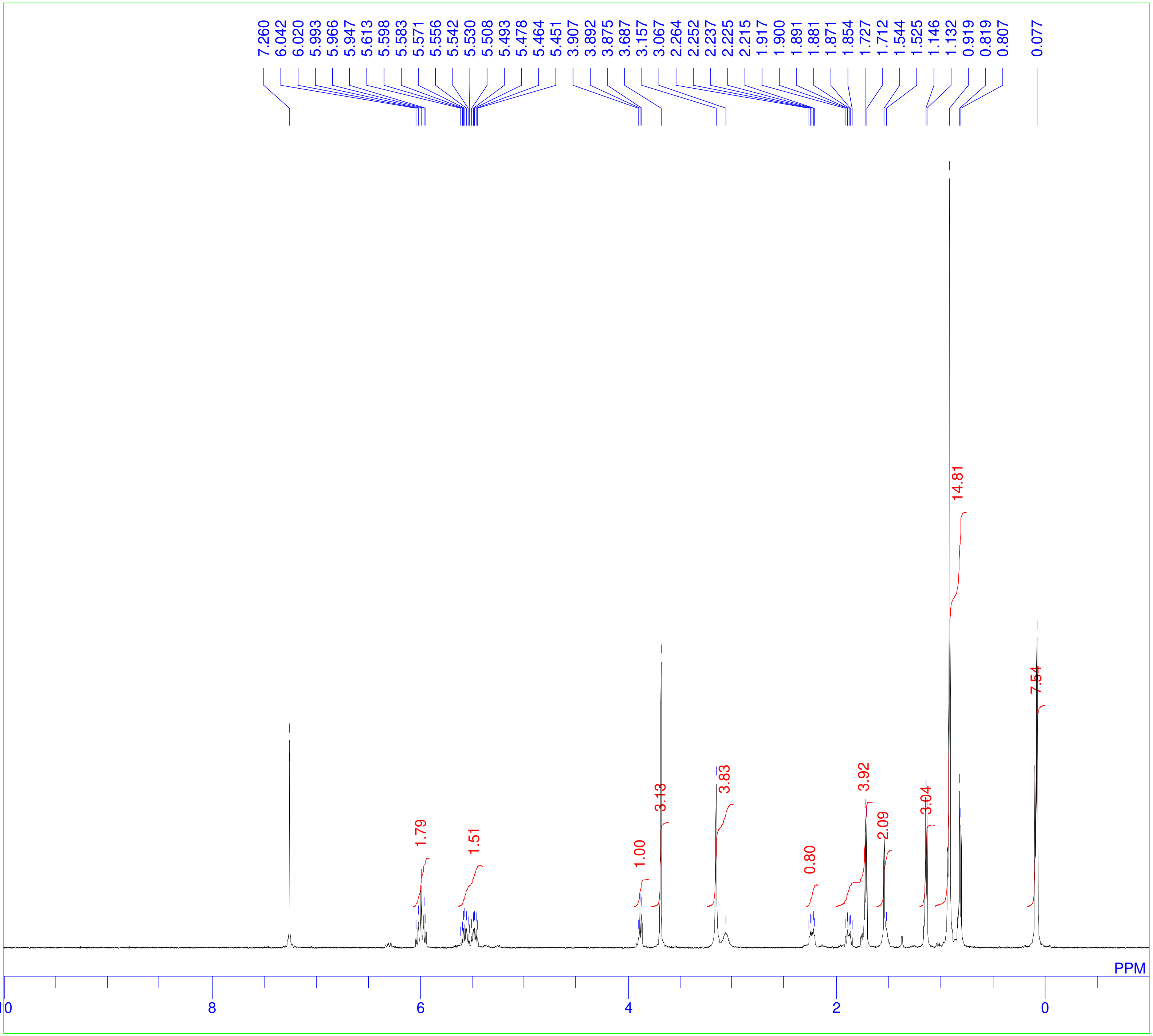

DFILE	19_1H.als
COMNT	
DATIM	Tue Jan 17 12:09:35 2012
OBNUC	1H
EXMOD	non
OBFRQ	500.00 MHz
OBSET	0.00 KHz
OBFIN	162160.00 Hz
POINT	8192
FREQU	10000.00 Hz
SCANS	8
ACQTM	0.8192 sec
PD	6.1808 sec
PW1	6.20 usec
IRNUC	1H
CTEMP	23.1 c
SLVNT	CDCL3
EXREF	7.26 ppm
BF	0.12 Hz
RGAIN	23

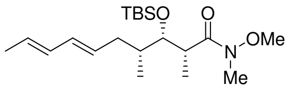

19

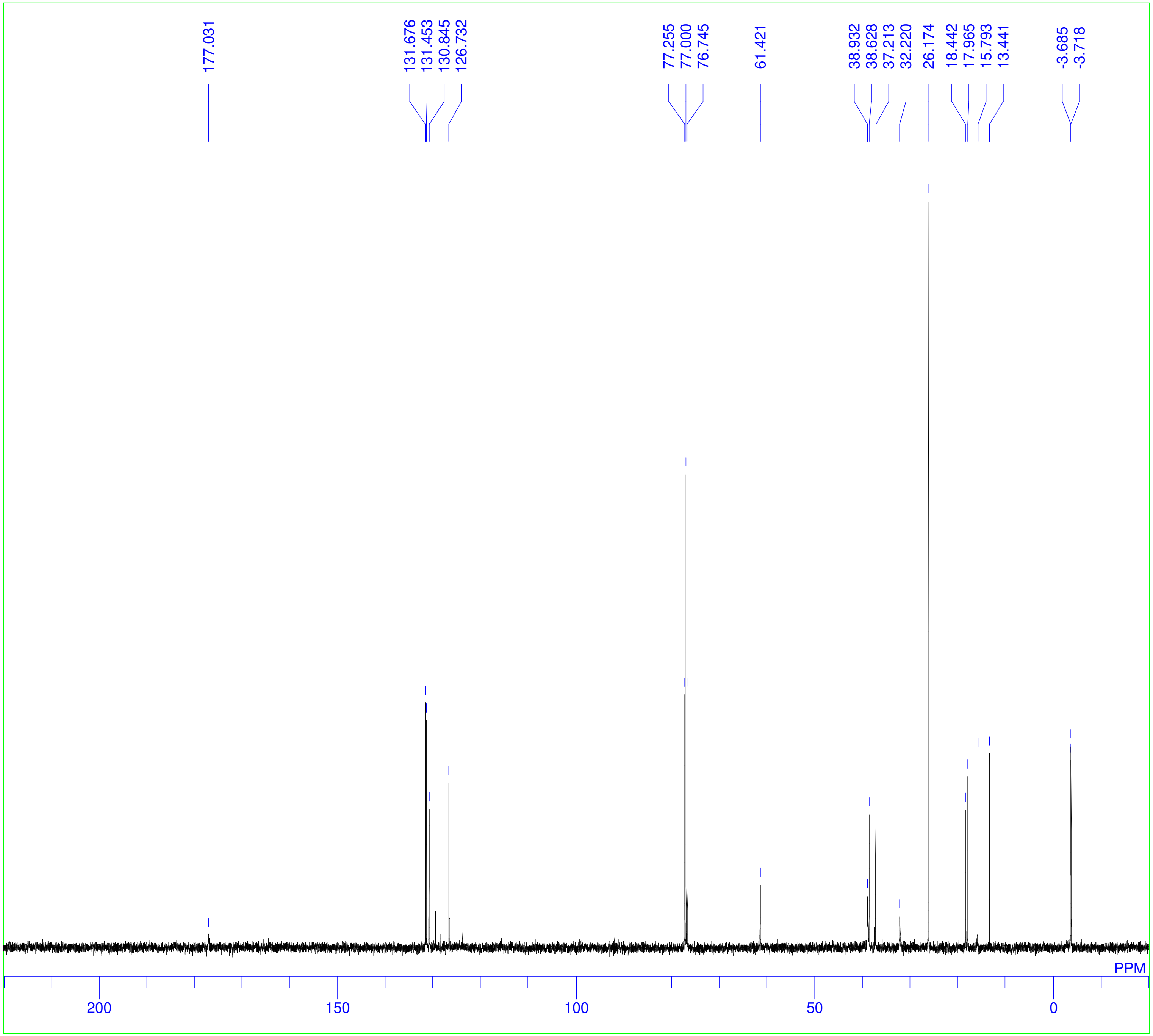

DFILE	19_13C.als
COMNT	
DATIM	Thu Jan 26 11:51:43 2012
OBNUC	13C
EXMOD	bcm
OBFRQ	125.65 MHz
OBSET	0.00 KHz
OBFIN	127958.00 Hz
POINT	32768
FREQU	33898.30 Hz
SCANS	256
ACQTM	0.9667 sec
PD	2.0333 sec
PW1	4.90 usec
IRNUC	1H
CTEMP	24.6 c
SLVNT	CDCL3
EXREF	77.00 ppm
BF	1.20 Hz
RGAIN	30

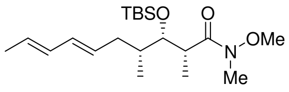

19

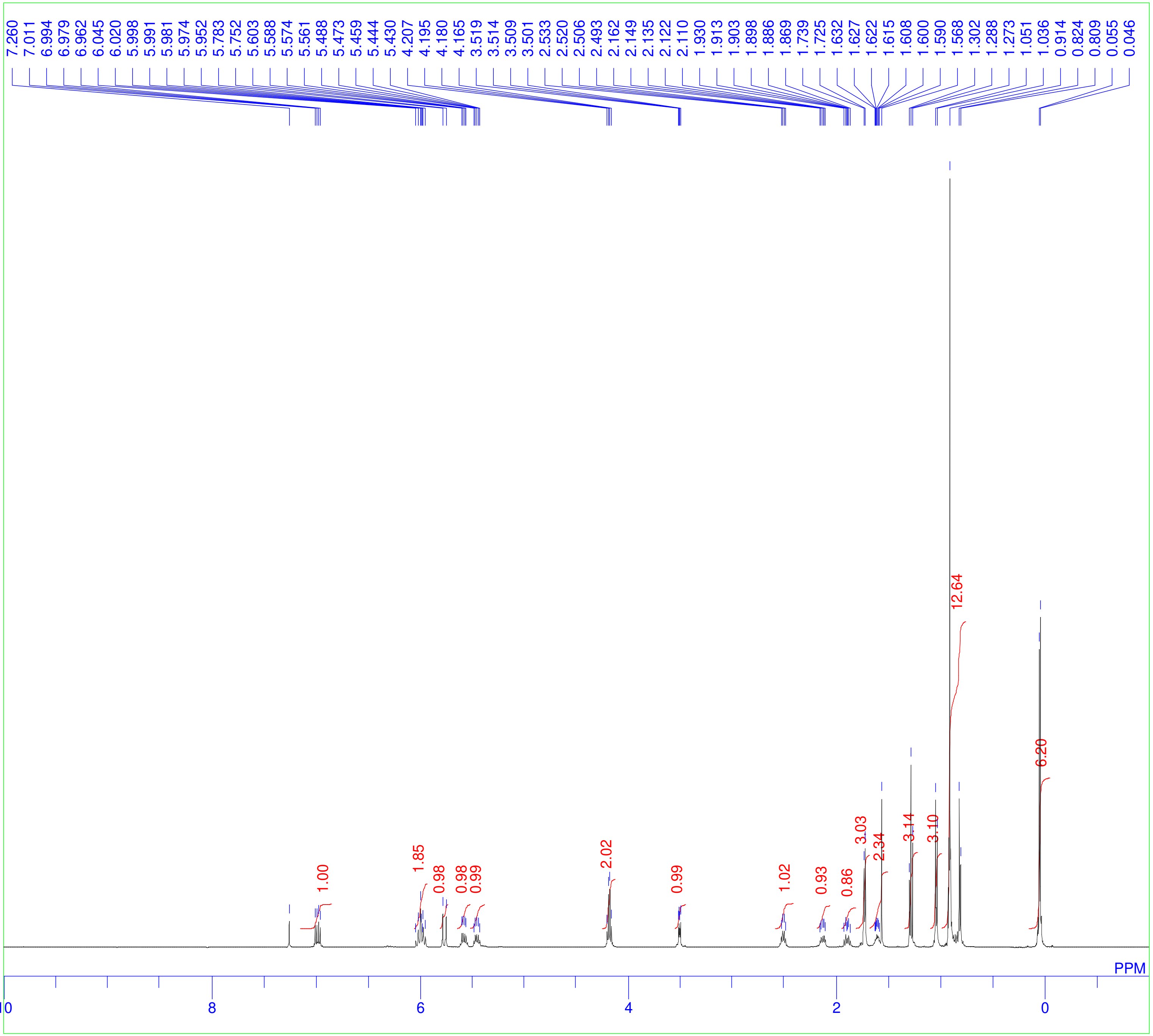

DFILE	10_1H.als
COMNT	
DATIM	Fri Jul 01 18:33:14 2011
OBNUC	1H
EXMOD	non
OBFRQ	500.00 MHz
OBSET	0.00 KHz
OBFIN	162160.00 Hz
POINT	8192
FREQU	10000.00 Hz
SCANS	8
ACQTM	0.8192 sec
PD	6.1808 sec
PW1	6.20 usec
IRNUC	1H
CTEMP	26.6 c
SLVNT	CDCL3
EXREF	7.26 ppm
BF	0.12 Hz
RGAIN	19

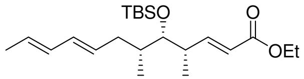

10

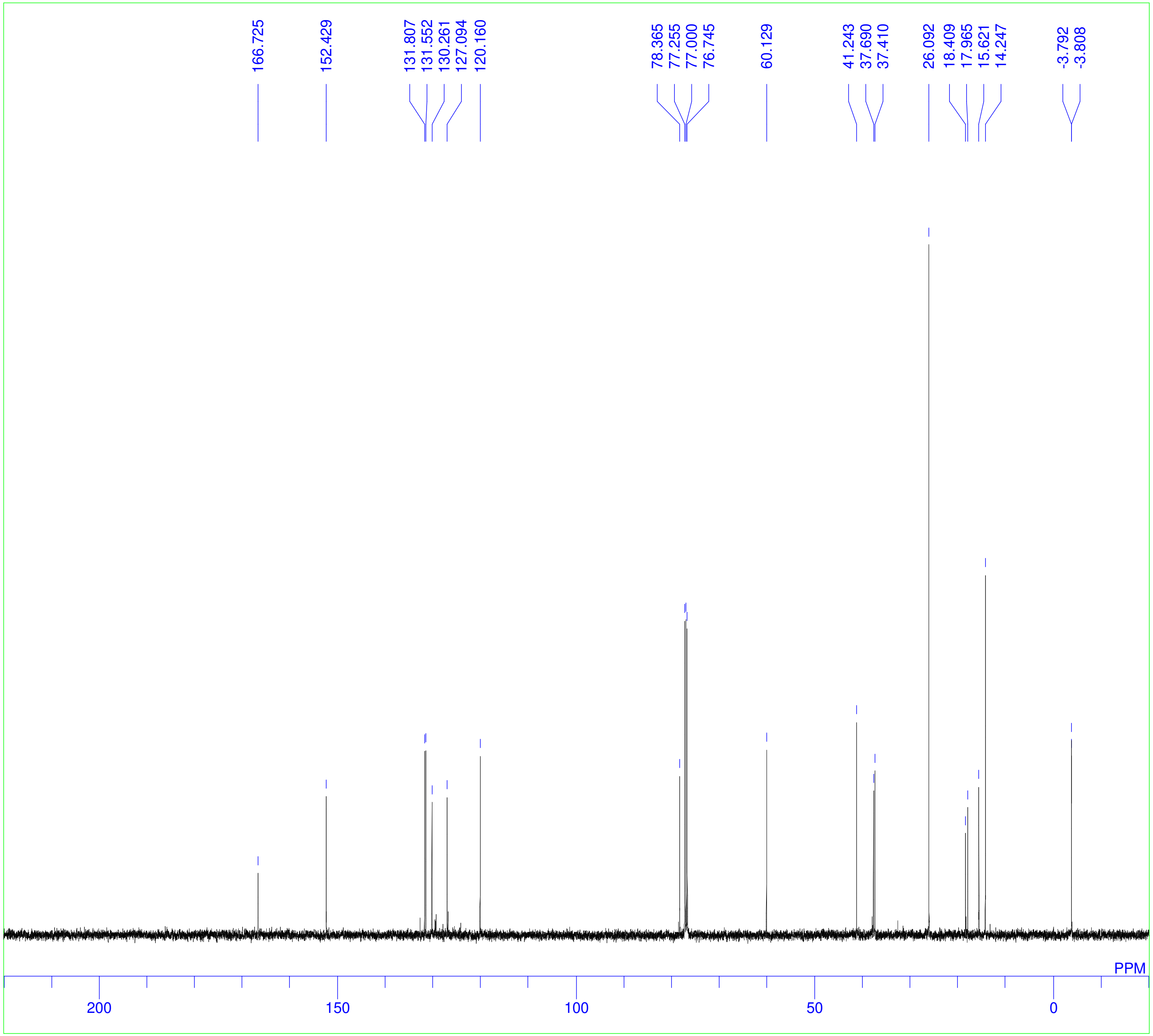

DFILE	10_13C.als
COMNT	
DATIM	Wed Sep 14 11:31:43 2011
OBNUC	13C
EXMOD	bcm
OBFRQ	125.65 MHz
OBSET	0.00 KHz
OBFIN	127958.00 Hz
POINT	32768
FREQU	33898.30 Hz
SCANS	512
ACQTM	0.9667 sec
PD	2.0333 sec
PW1	4.90 usec
IRNUC	1H
CTEMP	29.1 c
SLVNT	CDCL3
EXREF	77.00 ppm
BF	1.20 Hz
RGAIN	31

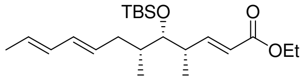

10

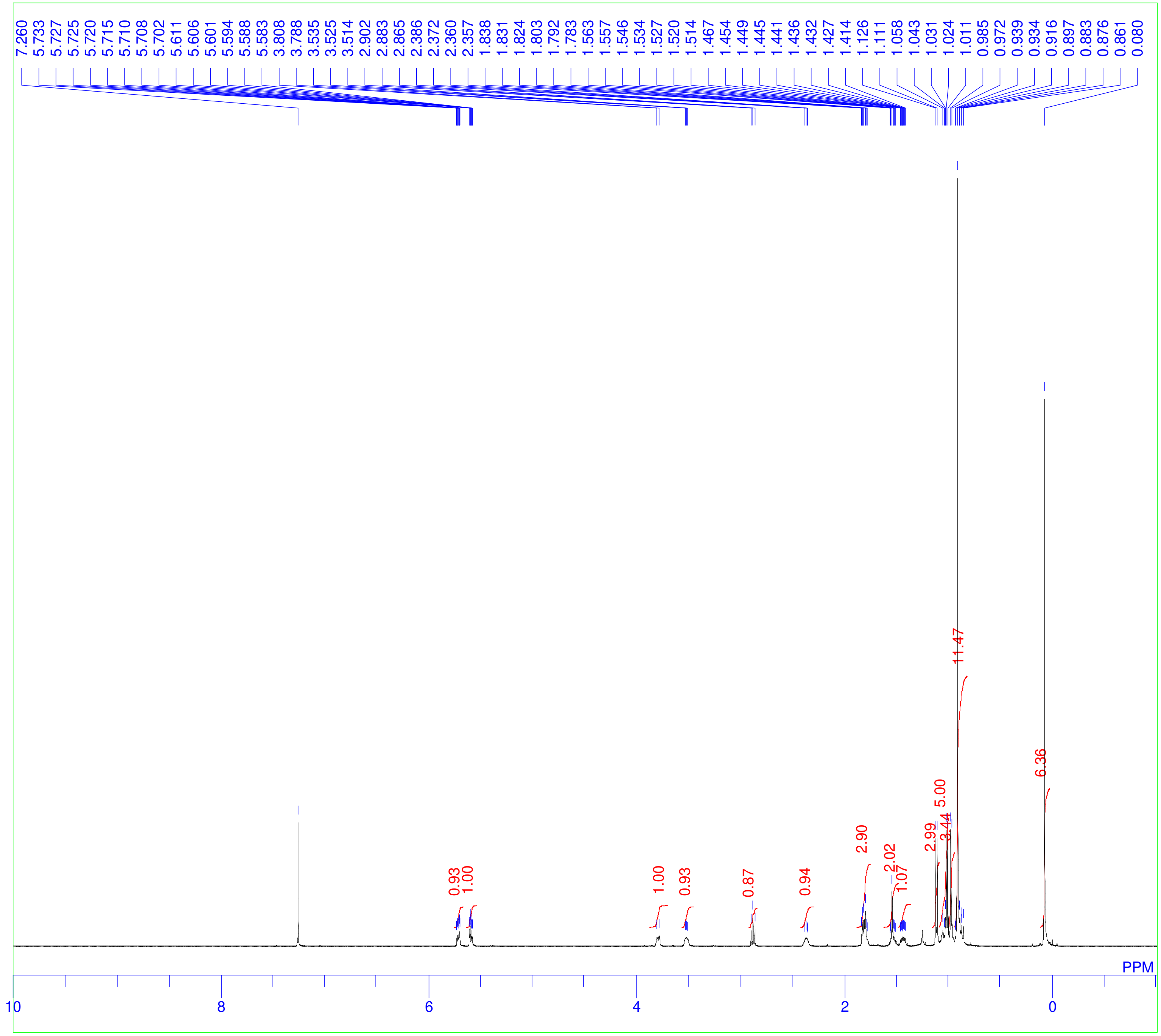

DFILE	9_1H.als
COMNT	
DATIM	Fri Jan 14 21:38:26 2011
OBNUC	1H
EXMOD	non
OBFRQ	500.00 MHz
OBSET	0.00 KHz
OBFIN	162160.00 Hz
POINT	32768
FREQU	10000.00 Hz
SCANS	8
ACQTM	3.2768 sec
PD	3.7232 sec
PW1	6.50 usec
IRNUC	1H
CTEMP	24.2 c
SLVNT	CDCL3
EXREF	7.26 ppm
BF	0.12 Hz
RGAIN	21

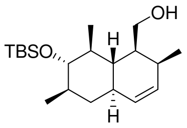

9

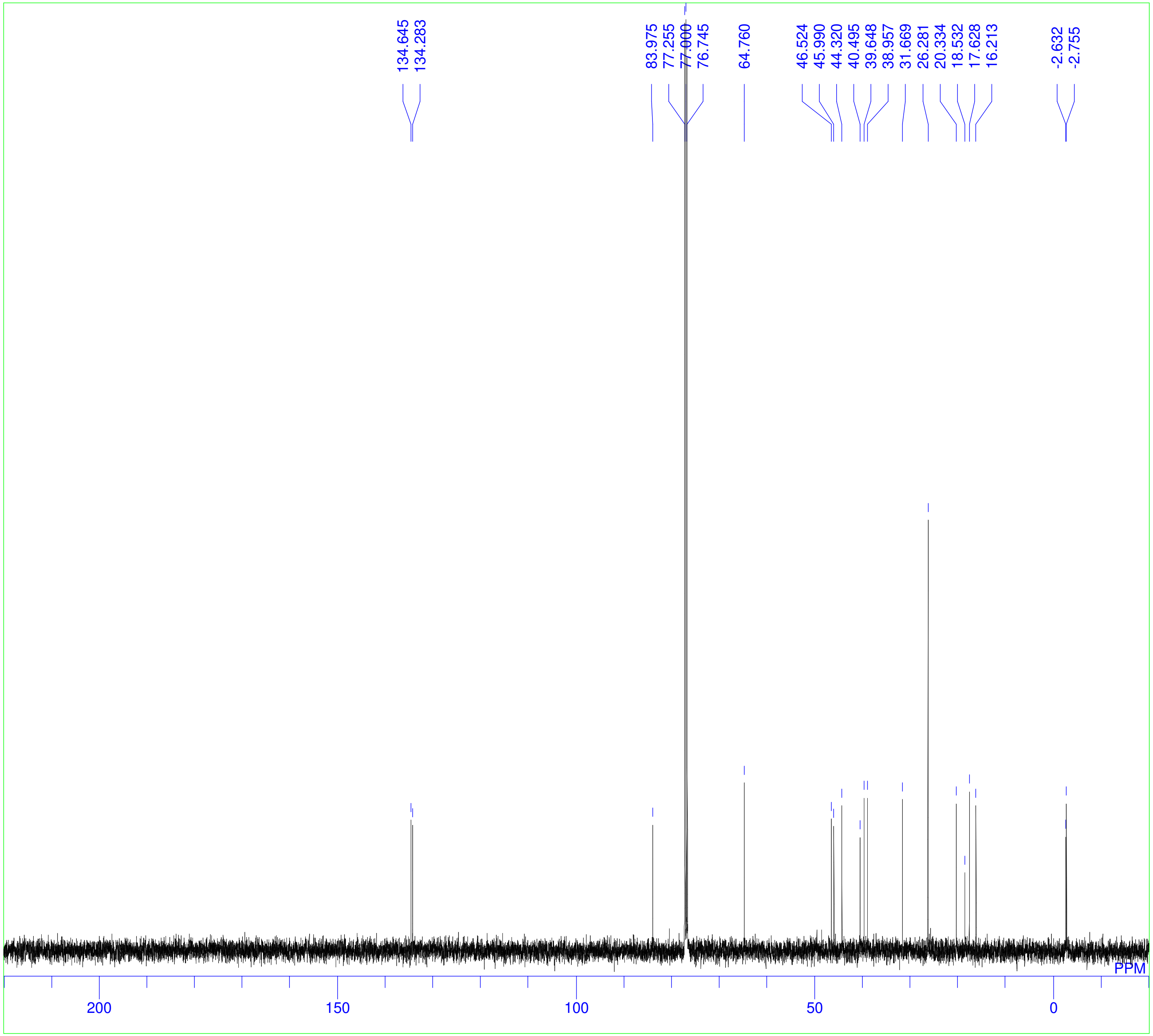

DFILE	9_13C.als
COMNT	
DATIM	Fri Jan 14 21:32:57 2011
OBNUC	13C
EXMOD	bcm
OBFRQ	125.65 MHz
OBSET	0.00 KHz
OBFIN	127958.00 Hz
POINT	32768
FREQU	33898.30 Hz
SCANS	640
ACQTM	0.9667 sec
PD	2.0333 sec
PW1	5.10 usec
IRNUC	1H
CTEMP	24.7 c
SLVNT	CDCL3
EXREF	77.00 ppm
BF	1.20 Hz
RGAIN	30

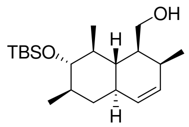

9

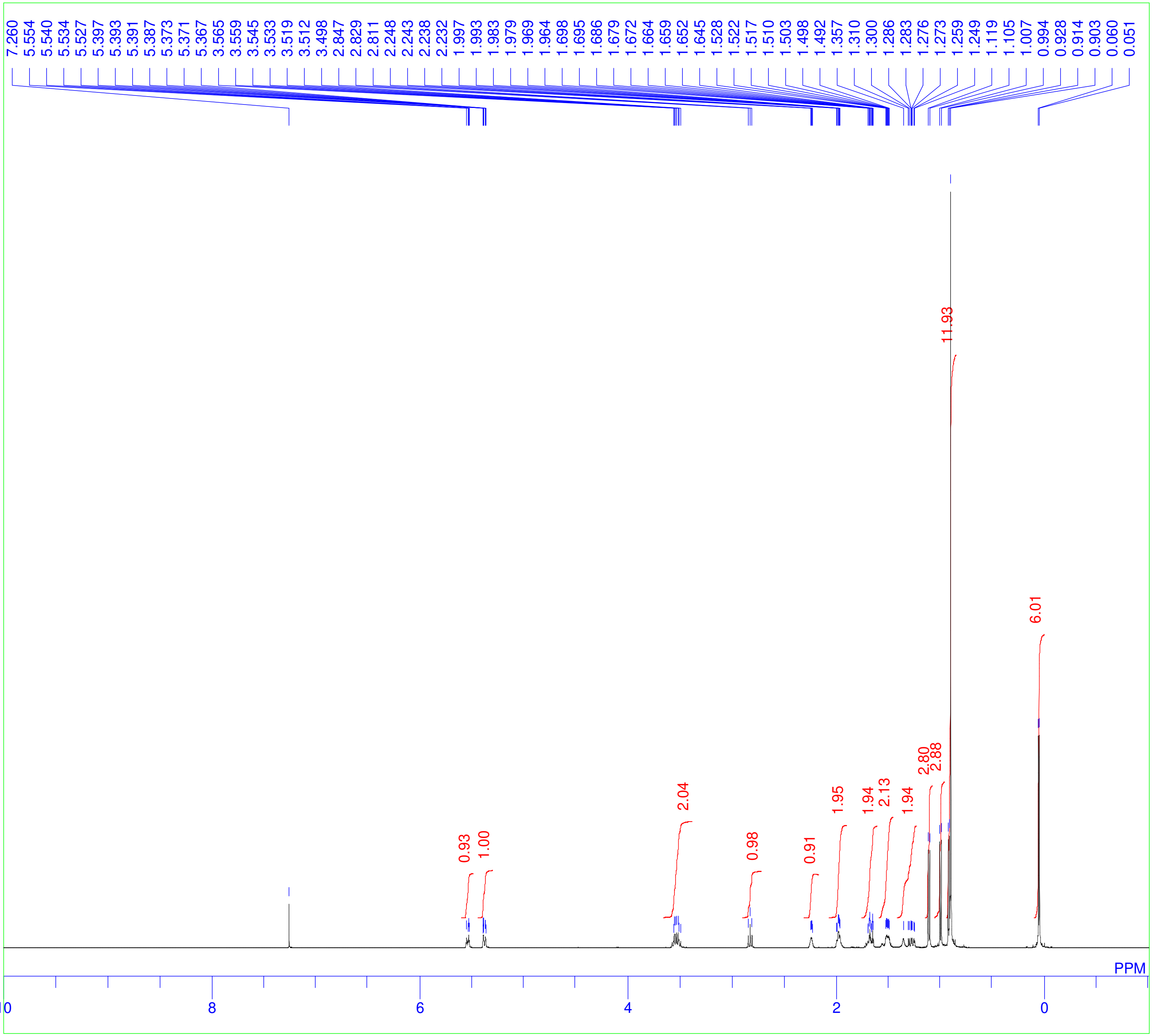

DFILE	24_1H.als
COMNT	
DATIM	Fri Jan 14 17:48:21 2011
OBNUC	1H
EXMOD	non
OBFRQ	500.00 MHz
OBSET	0.00 KHz
OBFIN	162160.00 Hz
POINT	32768
FREQU	10000.00 Hz
SCANS	8
ACQTM	3.2768 sec
PD	3.7232 sec
PW1	6.50 usec
IRNUC	1H
CTEMP	23.8 c
SLVNT	CDCL3
EXREF	7.26 ppm
BF	0.12 Hz
RGAIN	17

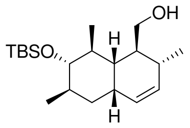

24

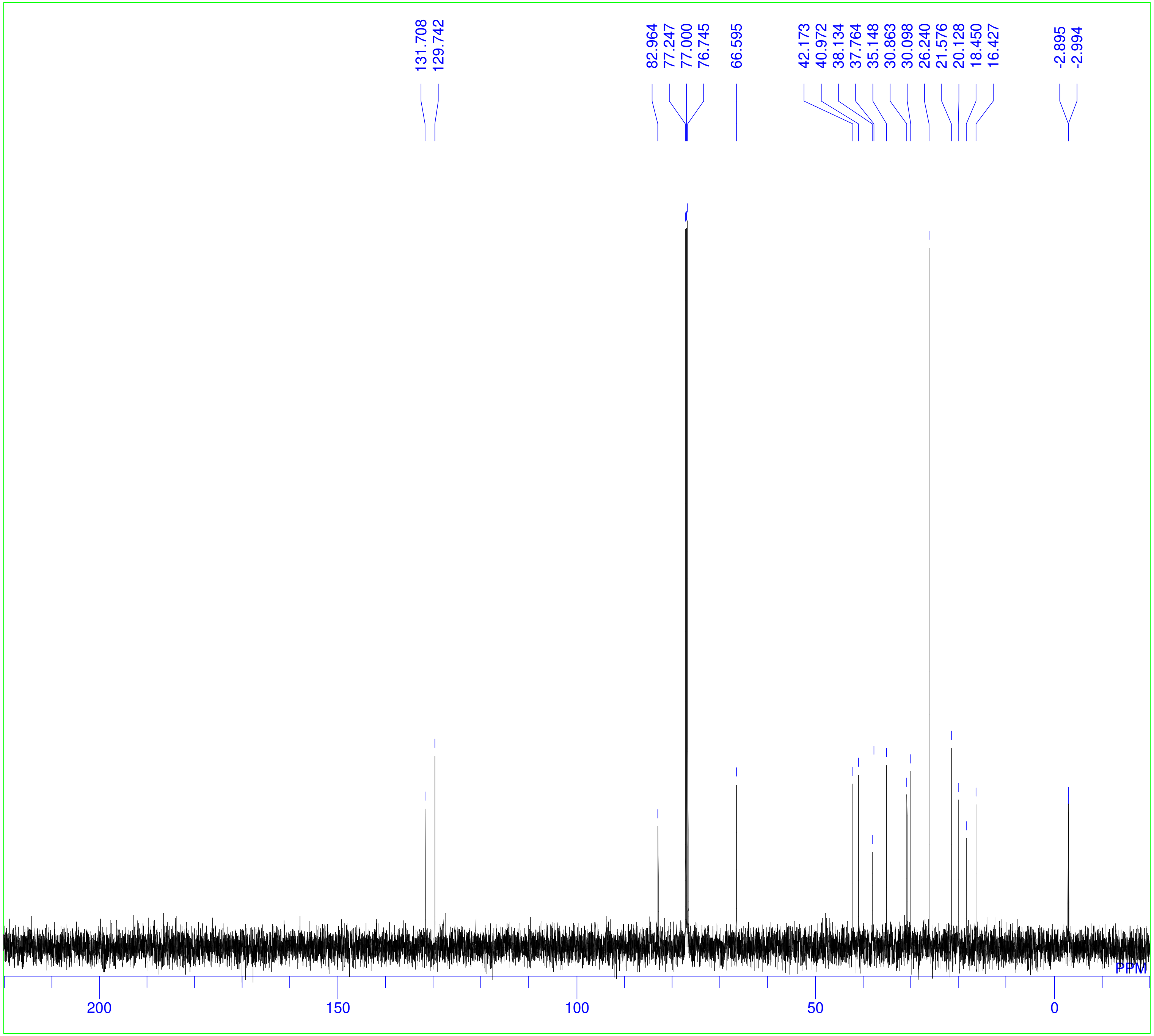

DFILE	24_13C.als
COMNT	
DATIM	Fri Jan 14 17:56:40 2011
OBNUC	13C
EXMOD	bcm
OBFRQ	125.65 MHz
OBSET	0.00 KHz
OBFIN	127958.00 Hz
POINT	32768
FREQU	33898.30 Hz
SCANS	160
ACQTM	0.9667 sec
PD	2.0333 sec
PW1	5.10 usec
IRNUC	1H
CTEMP	24.9 c
SLVNT	CDCL3
EXREF	77.00 ppm
BF	1.20 Hz
RGAIN	30

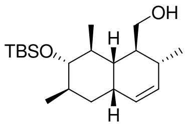

24

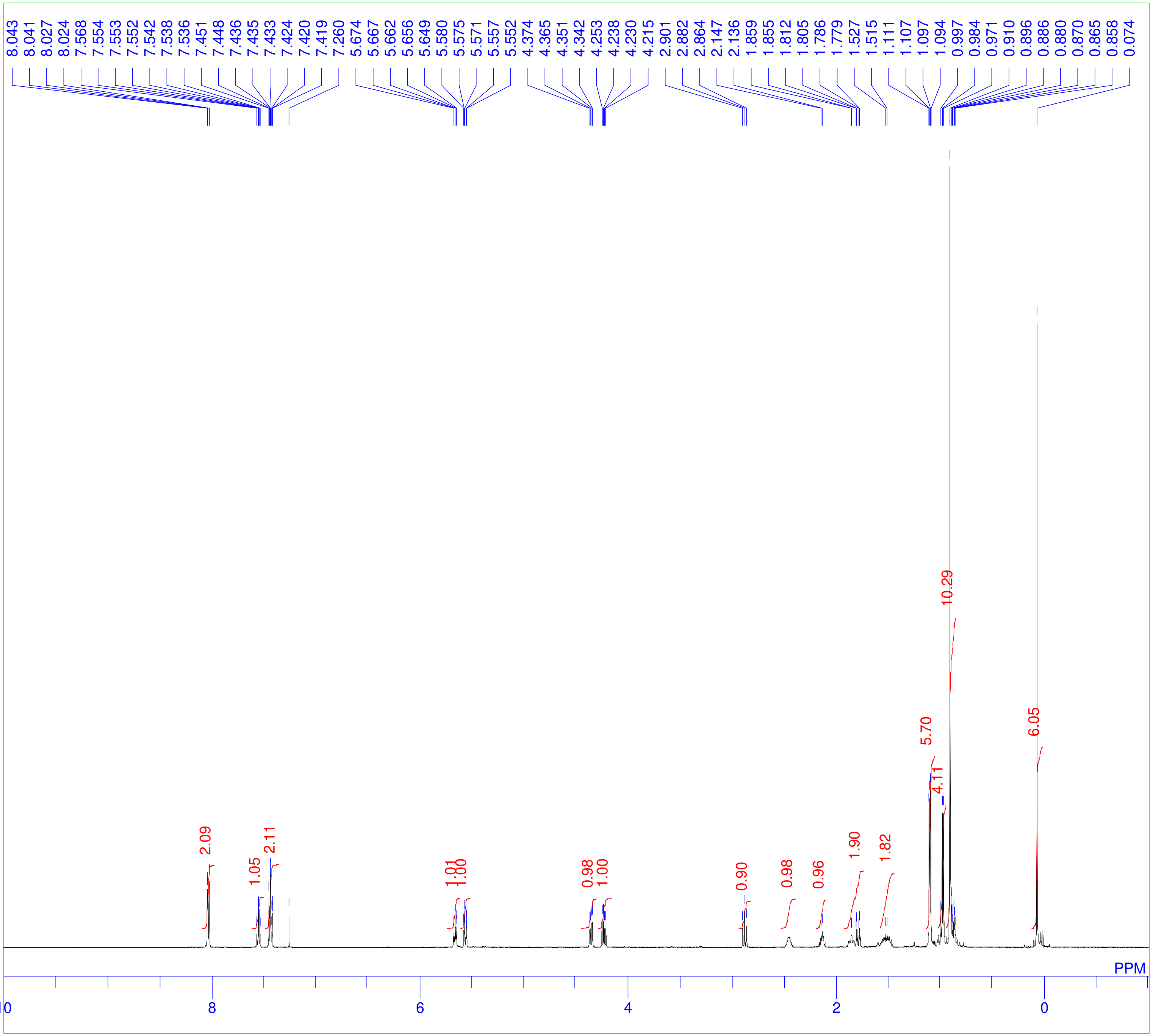

DFILE	25_1H-CDCl3.als
COMNT	
DATIM	2018-02-17 11:51:28
OBNUC	1H
EXMOD	proton.jxp
OBFRQ	500.16 MHz
OBSET	2.41 KHz
OBFIN	6.01 Hz
POINT	13107
FREQU	7507.51 Hz
SCANS	8
ACQTM	1.7459 sec
PD	5.0000 sec
PW1	3.35 usec
IRNUC	1H
CTEMP	19.2 c
SLVNT	CDCL3
EXREF	7.26 ppm
BF	0.30 Hz
RGAIN	24

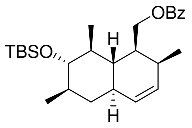

25

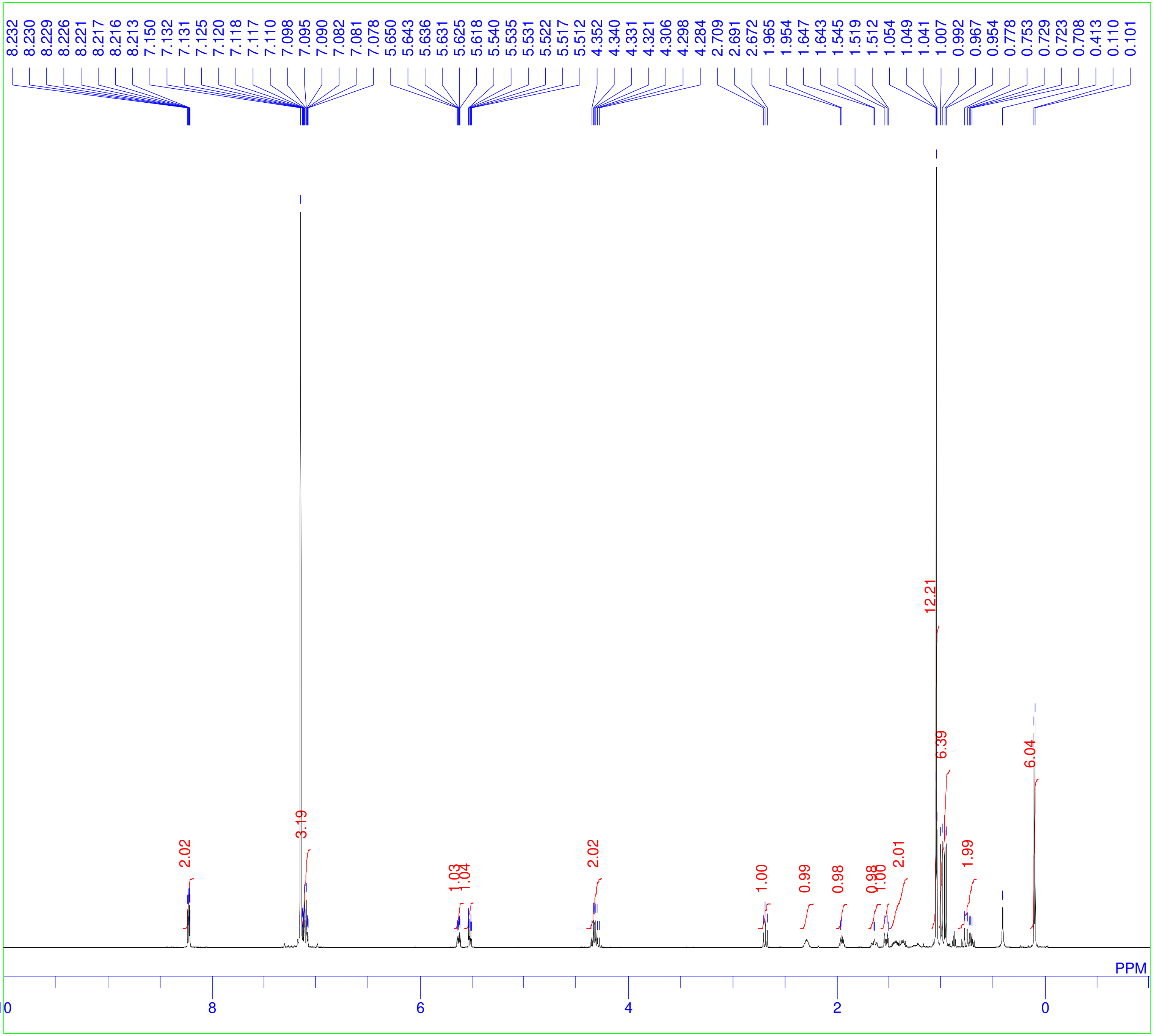

DFILE	25_1H-C6D6.als
COMNT	
DATIM	2022-09-21 12:08:44
OBNUC	1H
EXMOD	proton.jxp
OBFRQ	500.16 MHz
OBSET	2.41 KHz
OBFIN	6.01 Hz
POINT	13107
FREQU	7507.51 Hz
SCANS	8
ACQTM	1.7459 sec
PD	5.0000 sec
PW1	3.84 usec
IRNUC	1H
CTEMP	23.8 c
SLVNT	C6D6
EXREF	7.15 ppm
BF	0.30 Hz
RGAIN	40

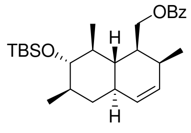

25

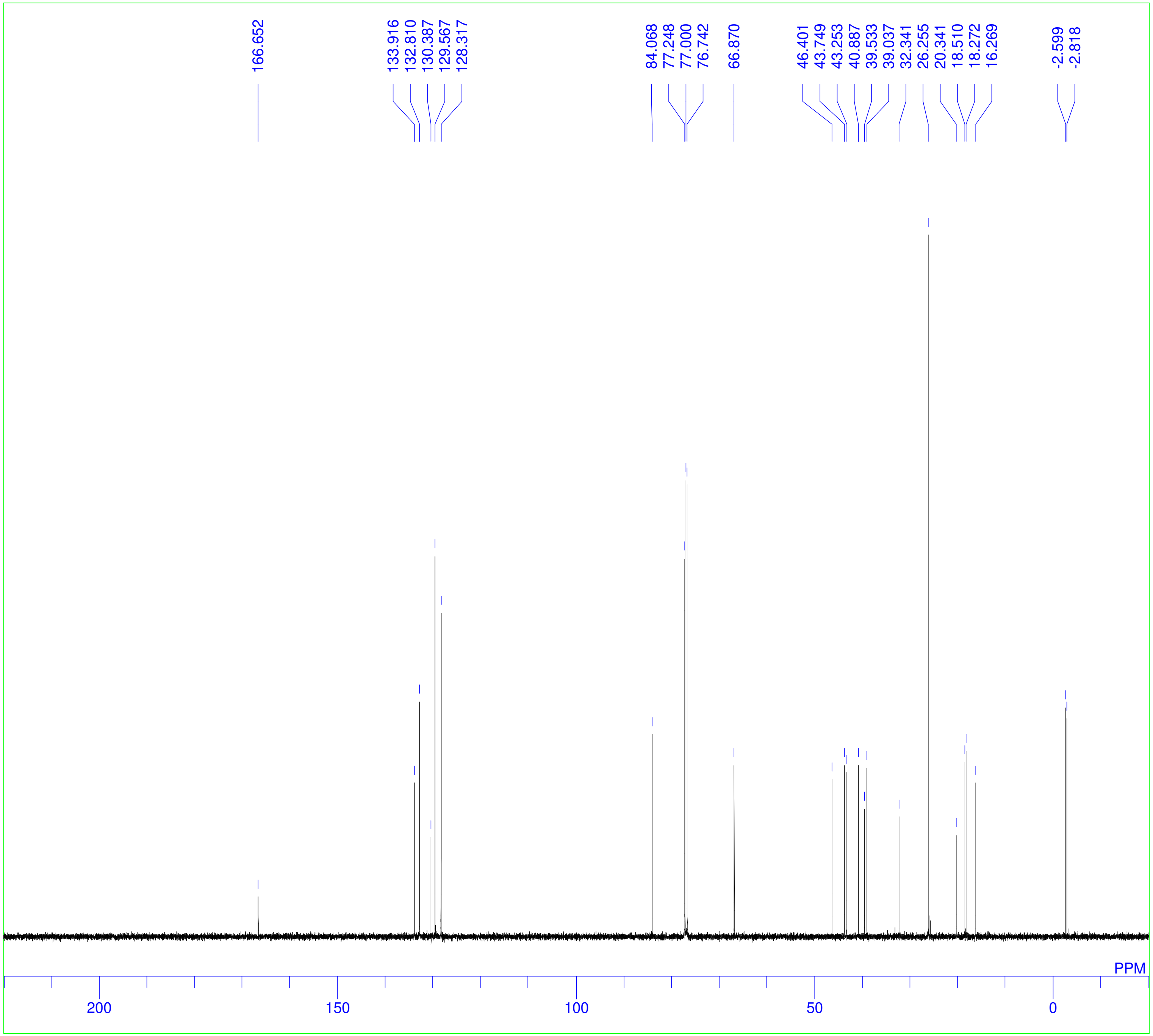

DFILE	25_13C-CDCl3.als
COMNT	
DATIM	2018-02-17 11:54:39
OBNUC	13C
EXMOD	carbon.jxp
OBFRQ	125.77 MHz
OBSET	7.87 KHz
OBFIN	4.21 Hz
POINT	26214
FREQU	31446.54 Hz
SCANS	512
ACQTM	0.8336 sec
PD	2.0000 sec
PW1	4.00 usec
IRNUC	1H
CTEMP	19.4 c
SLVNT	CDCL3
EXREF	77.00 ppm
BF	0.30 Hz
RGAIN	22

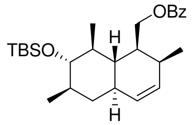

25

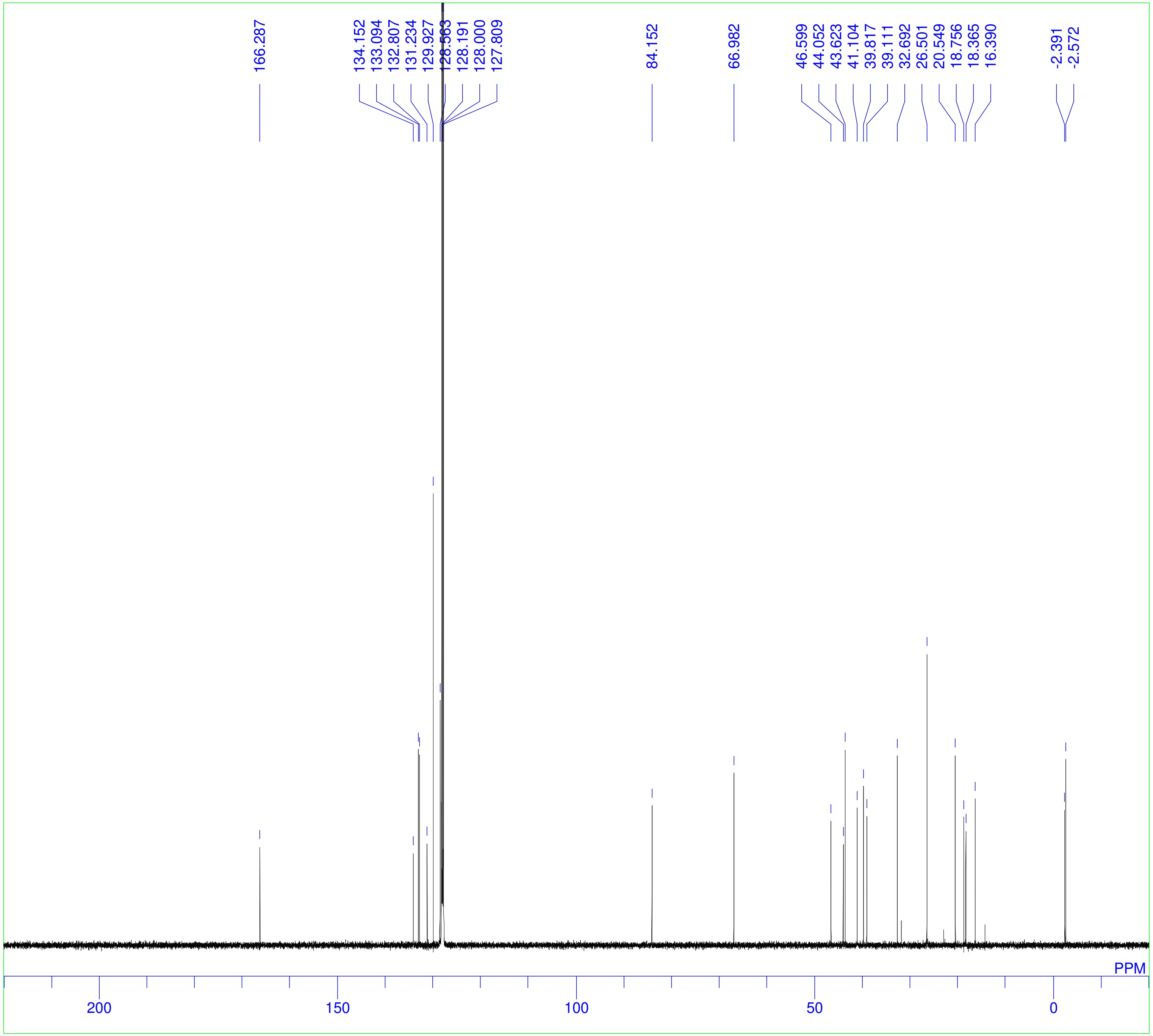

DFILE	25_13C-C6D6.als
COMNT	
DATIM	2022-09-21 12:14:07
OBNUC	13C
EXMOD	carbon.jxp
OBFRQ	125.77 MHz
OBSET	7.87 KHz
OBFIN	4.21 Hz
POINT	26214
FREQU	31446.54 Hz
SCANS	1024
ACQTM	0.8336 sec
PD	2.0000 sec
PW1	3.87 usec
IRNUC	1H
CTEMP	24.0 c
SLVNT	C6D6
EXREF	128.00 ppm
BF	0.30 Hz
RGAIN	34

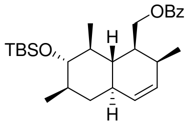

25

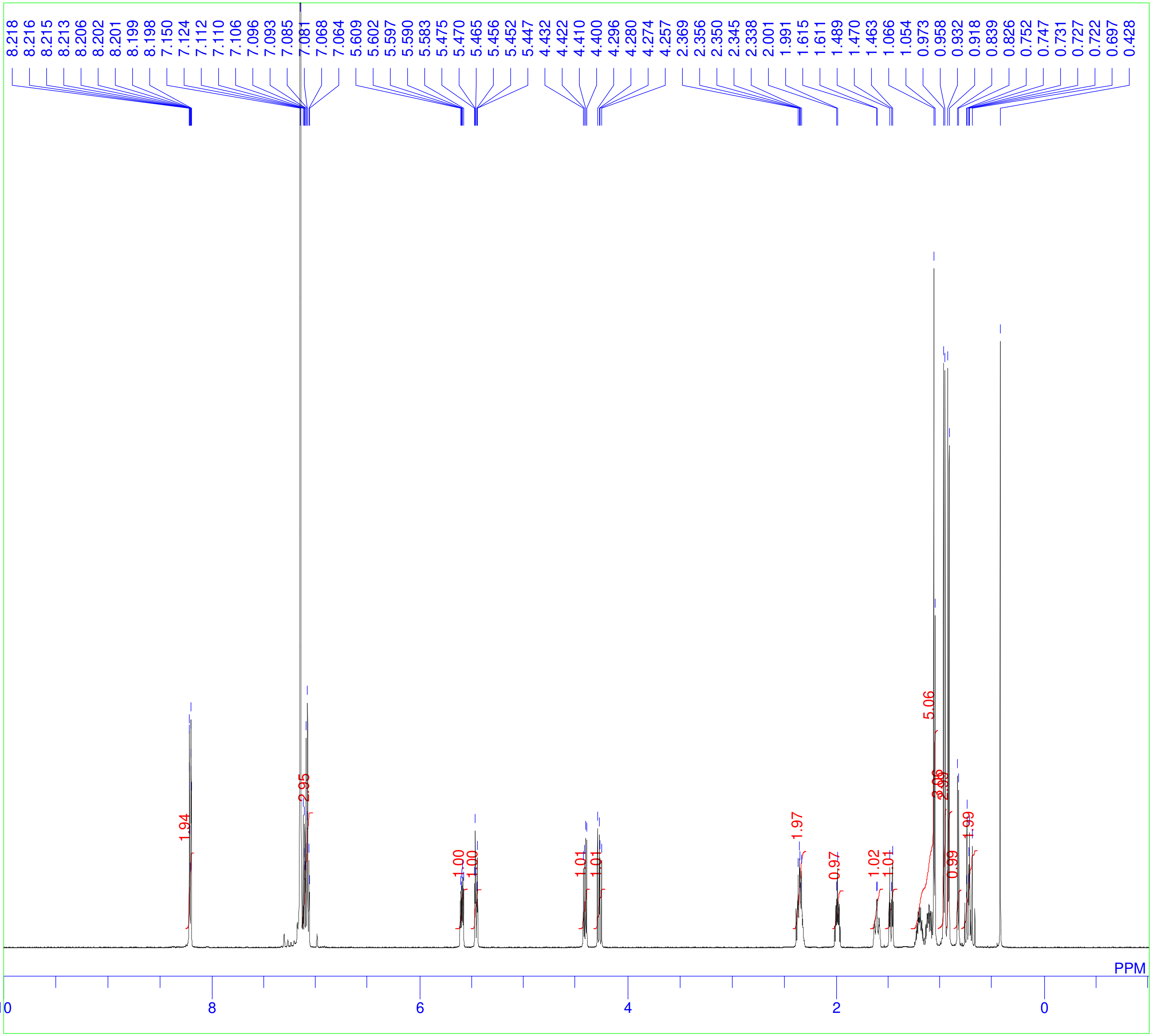

DFILE	26_1H.als
COMNT	
DATIM	2022-09-21 12:03:05
OBNUC	1H
EXMOD	proton.jxp
OBFRQ	500.16 MHz
OBSET	2.41 KHz
OBFIN	6.01 Hz
POINT	13107
FREQU	7507.51 Hz
SCANS	8
ACQTM	1.7459 sec
PD	5.0000 sec
PW1	3.84 usec
IRNUC	1H
CTEMP	24.0 c
SLVNT	C6D6
EXREF	7.15 ppm
BF	0.30 Hz
RGAIN	40

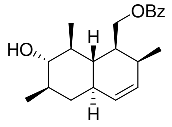

26

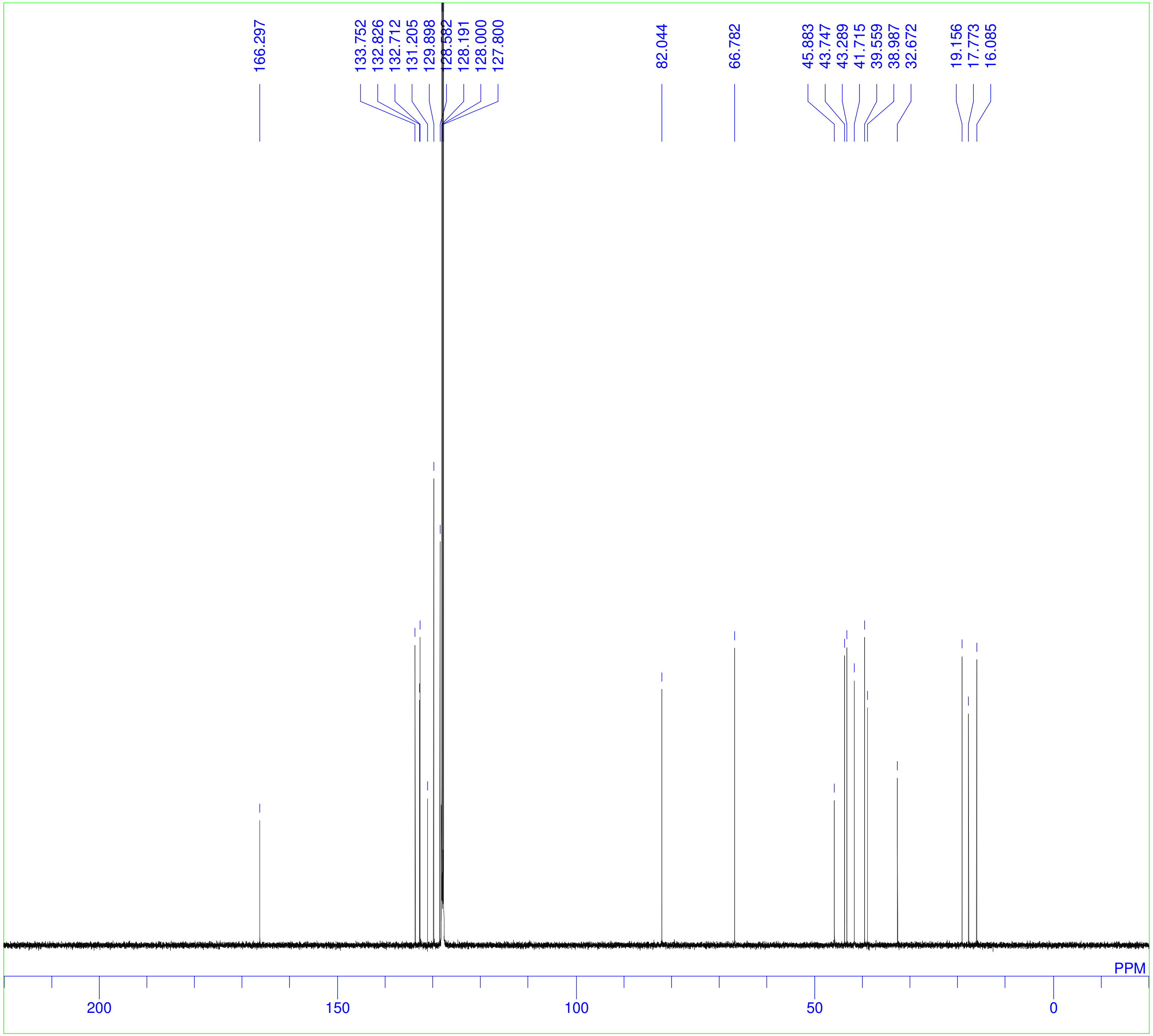

DFILE	26_13C.als
COMNT	
DATIM	2022-09-21 10:37:02
OBNUC	13C
EXMOD	carbon.jxp
OBFRQ	125.77 MHz
OBSET	7.87 KHz
OBFIN	4.21 Hz
POINT	26214
FREQU	31446.54 Hz
SCANS	1024
ACQTM	0.8336 sec
PD	2.0000 sec
PW1	3.87 usec
IRNUC	1H
CTEMP	24.1 c
SLVNT	C6D6
EXREF	128.00 ppm
BF	0.30 Hz
RGAIN	30

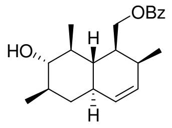

26

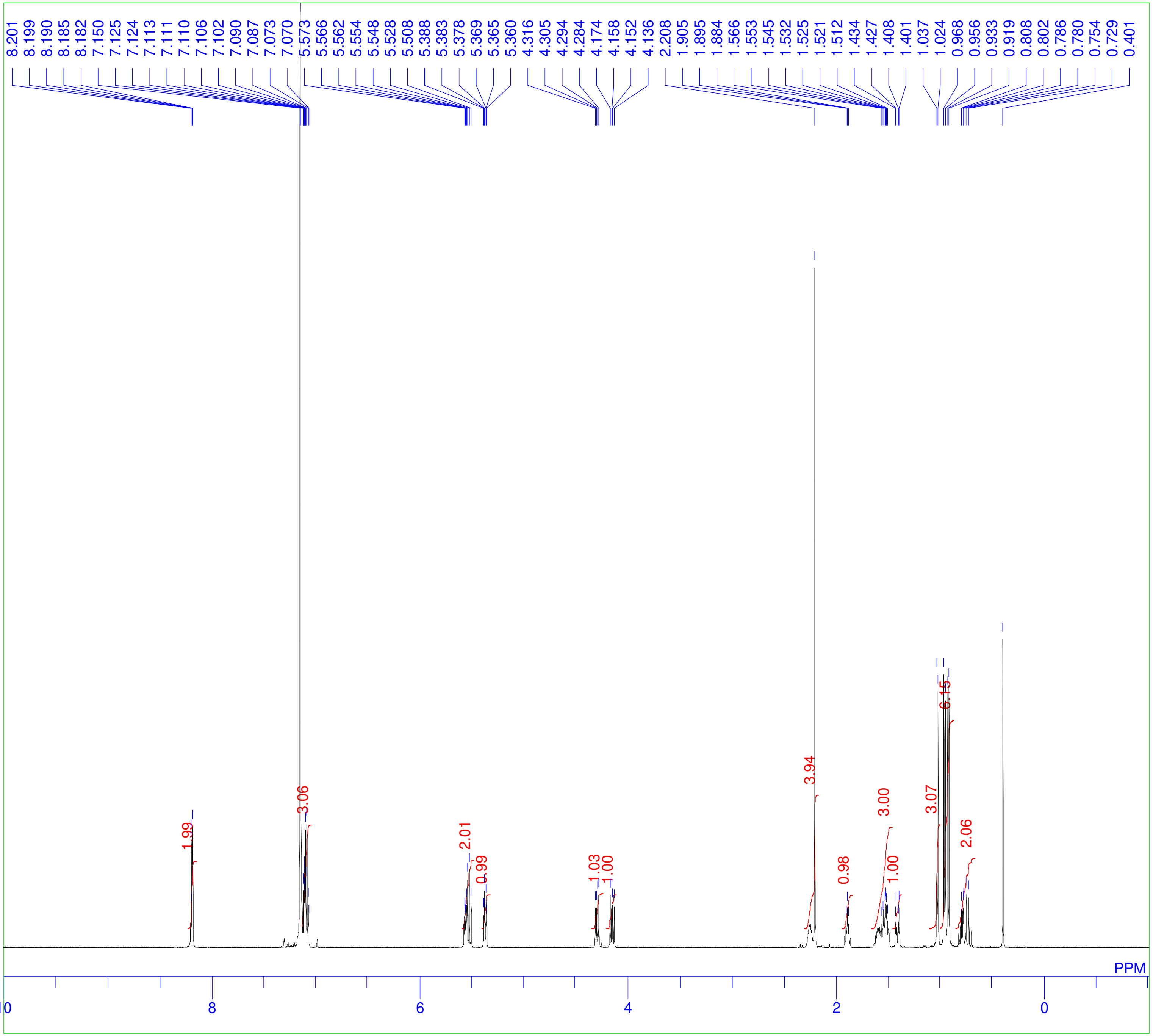

DFILE	8_1H.als
COMNT	
DATIM	2022-09-22 11:23:57
OBNUC	1H
EXMOD	proton.jxp
OBFRQ	500.16 MHz
OBSET	2.41 KHz
OBFIN	6.01 Hz
POINT	13107
FREQU	7507.51 Hz
SCANS	8
ACQTM	1.7459 sec
PD	5.0000 sec
PW1	3.84 usec
IRNUC	1H
CTEMP	23.9 c
SLVNT	C6D6
EXREF	7.15 ppm
BF	0.30 Hz
RGAIN	40

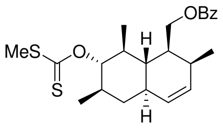

8

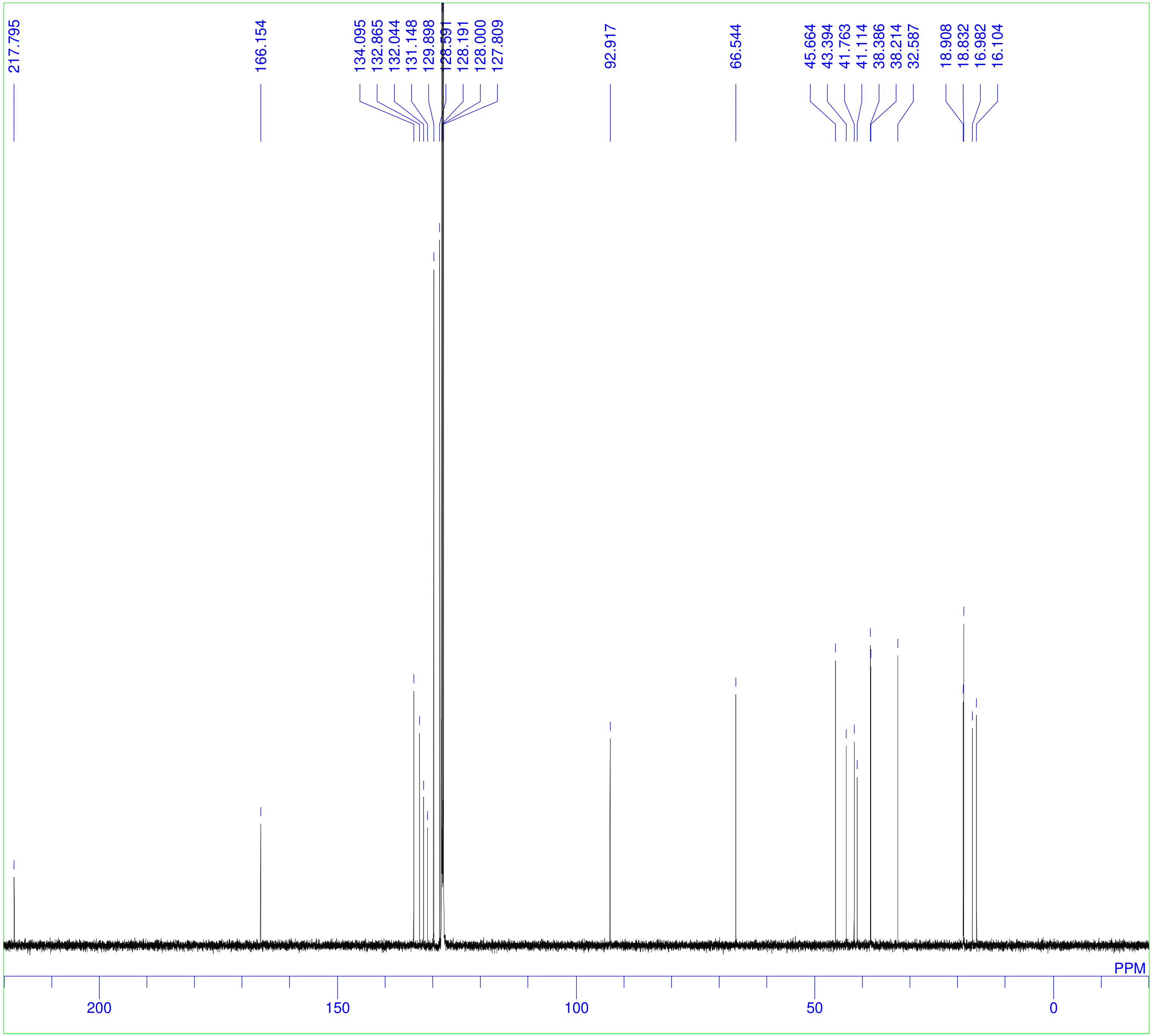

DFILE	8_13C.als
COMNT	
DATIM	2022-09-22 11:29:39
OBNUC	13C
EXMOD	carbon.jxp
OBFRQ	125.77 MHz
OBSET	7.87 KHz
OBFIN	4.21 Hz
POINT	26214
FREQU	31446.54 Hz
SCANS	1024
ACQTM	0.8336 sec
PD	2.0000 sec
PW1	3.87 usec
IRNUC	1H
CTEMP	24.2 c
SLVNT	C6D6
EXREF	128.00 ppm
BF	0.30 Hz
RGAIN	28

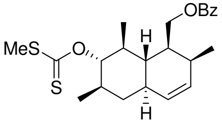

8

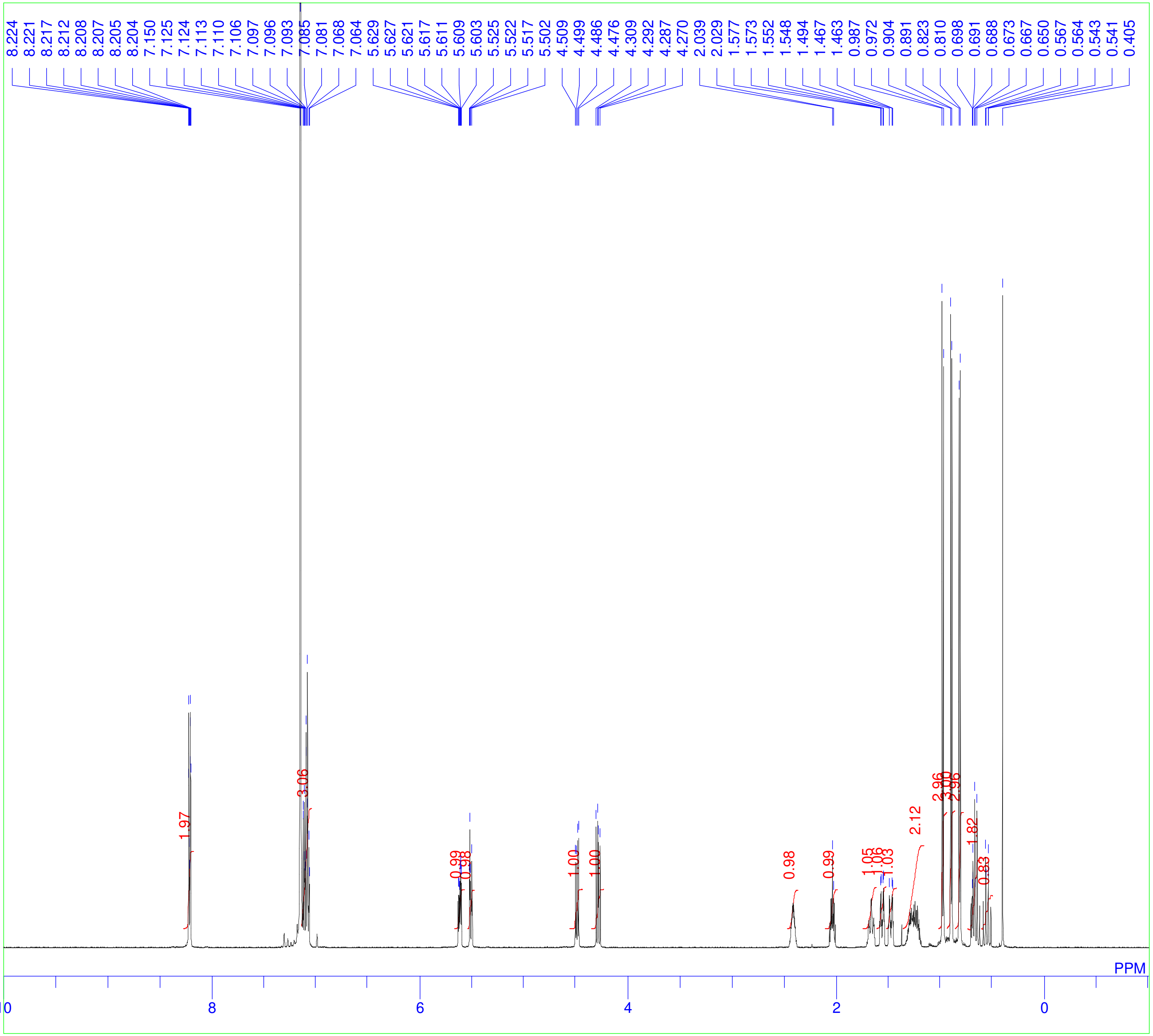

DFILE	7_1H.als
COMNT	
DATIM	2022-09-11 13:40:41
OBNUC	1H
EXMOD	proton.jxp
OBFRQ	500.16 MHz
OBSET	2.41 KHz
OBFIN	6.01 Hz
POINT	13107
FREQU	7507.51 Hz
SCANS	8
ACQTM	1.7459 sec
PD	5.0000 sec
PW1	3.84 usec
IRNUC	1H
CTEMP	24.0 c
SLVNT	C6D6
EXREF	7.15 ppm
BF	0.30 Hz
RGAIN	40

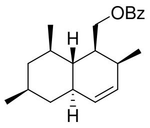

7

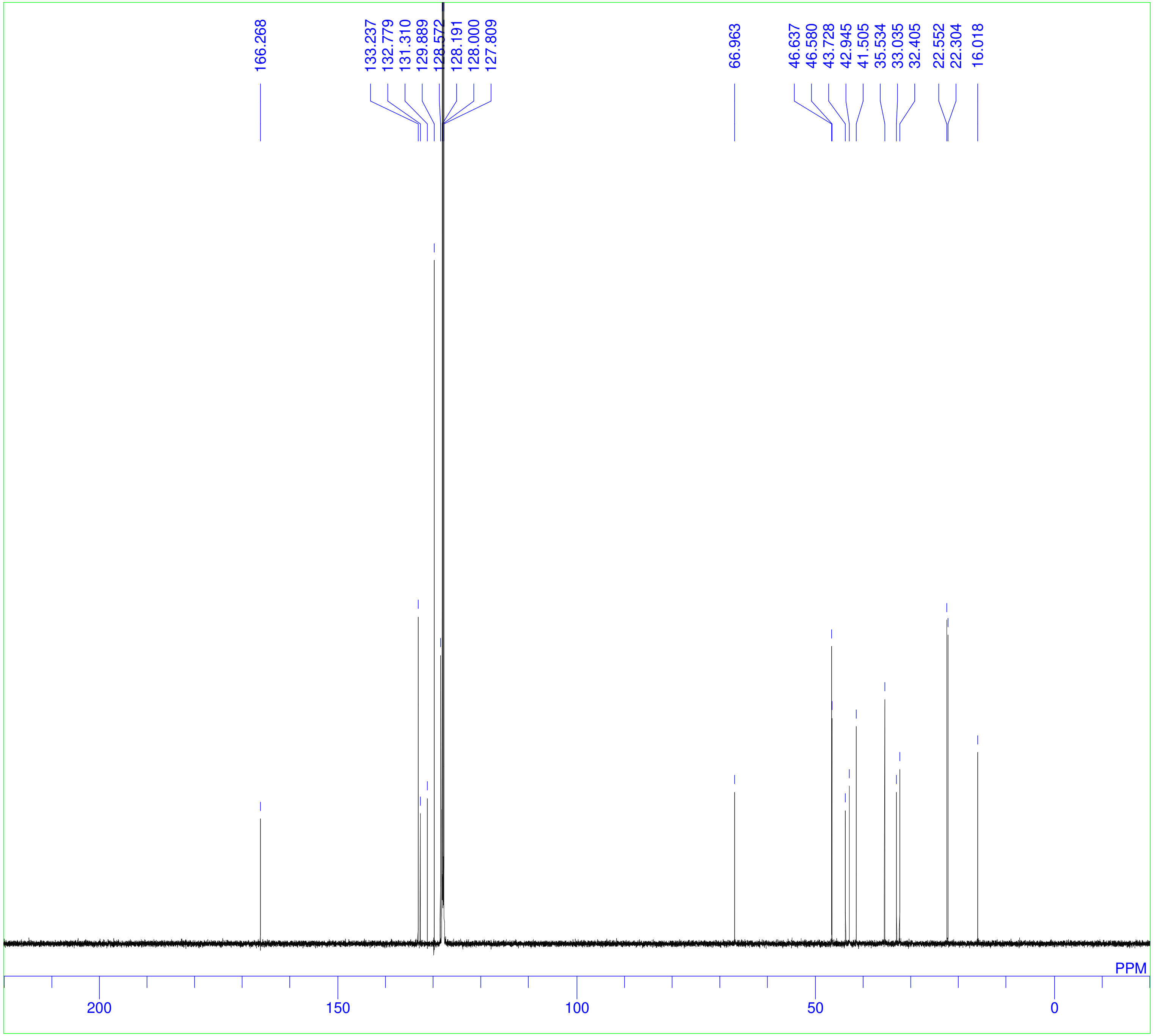

DFILE	7_13C.als
COMNT	
DATIM	2022-09-11 13:46:15
OBNUC	13C
EXMOD	carbon.jxp
OBFRQ	125.77 MHz
OBSET	7.87 KHz
OBFIN	4.21 Hz
POINT	26214
FREQU	31446.54 Hz
SCANS	1024
ACQTM	0.8336 sec
PD	2.0000 sec
PW1	3.87 usec
IRNUC	1H
CTEMP	23.8 c
SLVNT	C6D6
EXREF	128.00 ppm
BF	0.30 Hz
RGAIN	26

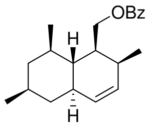

7

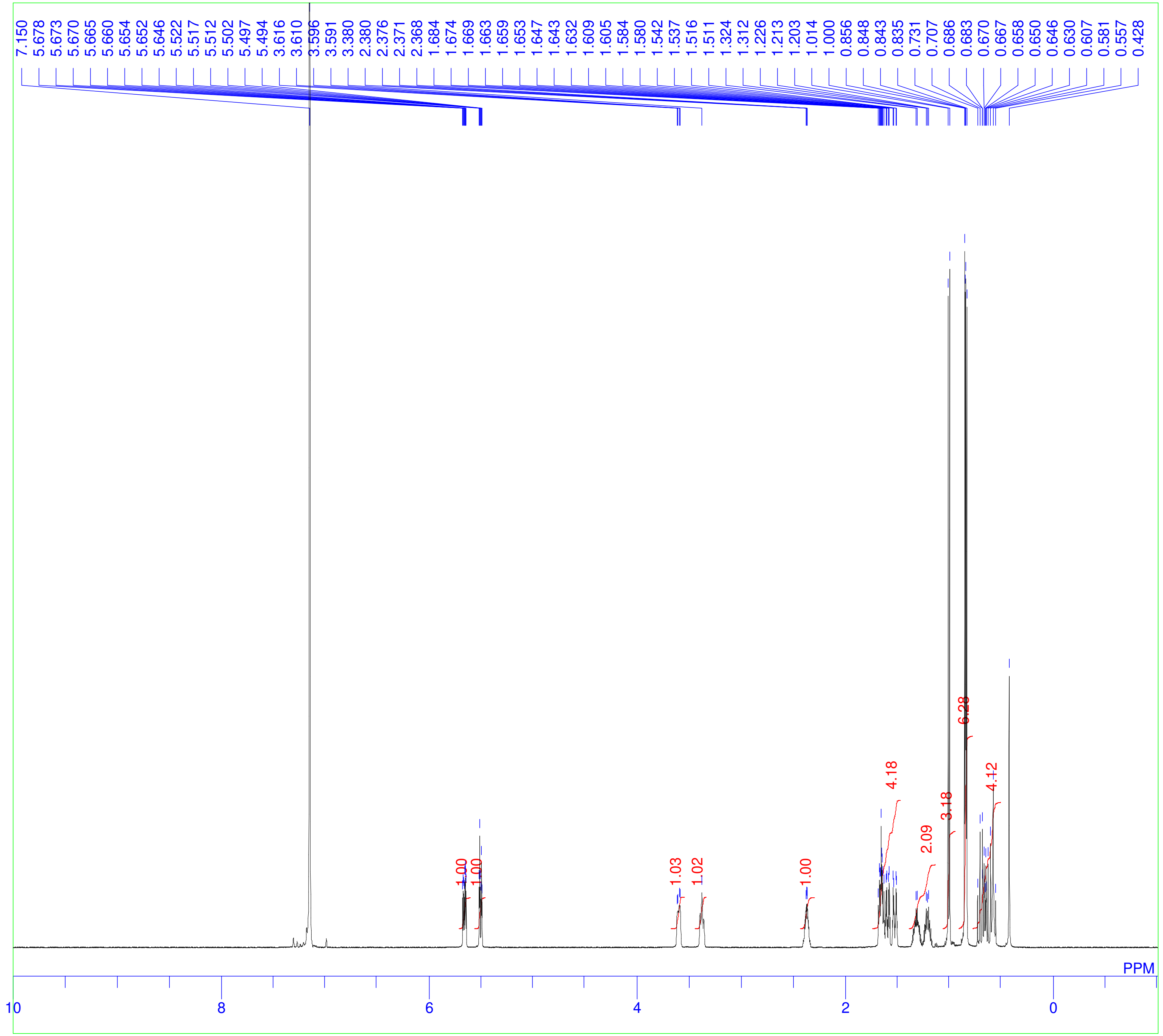

DFILE	27_1H.als
COMNT	
DATIM	2022-09-11 14:59:16
OBNUC	1H
EXMOD	proton.jxp
OBFRQ	500.16 MHz
OBSET	2.41 KHz
OBFIN	6.01 Hz
POINT	13107
FREQU	7507.51 Hz
SCANS	8
ACQTM	1.7459 sec
PD	5.0000 sec
PW1	3.84 usec
IRNUC	1H
CTEMP	24.0 c
SLVNT	C6D6
EXREF	7.15 ppm
BF	0.30 Hz
RGAIN	38

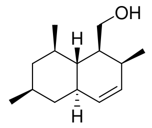

27

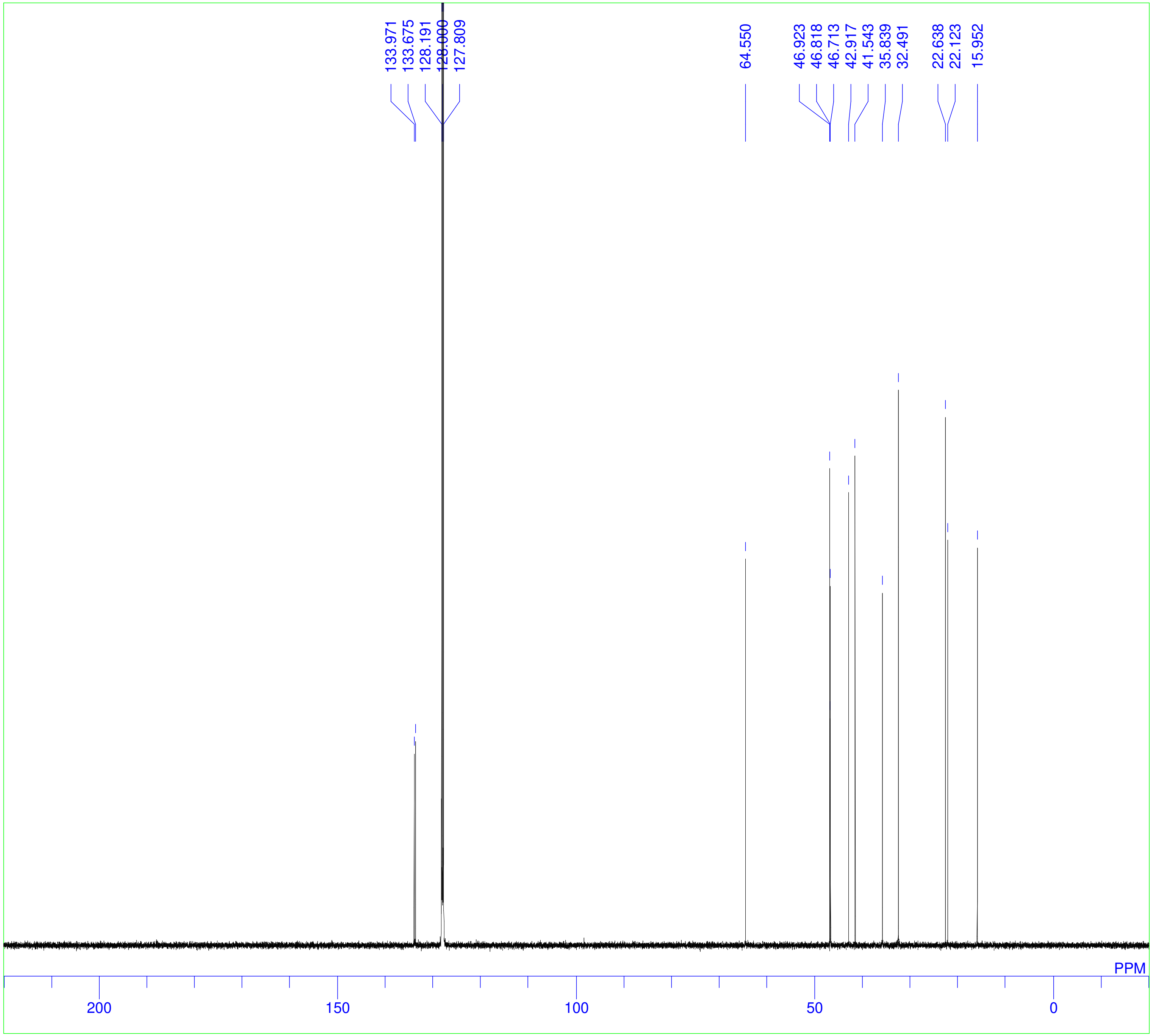

DFILE	27_13C.als
COMNT	
DATIM	2022-09-11 15:05:50
OBNUC	13C
EXMOD	carbon.jxp
OBFRQ	125.77 MHz
OBSET	7.87 KHz
OBFIN	4.21 Hz
POINT	26214
FREQU	31446.54 Hz
SCANS	1024
ACQTM	0.8336 sec
PD	2.0000 sec
PW1	3.87 usec
IRNUC	1H
CTEMP	24.2 c
SLVNT	C6D6
EXREF	128.00 ppm
BF	0.30 Hz
RGAIN	30

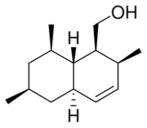

27

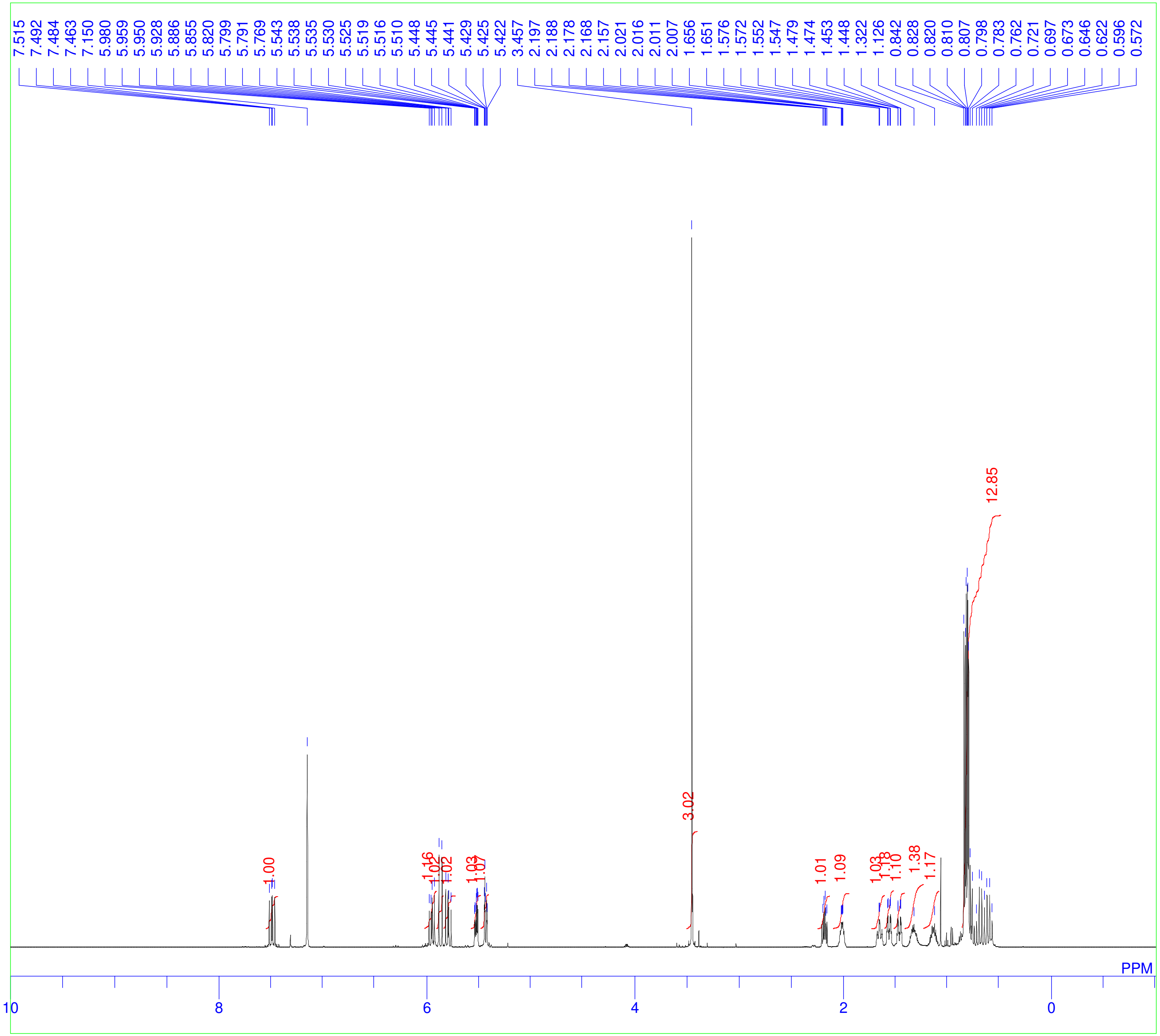

DFILE	5_1H.als
COMNT	
DATIM	2022-09-13 18:37:09
OBNUC	1H
EXMOD	proton.jxp
OBFRQ	500.16 MHz
OBSET	2.41 KHz
OBFIN	6.01 Hz
POINT	13107
FREQU	7507.51 Hz
SCANS	8
ACQTM	1.7459 sec
PD	5.0000 sec
PW1	3.84 usec
IRNUC	1H
CTEMP	24.3 c
SLVNT	C6D6
EXREF	7.15 ppm
BF	0.30 Hz
RGAIN	26

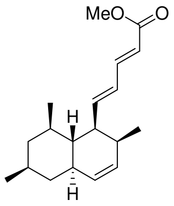

5

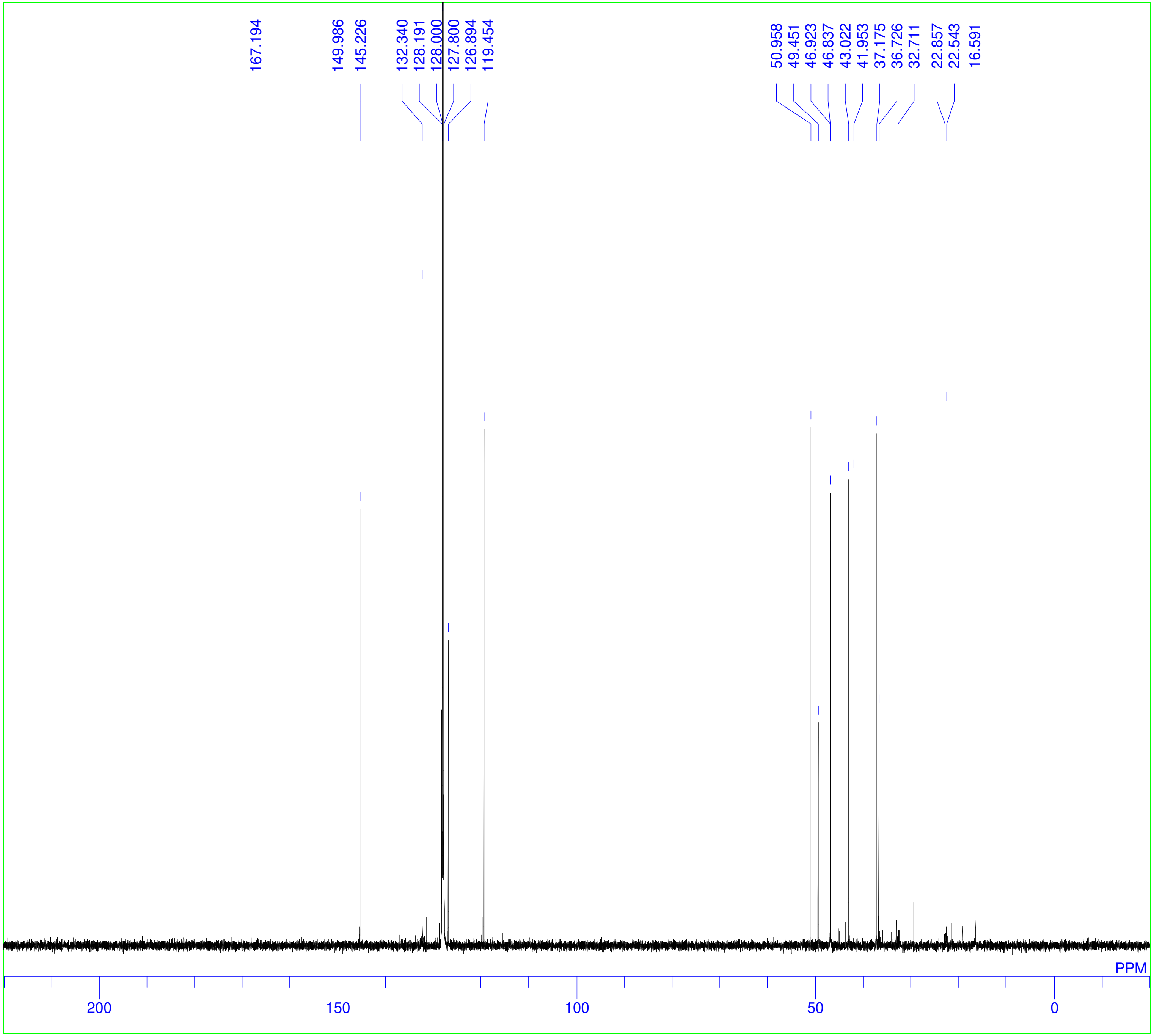

DFILE	5_13C.als
COMNT	
DATIM	2022-09-13 18:39:34
OBNUC	13C
EXMOD	carbon.jxp
OBFRQ	125.77 MHz
OBSET	7.87 KHz
OBFIN	4.21 Hz
POINT	26214
FREQU	31446.54 Hz
SCANS	1024
ACQTM	0.8336 sec
PD	2.0000 sec
PW1	3.87 usec
IRNUC	1H
CTEMP	24.3 c
SLVNT	C6D6
EXREF	128.00 ppm
BF	0.30 Hz
RGAIN	30

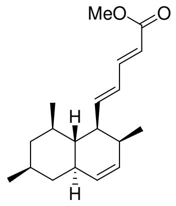

5

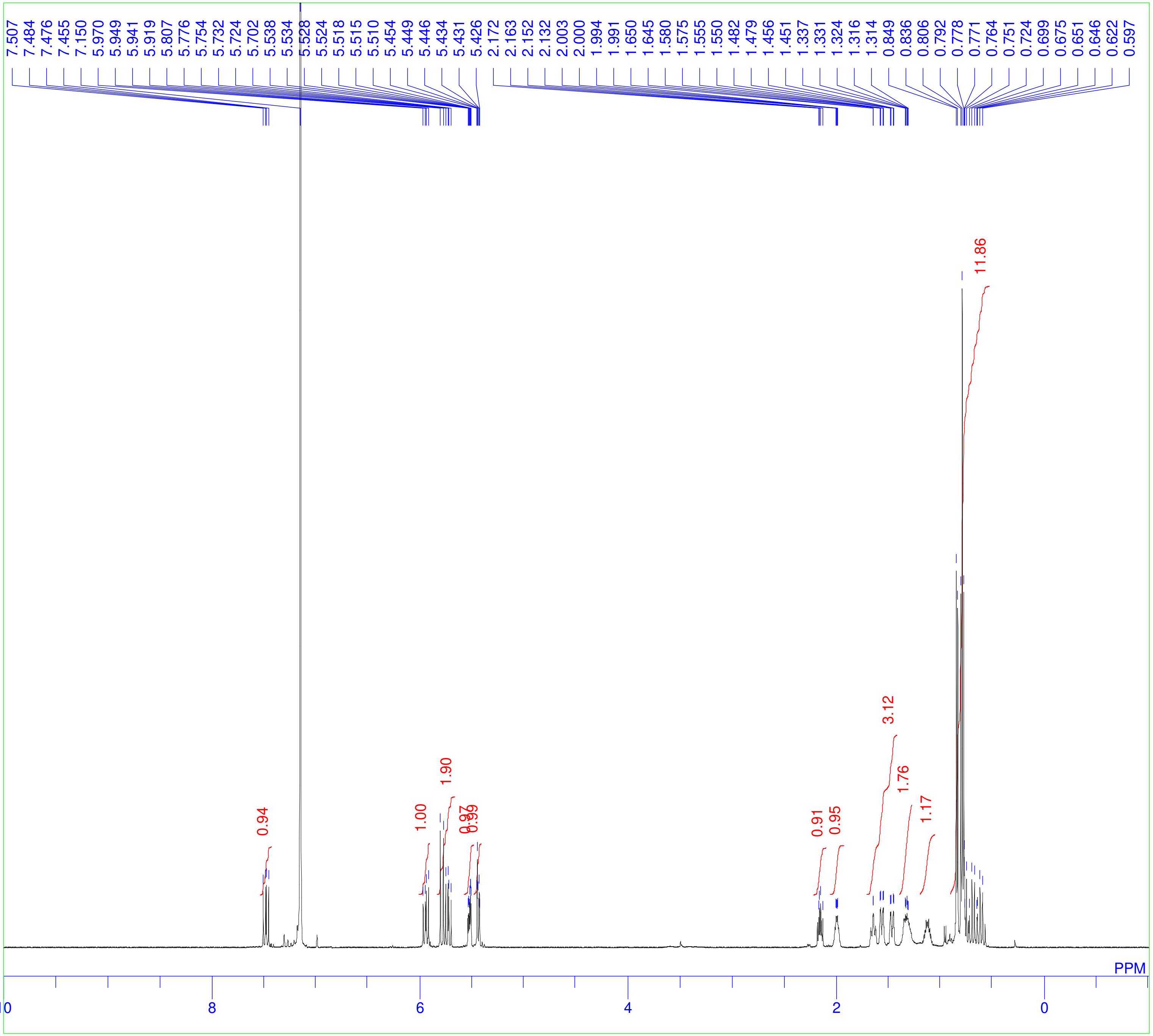

DFILE	Tanzawaic acid B (2)_1H.als
COMNT	
DATIM	2022-09-23 15:00:30
OBNUC	1H
EXMOD	proton.jxp
OBFRQ	500.16 MHz
OBSET	2.41 KHz
OBFIN	6.01 Hz
POINT	13107
FREQU	7507.51 Hz
SCANS	8
ACQTM	1.7459 sec
PD	5.0000 sec
PW1	3.84 usec
IRNUC	1H
CTEMP	23.8 c
SLVNT	C6D6
EXREF	7.15 ppm
BF	0.30 Hz
RGAIN	40

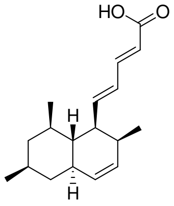

Tanzawaic acid B (2)

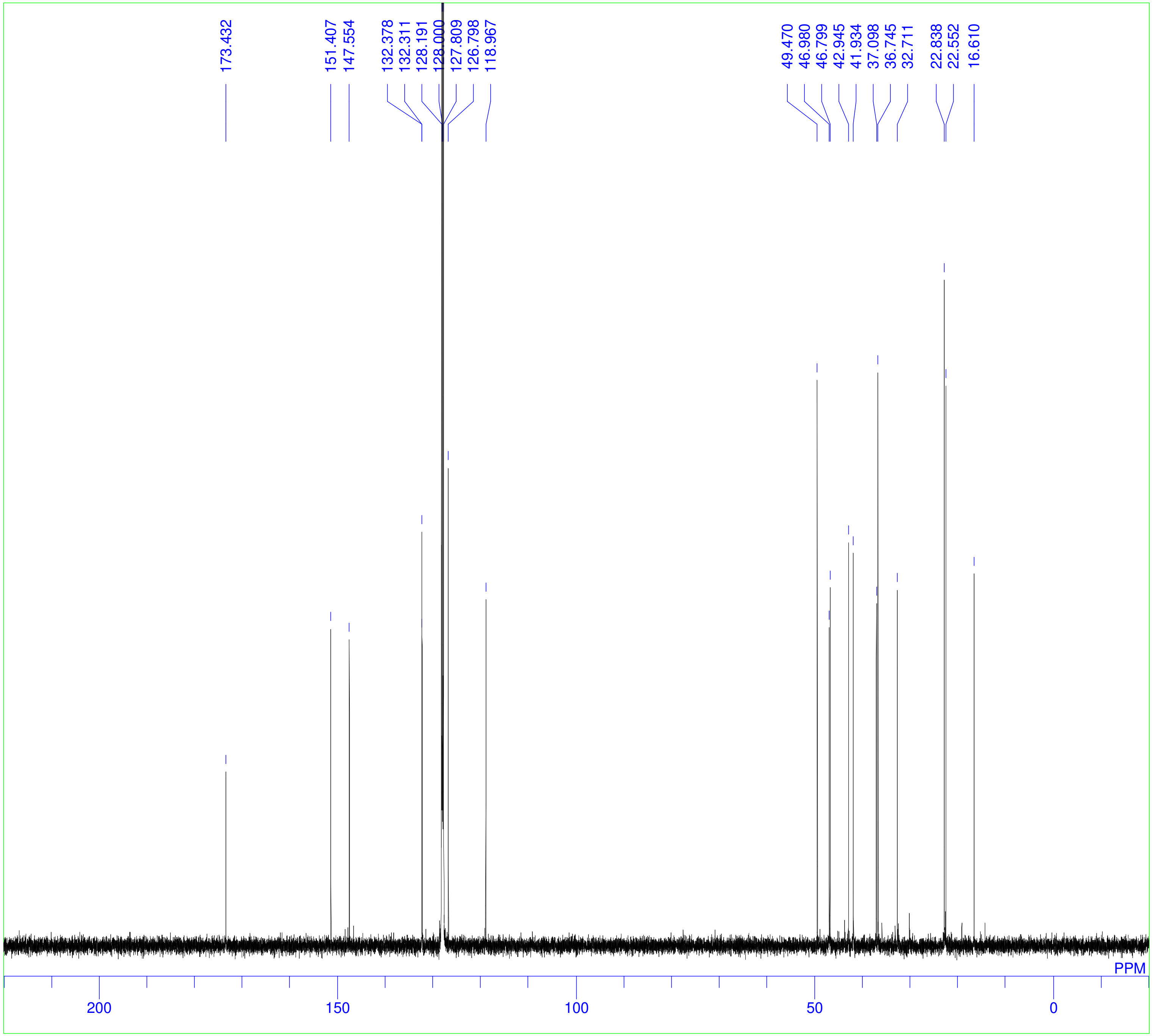

DFILE	Tanzawaic acid B (2)_13C.als
COMNT	
DATIM	2022-09-23 19:18:49
OBNUC	13C
EXMOD	carbon.jxp
OBFRQ	125.77 MHz
OBSET	7.87 KHz
OBFIN	4.21 Hz
POINT	26214
FREQU	31446.54 Hz
SCANS	1024
ACQTM	0.8336 sec
PD	2.0000 sec
PW1	3.87 usec
IRNUC	1H
CTEMP	23.9 c
SLVNT	C6D6
EXREF	128.00 ppm
BF	0.30 Hz
RGAIN	30

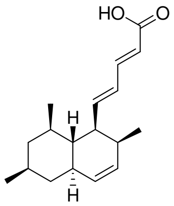

Tanzawaic acid B (2)

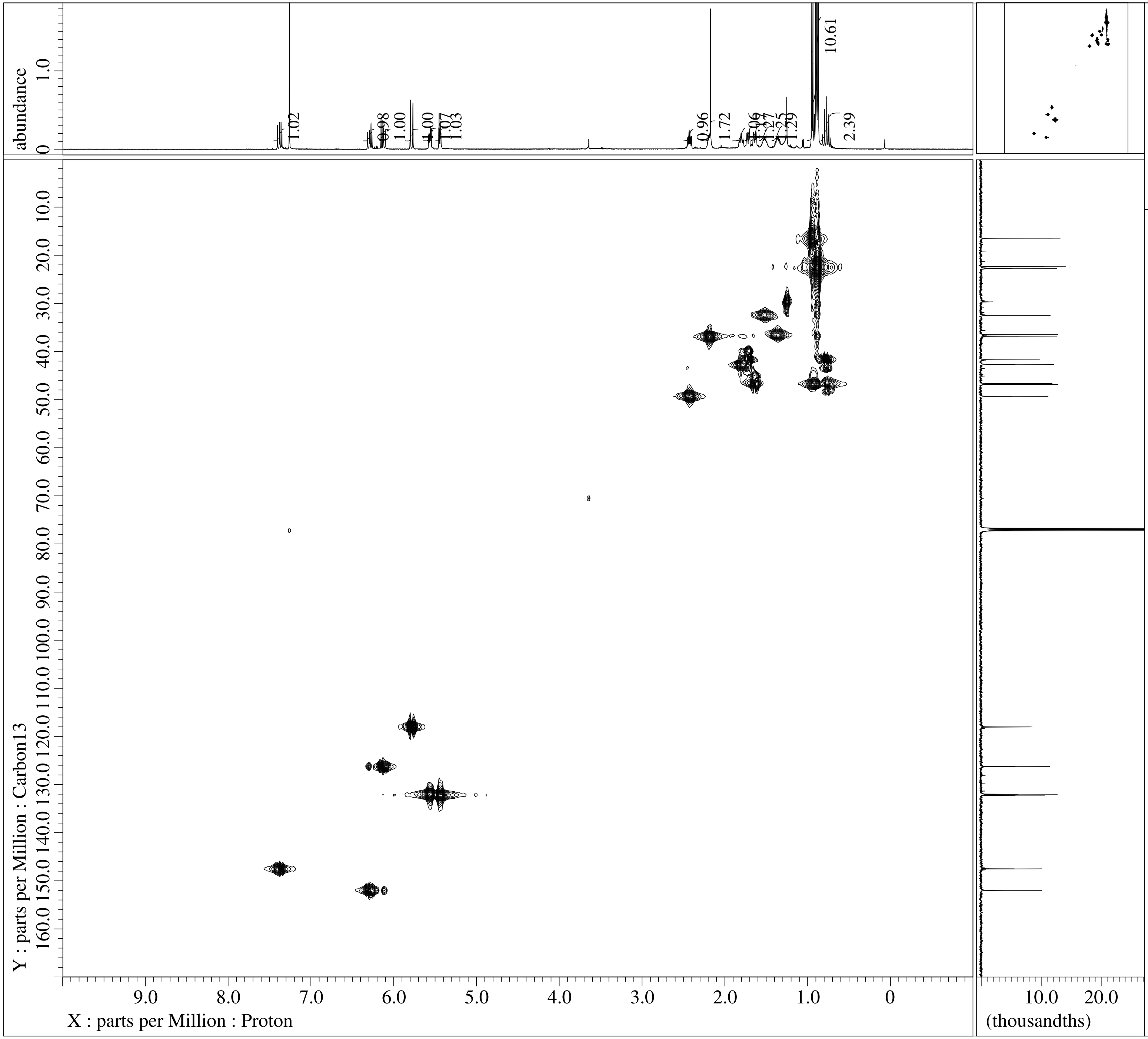


```
---- PROCESSING PARAMETERS ----
sinbell4( -60, 160 )
zerofill( 1 )
fft( 1, TRUE, TRUE )
ppm
[transpose]
sinbell4( -60, 160 )
zerofill( 2 )
fft( 1, TRUE, TRUE )
ppm
abs
```

```
Filename            = Tanzawaic aicd B (2)_HM
Author              = delta
Experiment          = hmqc.jxp
Sample_Id           = HT-1117-TM_overnight
Solvent             = CHLOROFORM-D
Creation_Time       = 14-OCT-2022 00:18:16
Revision_Time       =  2-DEC-2022 21:23:44
Current_Time        =  2-DEC-2022 21:27:16

Comment             = gradient enhanced HMQC
Data_Format         = 2D REAL REAL
Dim_Size            = 819, 512
Dim_Title           = Proton Carbon13
Dim_Units           = [ppm] [ppm]
Dimensions          = X Y
Site                = JNM-ECA500II
Spectrometer        = DELTA2_NMR

Field_Strength      = 11.7473579[T] (500[MHz]
X_Acq_Duration      = 0.10911744[s]
X_Domain            = 1H
X_Freq              = 500.15991521[MHz]
X_Offset            = 5.0[ppm]
X_Points            = 1024
X_Prescans          = 4
X_Resolution        = 9.16443788[Hz]
X_Sweep             = 9.38438438[kHz]
X_Sweep_Clipped     = 7.50750751[kHz]
Y_Domain            = 13C
Y_Freq              = 125.76529768[MHz]
Y_Offset            = 85[ppm]
Y_Points            = 256
Y_Prescans          = 0
Y_Resolution        = 83.60980308[Hz]
Y_Sweep             = 21.40410959[kHz]
Tri_Domain          = Proton
Tri_Freq            = 500.15991521[MHz]
Tri_Offset          = 5.0[ppm]
Clipped             = FALSE
Scans               = 16
Total_Scans         = 4096

Relaxation_Delay    = 1.5[s]
Recvr_Gain          = 50
Temp_Get            = 23.7[dC]
X_Acq_Time          = 0.10911744[s]
X_Atn               = 3.2[dB]
X_Gamma             = 42576375
X_Pulse             = 7.68[us]
```

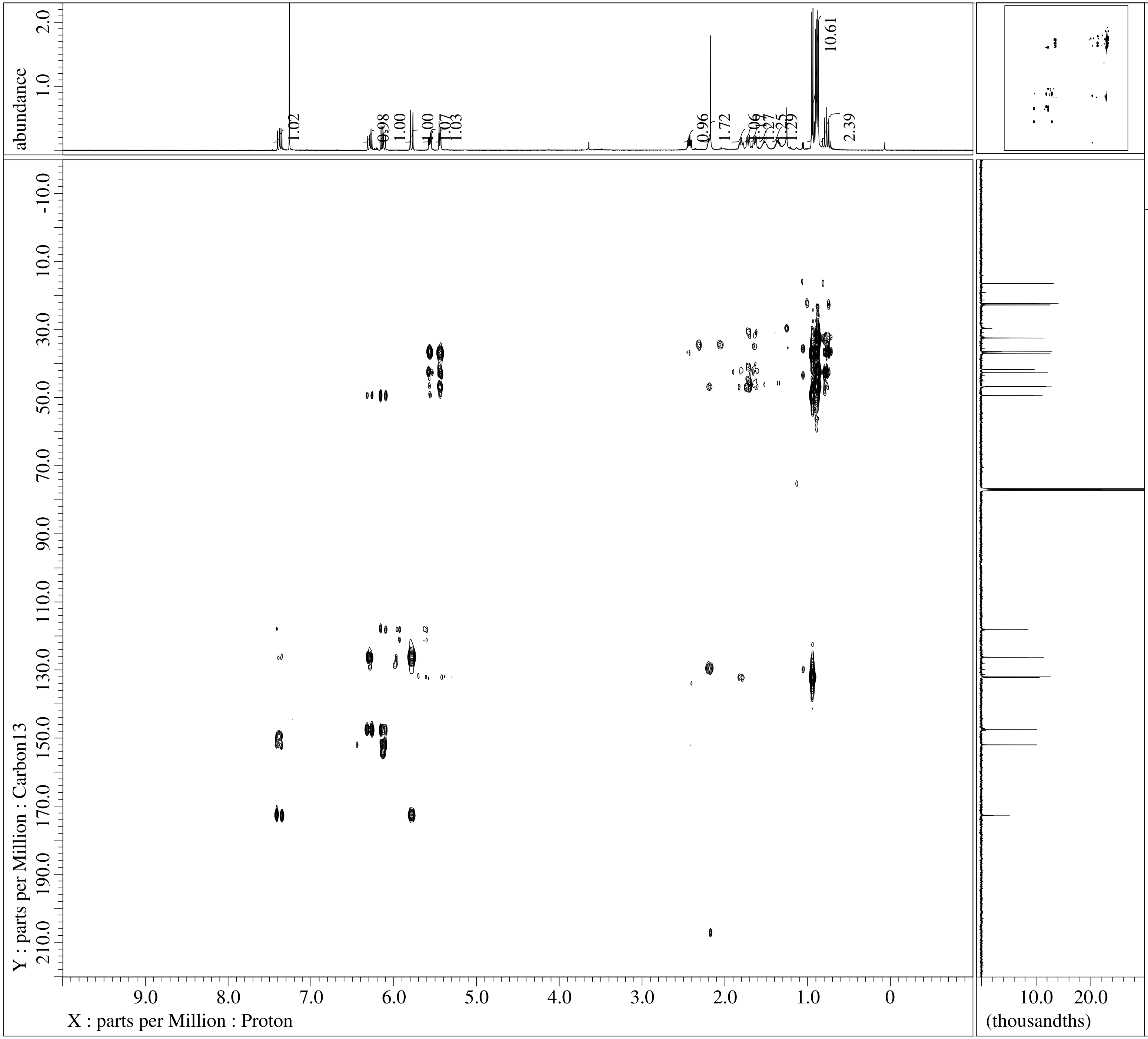

---- PROCESSING PARAMETERS ----
gauss(5.0[Hz], 0.0[s])
sinbell_auto
zerofill(1)
fft(1, TRUE, TRUE)
ppm
[transpose]
sinbell4(-60, 160)
trapezoid(0[%], 5[%], 80[%], 100[%])
zerofill(2)
fft(1, TRUE, TRUE)

Filename = Tanzawaic aicd B (2)_HM
Author = delta
Experiment = hmbc.jxp
Sample_Id = HT-1117-TM_overnight
Solvent = CHLOROFORM-D
Creation_Time = 13-OCT-2022 22:15:34
Revision_Time = 2-DEC-2022 21:14:55
Current_Time = 2-DEC-2022 21:17:00

Comment = gradient enhanced HMBC
Data_Format = 2D REAL REAL
Dim_Size = 1638, 512
Dim_Title = Proton Carbon13
Dim_Units = [ppm] [ppm]
Dimensions = X Y
Site = JNM-ECA500II
Spectrometer = DELTA2_NMR

Field_Strength = 11.7473579[T] (500[MHz]
X_Acq_Duration = 0.21823488[s]
X_Domain = 1H
X_Freq = 500.15991521[MHz]
X_Offset = 5.0[ppm]
X_Points = 2048
X_Prescans = 4
X_Resolution = 4.58221894[Hz]
X_Sweep = 9.38438438[kHz]
X_Sweep_Clipped = 7.50750751[kHz]
Y_Domain = 13C
Y_Freq = 125.76529768[MHz]
Y_Offset = 100[ppm]
Y_Points = 256
Y_Prescans = 0
Y_Resolution = 122.99275819[Hz]
Y_Sweep = 31.4861461[kHz]
Tri_Domain = Proton
Tri_Freq = 500.15991521[MHz]
Tri_Offset = 5.0[ppm]
Clipped = FALSE
Scans = 16
Total_Scans = 4096

Relaxation_Delay = 1.5[s]
Recvr_Gain = 50
Temp_Get = 23.8[dC]
X_Acq_Time = 0.21823488[s]
X_Atn = 3.2[dB]
X_Gamma = 42576375
X_Pulse = 7.68[us]
